# Modulation of NOX2 causes obesity-mediated atrial fibrillation

**DOI:** 10.1172/JCI175447

**Published:** 2024-08-15

**Authors:** Arvind Sridhar, Jaime DeSantiago, Hanna Chen, Mahmud Arif Pavel, Olivia Ly, Asia Owais, Miles Barney, Jordan Jousma, Sarath Babu Nukala, Khaled Abdelhady, Malek Massad, Lona Ernst Rizkallah, Sang-Ging Ong, Jalees Rehman, Dawood Darbar

**Affiliations:** 1 Division of Cardiology,; 2 Department of Pharmacology,; 3 Division of Cardiothoracic Surgery, and; 4 Department of Biochemistry and Molecular Genetics, University of Illinois Chicago, Chicago, Illinois, USA.; 5 Department of Medicine, Jesse Brown Veterans Administration, Chicago, Illinois, USA.

**Keywords:** Cardiology, Arrhythmias

## Abstract

Obesity is linked to an increased risk of atrial fibrillation (AF) via increased oxidative stress. While NADPH oxidase 2 (NOX2), a major source of oxidative stress and reactive oxygen species (ROS) in the heart, predisposes to AF, the underlying mechanisms remain unclear. Here, we studied NOX2-mediated ROS production in obesity-mediated AF using *Nox2*-knockout mice and mature human induced pluripotent stem cell–derived atrial cardiomyocytes (hiPSC-aCMs). Diet-induced obesity (DIO) mice and hiPSC-aCMs treated with palmitic acid (PA) were infused with a NOX blocker (apocynin) and a NOX2-specific inhibitor, respectively. We showed that NOX2 inhibition normalized atrial action potential duration and abrogated obesity-mediated ion channel remodeling with reduced AF burden. Unbiased transcriptomics analysis revealed that NOX2 mediates atrial remodeling in obesity-mediated AF in DIO mice, PA-treated hiPSC-aCMs, and human atrial tissue from obese individuals by upregulation of paired-like homeodomain transcription factor 2 (PITX2). Furthermore, hiPSC-aCMs treated with hydrogen peroxide, a NOX2 surrogate, displayed increased PITX2 expression, establishing a mechanistic link between increased NOX2-mediated ROS production and modulation of PITX2. Our findings offer insights into possible mechanisms through which obesity triggers AF and support NOX2 inhibition as a potential novel prophylactic or adjunctive therapy for patients with obesity-mediated AF.

## Abstract 2

Obesity-mediated atrial fibrillation, driven by NOX2-induced ROS production and modulation of PITX2, is mitigated by NOX2 inhibition, offering potential therapy insights.

## Graphical abstract

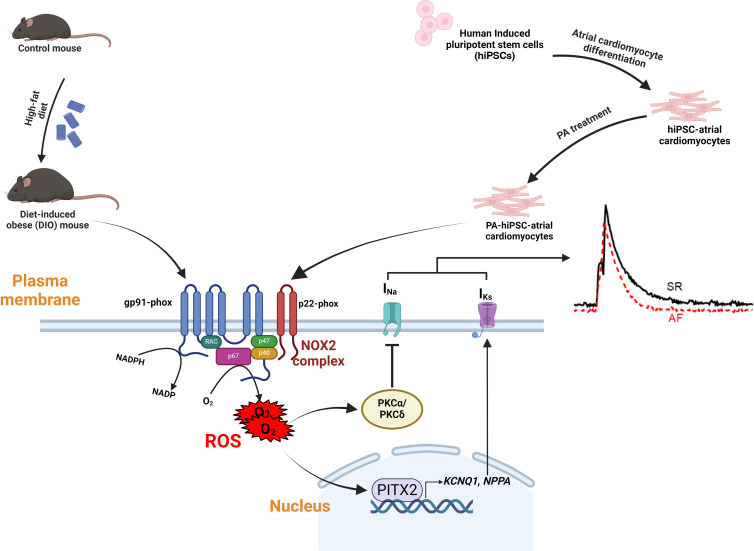

## Introduction

Atrial fibrillation (AF), the most common sustained cardiac arrhythmia, is associated with an increased risk of stroke, heart failure, and death (1). Increasingly, population-based data have identified obesity (body mass index [BMI] ≥ 30 kg/m^2^) as an independent risk factor for AF (2–4). Human and animal studies show that obesity-induced atrial remodeling creates a profibrillatory substrate for AF (4–8). In diet-induced obesity (DIO) mice fed a high-fat diet (HFD), increased AF burden was seen to be mediated by both atrial structural and electrical remodeling (9). Importantly, the atria of DIO mice exhibited reduced cardiac sodium (I_Na_) and calcium currents (I_Ca,L_), while there were enhanced ultra-rapid delayed rectifier current (I_Kur_) and increased fibrosis, leading to a shortening of the atrial action potential duration (APD) and reduced conduction velocity (CV) (9). However, the molecular mechanisms by which obesity mediates AF remain poorly understood.

Oxidative stress and the generation of reactive oxygen species (ROS) play a key role in mediating obesity-induced atrial remodeling and the development of AF (10). Increased lipolysis and the consequent increase in the exposure of myocardial tissue to fatty acids (FAs) result in oxidative injury and inflammation in obese hearts (10–12). The primary sources of ROS production are NADPH oxidase (NOX), mitochondria, xanthine oxidase, cytochrome *c* oxidase, and nitric oxide synthase (13). Recently, NOX2 has emerged as a key player in the pathophysiology of AF with several findings linking the onset of AF to NOX2 upregulation (12–18). Studies have also shown that Nox2 upregulation drives angiotensin II–induced cardiac hypertrophy and fibrosis and ROS production (16). NOX2 is also a critical mediator of AF pathophysiology through its modulation of acetylcholine-activated inward-rectifying potassium current (I_KACh_) via protein kinase C-ε (PKC-ε) translocation to the cell membrane, and NOX2 knockdown reduces the onset of AF in canines (17–19). In DIO mice, increased oxidative stress is specific to both mitochondria and cytoplasm, and MitoTEMPO, a mitochondrial antioxidant, has been shown to reduce structural remodeling and AF burden (9). However, the signaling pathways and mechanisms linking oxidative stress and atrial ion channel and structural remodeling remain unclear.

Most clinical trials using antioxidant therapy to treat AF have failed to show clinical benefit, in part because generic antioxidants such as vitamin C and MitoTEMPO target nonspecific pathways of ROS production (20, 21). Moreover, regardless of the source, ROS generates more ROS in the same way that AF begets AF and facilitates the progression of AF from paroxysmal to persistent forms (10, 12, 13). Thus, the identification and targeting of specific pathways involved in ROS production, such as NOX2, may prevent not only AF but also its progression in obesity-mediated AF.

Human induced pluripotent stem cell–derived atrial cardiomyocytes (hiPSC-aCMs) possess the complex array of ion channels that make up the atrial action potential (AP) and closely mimic the electrical, structural, and metabolic features of human atrial tissue, thus holding great promise for modeling AF (22–25). The most abundant FA found in obese individuals, palmitic acid (PA), increases the expression of NOX2 and the production of mitochondrial ROS in cardiomyocytes (10–12). This can lead to mitochondrial abnormalities and altered calcium homeostasis, and contribute to the development of AF in obesity (12). Thus, treating hiPSC-aCMs with PA creates an extracellular milieu that resembles obese human atria.

Altered expression of paired-like homeodomain transcription factor 2 (PITX2), which has been associated with the chromosome 4q25 locus in AF patients, results in abnormal atrial electrical properties in both humans and mice, highlighting its significance in the pathophysiology of AF (26). Investigating the pro-arrhythmic effects of *PITX2*-induced electrical remodeling is a crucial step toward understanding and treating AF (26, 27). However, the specific role of *PITX2* in obesity-mediated AF and increase in NOX2 remains unclear. Considering the link between NOX2 and atrial remodeling, we hypothesized that NOX2 drives oxidative stress and ROS, resulting in atrial channel and structure changes via modulation of PITX2 in obesity-mediated AF (15–19). To test this, we used a *Nox2*-knockout (KO) mouse model and PA-treated hiPSC-aCMs treated with apocynin (NOX blocker) (28, 29) and GSK-2795039 (NOX2 inhibitor) (30). Collectively, both genetic and pharmacologic inhibition of NOX2 in obese mice and PA-treated hiPSC-aCMs abrogates ion channel and structural remodeling and prevents obesity-mediated AF in part by transcriptional regulation of *PITX2*.

## Results

### NOX2 is increased in atrial tissue of obese individuals.

**Figure 1:**
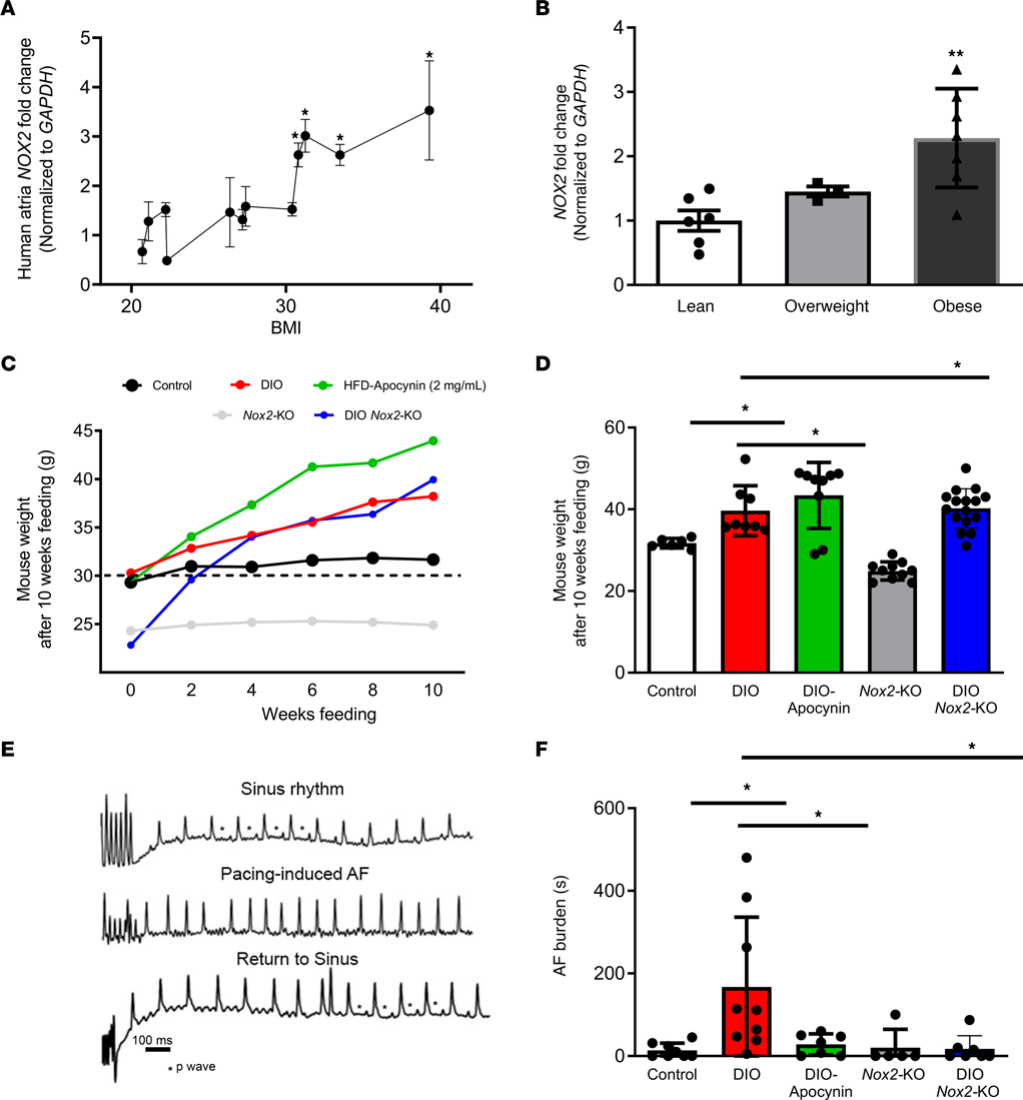
Genetic and pharmacological inhibition of NADPH oxidase 2 (NOX2) reduces obesity-mediated AF. (**A**) Human *NOX2* mRNA expression versus patient BMI (kg/m^2^). (**B**) Human *NOX2* mRNA expression in lean (*n* = 6), overweight (*n* = 3), and obese individuals (*n* = 7). (**C**) Average weight (grams) of control, diet-induced obesity (DIO), DIO-apocynin, *Nox2*-knockout (KO), and DIO *Nox2*-KO mice over 10-week duration with an HFD. (**D**) Final weights (grams) of all 5 groups of mice after 10 weeks of HFD. (**E**) Atrial electrograms showing sinus rhythm at baseline (top), pacing-induced AF in DIO mice (middle), and sinus rhythm restoration in DIO mice (bottom). (**F**) Pacing-induced AF burden (duration, seconds) in control (*n* = 8), DIO (*n* = 9), DIO-apocynin (*n* = 7), *Nox2*-KO (*n* = 5), and DIO *Nox2*-KO (*n* = 7) mice. *P* > 0.05, *P < 0.05, **P < 0.01, by 2-tailed, unpaired Student’s *t* test.

As NOX2 protein is increased in obesity-mediated AF in DIO mice (9), we examined NOX2 levels in human atrial tissue of obese individuals with real-time quantitative PCR (qPCR). Individuals were grouped into lean (BMI 18.5–25 kg/m^2^), overweight (BMI 25 to <30 kg/m^2^), and obese (BMI >30.0 kg/m^2^). BMI was the only key differentiator that was significantly changed in the overweight and obese groups compared with lean. Notably, other clinical parameters such as age, ejection fraction, left atrial size, and prevalence of conditions like diabetes mellitus, hypertension, coronary artery disease, and congestive heart failure did not exhibit statistically significant differences across the groups (Supplemental Table 1; supplemental material available online with this article; https://doi.org/10.1172/JCI175447DS1). We observed increased mRNA expression of human atrial *NOX2* in individuals with BMI greater than 30 kg/m^2^ (Figure 1A). While there was no significant change in *NOX2* expression among overweight individuals, obese individuals displayed a more than 2-fold increase in atrial *NOX2* expression as compared with lean individuals (Figure 1B).

We then evaluated the expression of cardiac ion channels and structural genes. Though not statistically significant, obese individuals displayed a marked increase in the mRNA expression of *KCNA5* compared with lean individuals (Supplemental Figure 1A). Overweight and obese individuals also displayed a decrease in the mRNA expression of *GJA5*, which encodes connexin 40, of which the decrease in overweight patients was statistically significant (Supplemental Figure 1B). In contrast, *SCN5A* encoding I_Na_ was not significantly changed in comparison with lean individuals (Supplemental Figure 1C). *CACNA1C* encoding I_Ca,L_ showed a marked increase in obese individuals as compared with lean individuals (Supplemental Figure 1D).

### NOX2 inhibition prevents obesity-mediated AF.

Given the association between NOX2 and atrial ion channel remodeling in obesity-mediated human AF, we investigated whether genetic knockout of NOX2 or pharmacological inhibition would reduce AF burden in an animal model of obesity, diet-induced obesity (DIO). We fed control (C57BL/6J, DIO) and *Nox2-*KO male mice a 60% HFD for 10 weeks. Female mice were omitted from the study because of their heightened resistance to the obesogenic effects of HFD. Only obese mice that weighed more than 33 g were included in the study. Some DIO mice were given a pharmacological NOX2 inhibitor, apocynin, in the drinking water (2 mg/mL; DIO-apocynin) (28, 29). We then assessed AF incidence and burden using transesophageal atrial burst pacing as we previously described (9). DIO, DIO *Nox2*-KO, and DIO-apocynin mice increased their weight compared with lean controls and *Nox2*-KO mice fed with the control diet (Figure 1C). The average weights of DIO, DIO-apocynin, and DIO *Nox2*-KO mice were substantially increased compared with those of control and *Nox2*-KO mice (39.6 ± 6.1 g, 43.4 ± 8.1 g, and 40.2 ± 4.8 g, respectively, vs. 31.7 ± 1.2 g and 24.9 ± 2.2 g, respectively) (Figure 1D). In our study, following transesophageal atrial pacing, we observed that both DIO *Nox2*-KO and DIO-apocynin mice had a markedly reduced AF burden compared with DIO mice. DIO *Nox2*-KO mice and DIO-apocynin mice had 17.4 ± 31.8 seconds and 28.3 ± 25.4 seconds of AF compared with 167.3 ± 168.9 seconds in DIO mice, respectively (Figure 1, E and F). Compared with DIO mice, DIO *Nox2*-KO mice showed a reduction in AF incidence, while the change was not statistically significant in DIO-Apocynin mice (Supplemental Figure 1E). Collectively, our data show that both genetic and pharmacological inhibition of NOX2 expression prevents pacing-induced AF in DIO mice.

### Genetic suppression of Nox2 reverses obesity-mediated AF by normalizing atrial APD.

**Figure 2:**
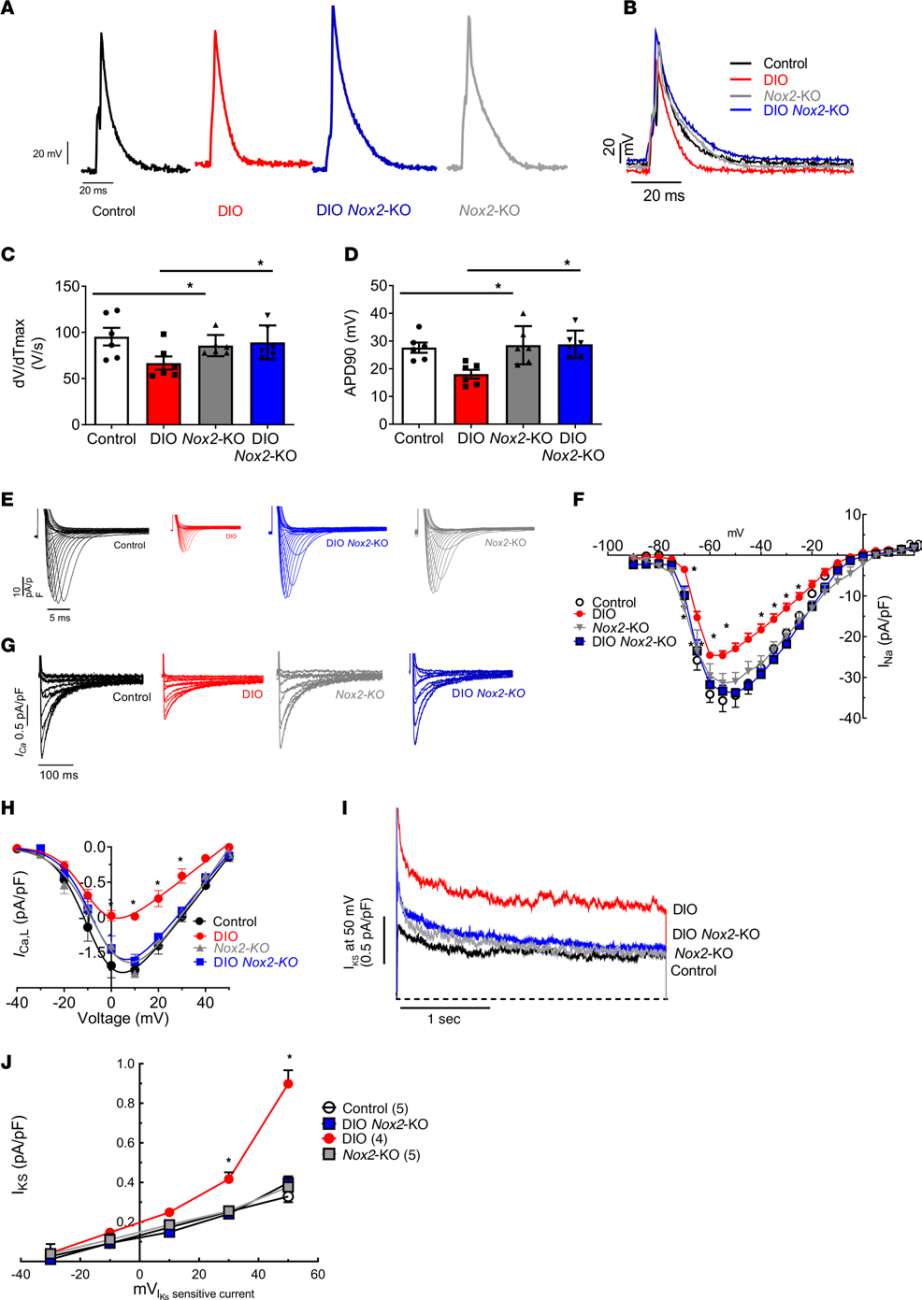
DIO *Nox2*-KO mice display increased atrial action potential and abrogation of obesity-induced ion channel remodeling. (**A** and **B**) Whole-cell patch clamping of atrial myocytes of DIO *Nox2*-KO mice showed increased prolongation of shortened action potential duration (APD) caused by obesity. Representative AP tracings in atrial myocytes in control (*n* = 6 cells, *n* = 4 mice), DIO (*n* = 6 cells, *n* = 4 mice), *Nox2*-KO (*n* = 6 cells, *n* = 3 mice), and DIO *Nox2*-KO mice (*n* = 6 cells, *n* = 3 mice). (**C**) Instantaneous rate of voltage change over time (dV/dT_max_), an indicator of atrial conduction velocity (CV; *n* = 6 cells). (**D**) Measured APD at 90% repolarization (APD90; *n* = 6 cells). (**E**) Representative sodium current (I_Na_) tracings from control, DIO, and DIO *Nox2*-KO mice showing increased currents in DIO *Nox2*-KO atrial myocytes (*n* = 6 atrial cells, *n* = 3 mice). (**F**) I_Na_ and voltage relationship (I-V curves) in control (*n* = 6), DIO (*n* = 6), and DIO *Nox2*-KO mice (*n* = 6). (**G**) Representative L-type calcium current (I_Ca,L_) tracings from control, DIO, and DIO *Nox2*-KO mice showing increased currents in DIO *Nox2*-KO atrial myocytes (*n* = 4 cells, *n* = 3 mice). (**H**) I_Ca,L_ and voltage relationship (I-V curves) in control (*n* = 4), DIO (*n* = 4), *Nox2*-KO (*n* = 4), and DIO *Nox2*-KO mice (*n* = 4). (**I**) Slow delayed rectifier potassium current (I_Ks_; treated with 1 μM HMR-1556) and voltage relationship (I-V curves) in control (*n* = 5 cells, *n* = 3 mice), DIO (*n* = 4 cells, *n* = 3 mice), *Nox2*-KO (*n* = 7 cells, *n* = 3 mice), and DIO *Nox2*-KO mice (*n* = 8 cells, 3 mice). (**J**) Comparison of I_Ks_ at 50 mV in control (*n* = 5 cells), DIO (*n* = 4 cells), *Nox2*-KO (*n* = 7 cells), and DIO *Nox2*-KO mice (*n* = 8 cells). *P* > 0.05, *P < 0.05, by 1-tailed ANOVA with Bonferroni’s post hoc test for multiple comparisons.

To assess electrophysiological impact of Nox2 deletion, we used whole-cell patch clamping in freshly isolated atrial cardiomyocytes pooled from both left atrium (LA) and right atrium (RA) from control, DIO, *Nox2*-KO, and DIO *Nox2*-KO mice. First, DIO *Nox2*-KO mice displayed a prolonged atrial AP compared with DIO mice with substantial normalization of the action potential duration at 20% repolarization (APD20), APD50, and APD90 (*P* < 0.05; Figure 2, A and B, and Supplemental Figure 2, A and B). Second, both the maximum AP amplitude (APA_max_) and the upstroke velocity (dV/dT_max_) were significantly increased in DIO *Nox2*-KO mice compared with DIO mice (*P* < 0.05; Figure 2C and Supplemental Figure 2B). Third, the atrial AP of DIO *Nox2*-KO mice closely resembled that of control and *Nox2*-KO mice, and there were no changes in the APD20, APD50, APD90, APA_max_, and dV/dT_max_ between the 3 groups (Figure 2, A–D, and Supplemental Figure 2, A, B, and D). There were no changes to the resting membrane potential across the 4 groups of mice (Supplemental Figure 2C).

### DIO Nox2-KO mice restore APD by modulating I_Na_ and I_Ks_.

Inducible AF in obese mice is mediated in part by ion channel remodeling of both I_Na_ and slow delayed rectifier potassium current (I_Ks_) (9). To determine whether NOX2 protein inhibition restores atrial I_Na_, we performed whole-cell voltage patch clamping in all 4 groups of mice. DIO *Nox2*-KO mice showed a significant increase in peak I_Na_ density when compared with DIO mice (Supplemental Figure 3, A–C) with the restoration of I_Na_ densities at all test potentials, similarly to control and *Nox2*-KO mice (Figure 2, E and F). There was also increased protein expression of Na_v_1.5 in DIO *Nox2*-KO and DIO-apocynin mice compared with DIO mice (Supplemental Figure 3, D–F). Nox2 increase leads to increased protein levels of PKC isoforms (31, 32). Increased PKC-δ activity reduces overall Na_v_1.5 expression and decreases I_Na_ (31, 32). We performed Western blots on control, DIO, *Nox2*-KO, and DIO *Nox2*-KO mice and showed that knockout of *Nox2* reversed the obesity-induced increase in the protein-level expression of PKC-α and PKC-δ isoforms (Supplemental Figure 3, G and H).

Voltage clamping studies revealed that increased total I_K_ in DIO mice was reduced significantly in DIO *Nox2*-KO mice (Supplemental Figure 4A). Using HMR-1556 to quantify I_Ks_ indicated that NOX2 inhibition abrogated obesity-induced I_Ks_ in DIO *Nox2*-KO (Figure 2I). I_Ks_ densities at 30 mV and 50 mV were substantially reduced in DIO *Nox2-*KO mice versus DIO mice (Figure 2, I and J). We then evaluated the mRNA and protein levels of several genes encoding the α and β subunits of the potassium channels with roles in AP repolarization. DIO *Nox2*-KO mice displayed decreased K_v_7.1 and MinK protein expression compared with DIO mice; however, K_v_1.5, another major potassium channel involved in AF, remained unchanged (Supplemental Figure 4, C–E). DIO *Nox2*-KO mice showed reduced mRNA expression of *Kcnq1* and *Kcne1*, which encode I_Ks_, and *Kcna5*, which encodes I_Kur_ (Supplemental Figure 4B). Lastly, gene and protein expression of *Kcnj3*, which encodes the inward-rectifying potassium channel Kir3.1 and forms a part of the acetylcholine-activated potassium channel (I_KACh_), was significantly reduced in DIO *Nox2*-KO mice as compared with DIO mice (Supplemental Figure 4, B and G). We previously showed that increased atrial natriuretic peptide (ANP) activity modulates I_Ks_ in hiPSC-aCMs harboring an *NPPA* mutation (23, 33). mRNA and protein expression of ANP was markedly increased in DIO mice as compared with controls, but the increase was abrogated in DIO *Nox2*-KO mice (Supplemental Figure 4, B and G).

### NOX2 inhibition improves contractility in DIO mice and PA-treated hiPSC-aCMs.

**Figure 3:**
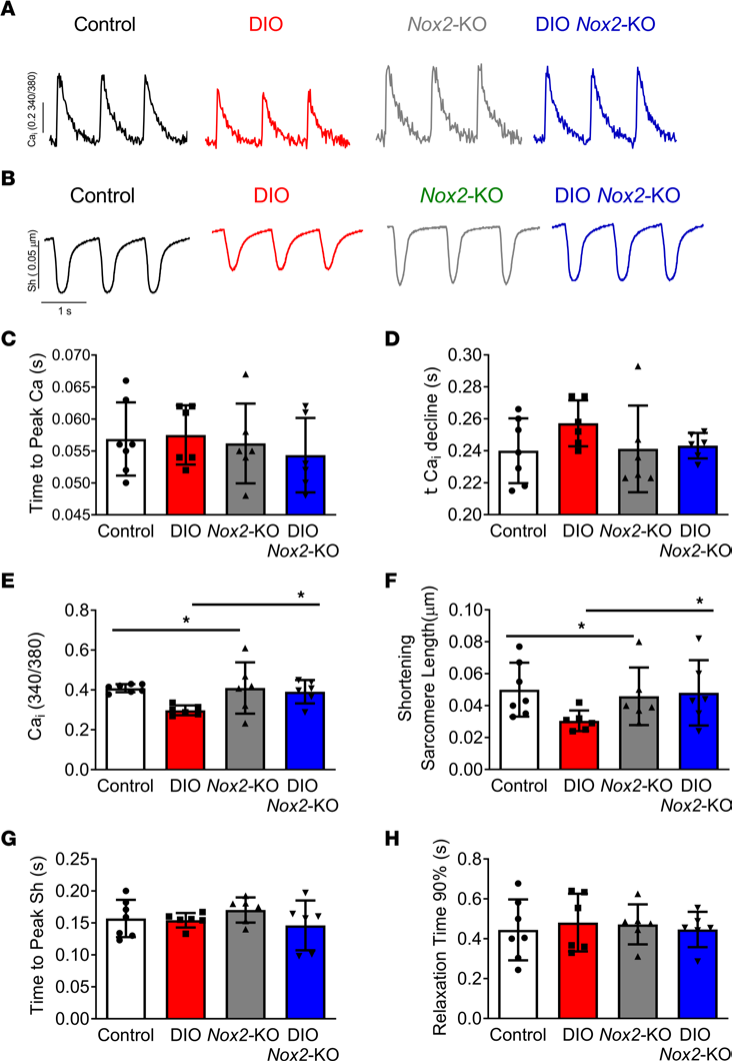
NOX2 inhibition improves atrial contractility in DIO mice. (**A**) Representative calcium transient tracings from control, DIO, *Nox2*-KO, and DIO *Nox2*-KO atrial myocytes. (**B**) Representative cell shortening tracings from control, DIO, *Nox2*-KO, and DIO *Nox2*-KO atrial myocytes (*n* = 6 cells, *n* = 3 mice each). (**C**–**E**) Quantification of calcium transient tracings. (**C**) Time to peak calcium. (**D**) Time for calcium decline. (**E**) Calcium transient peak amplitudes. (**F**–**H**) Quantification of sarcomere cell shortening tracings. (**F**) Shortening sarcomeric length. (**G**) Time to peak cell shortening. (**H**) Time to 90% relaxation (*n* = 6 cells.) *P < 0.05.

Reduction of the density of the I_Ca,L_, which is generated by channels composed of Ca_v_1.2 (encoded by *CACNA1C*), β_2_ (*CACNB2*), and α_2_δ (*CACNA2D*) subunits, is a hallmark of the atrial electrical remodeling in DIO mice (9). The reduction in I_Ca,L_ induces a reduction in calcium release from the sarcoplasmic reticulum (SR) and a reduction in overall atrial contractility. To further evaluate this change, we used voltage clamping studies on pooled mouse atrial cardiomyocytes to measure I_Ca,L_ along with calcium transient measurements using the fluorescent calcium dye Fura-2 in DIO *Nox2*-KO mice (Figure 2, G and H, and Figure 3, A–H). I_Ca, L_ reduction in DIO mice was markedly reversed in DIO *Nox2*-KO mice (Figure 2, G and H). We also observed a reduction of intracellular Ca^2+^ ([Ca^2+^]_i_) in DIO atrial cells, which was reversed in DIO *Nox2*-KO atrial cells, thus highlighting an increased magnitude of calcium release from the sarcoplasmic reticulum in DIO *Nox2*-KO mice (Figure 3E). DIO atrial cells also showed a substantial decrease in sarcomeric cell shortening, a measure of atrial contractility, compared with control and DIO *Nox2*-KO atrial cells (Figure 3F). There were no statistical differences in the time to peak and relaxation time between the 4 groups (Figure 3, C, D, G, and H).

### NOX2 inhibition in PA-treated hiPSC-aCMs using NOX2 small-molecule inhibitor reverses obesity-induced ion channel remodeling.

**Figure 4:**
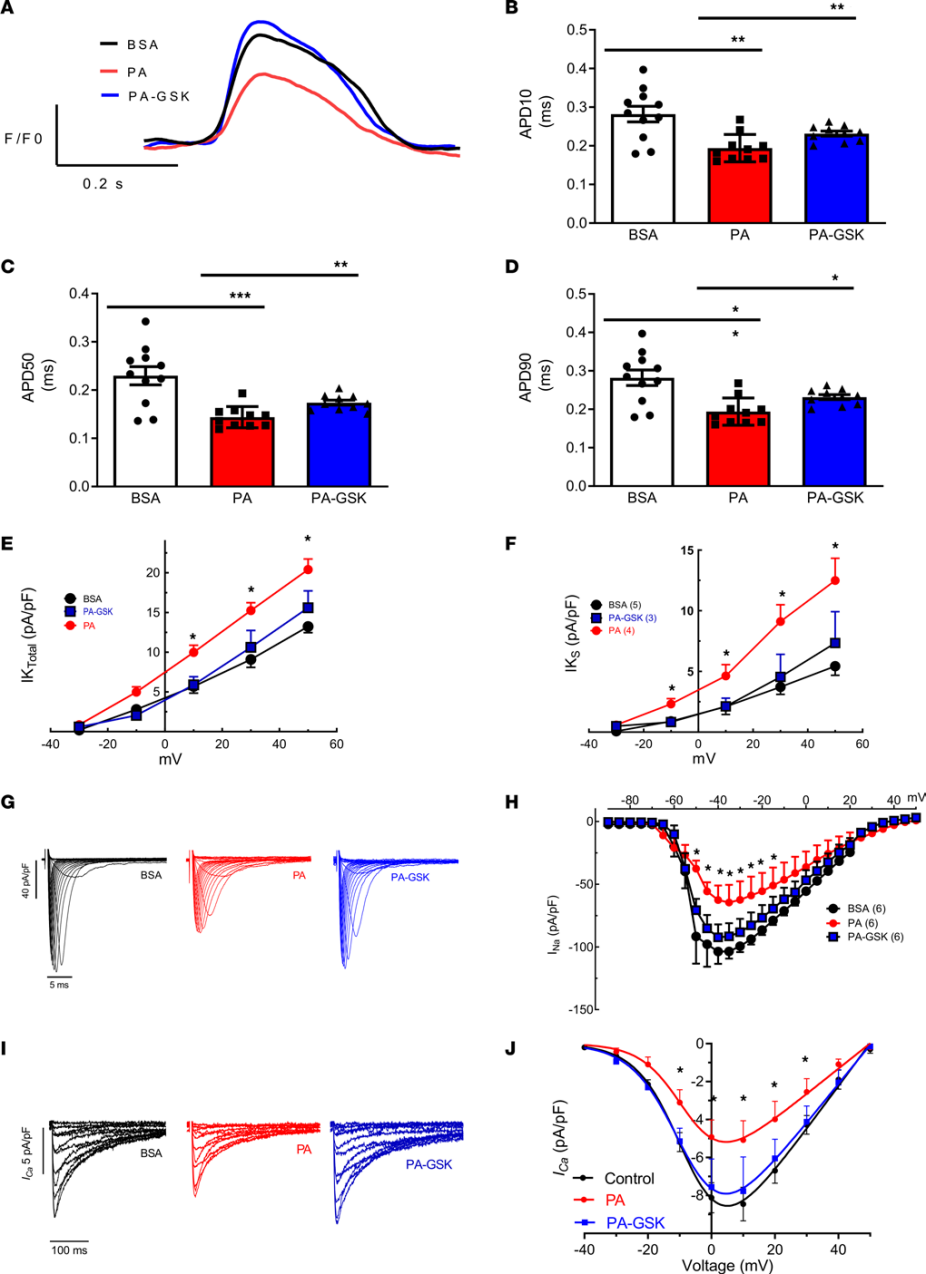
NOX2 inhibition in PA-treated hiPSC-aCMs using the NOX2 small-molecule inhibitor GSK-2795039 reverses obesity-induced ion channel remodeling. (**A**) Optical voltage mapping on vehicle BSA-, PA-, and PA-GSK-hiPSC-aCMs showed that the shortening of atrial AP duration observed in PA-hiPSC-aCMs is abrogated in PA-GSK-hiPSC-aCMs. (**B**) Measured APD at 10% repolarization (APD10). (**C**) Measured APD at 50% repolarization (APD50). (**D**) Measured APD at 90% repolarization (APD90). (**E**) Total potassium current (I_K_) and voltage relationship (I-V curves) in BSA- (*n* = 5), PA- (*n* = 4), and PA-GSK-hiPSC-aCMs (*n* = 3). (**F**) Slow delayed rectifier potassium current (I_Ks_) and voltage relationship (I-V curves) in BSA- (*n* = 5), PA- (*n* = 4), and PA-GSK-hiPSC-aCMs (*n* = 3). (**G**) Representative I_Na_ traces in BSA- (*n* = 6), PA- (*n* = 6), and PA-GSK-hiPSC-aCMs (*n* = 6). (**H**) Peak I_Na_ current density in BSA- (*n* = 6), PA- (*n* = 4), and PA-GSK-hiPSC-aCMs (*n* = 3). (**I**) Representative I_Ca,L_ traces in BSA- (*n* = 6), PA- (*n* = 6), and PA-GSK-hiPSC-aCMs (*n* = 6). (**J**) Peak I_Ca,L_ current density in BSA- (*n* = 6), PA- (*n* = 5), and PA-GSK-hiPSC-aCMs (*n* = 4). *P < 0.05, **P < 0.01, ***P < 0.001, by 1-tailed ANOVA with Bonferroni’s post hoc test for multiple comparisons.

Retinoic acid was used specifically to induce hiPSC-aCM differentiation from hiPSCs. Flow cytometry revealed a significant increase in the percentage of cells expressing K_v_1.5 in retinoic acid–treated cells compared with DMSO-treated cells, while there was a decrease in the percentage of cells expressing MLC2v, a ventricular marker (Supplemental Figure 11, A–D). We used mature hiPSC-aCMs treated with PA and a NOX2 small-molecule inhibitor, GSK-2795039 (20 μM dissolved in DMSO; PA-GSK-hiPSC-aCMs), and oleic acid (OA; at 0.5 μM) for 5 days to study the effects of NOX2 in mediating FA-induced atrial remodeling. Optical voltage mapping experiments on PA-GSK-hiPSC-aCMs showed results similar to those in DIO *Nox2*-KO mice with a reversal in shortened AP in PA-GSK-hiPSC-aCMs compared with PA-treated hiPSC-aCMs (PA-hiPSC-aCMs) at 10%, 50%, and 90% repolarization (Figure 4, A–D). As in DIO mice, hiPSC-aCMs treated with PA also displayed increased I_Ks_ and total I_K_, which was reversed in PA-GSK-hiPSC-aCMs (Figure 4, E and F). Whole-cell patch clamping studies revealed that chronic PA treatment shortened APD at 20%, 50%, and 90% repolarization while OA treatment markedly prolonged APD50 and increased the maximum upstroke velocity (Supplemental Figure 7, A–E). PA-hiPSC-aCMs in contrast to bovine serum albumin (BSA)–hiPSC-aCMs showed a marked reduction in the maximum upstroke velocity and maximum amplitude of the AP (APA_max_) (Supplemental Figure 7, E and F). Moreover, PA-hiPSC-aCMs also showed decreased I_Na_ and I_Ca,L_ densities compared with control hiPSC-aCMs, which was reversed in PA-GSK-hiPSC-aCMs (Figure 4, G–J). Thus, our results suggest that genetic deletion and pharmacological inhibition of NOX2 abrogate atrial APD shortening mediated by obesity and rescue obesity-induced AF in both DIO *Nox2*-KO mice and PA-GSK-hiPSC-aCMs.

Echocardiographic analyses showed that DIO mice displayed increased LA size compared with control mice (Supplemental Figure 5, A and C, and Supplemental Table 2). While DIO mice showed both LA and RA enlargement compared with controls, only LA size was abrogated in DIO *Nox2*-KO mice (Supplemental Figure 5C). There was no notable difference in the right ventricular area between the 4 groups of mice. Other echocardiographic parameters such as the left ventricular ejection fraction, fractional shortening, pulse wave ratio between active and passive ventricle filling (A′/E′), cardiac output, and left ventricular posterior wall diameter were unchanged across the 4 groups of mice (Supplemental Figure 5, B, D, and E, and Supplemental Table 2).

### NOX2 inhibition prevents obesity-mediated atrial fibrosis and increases atrial CV.

**Figure 5:**
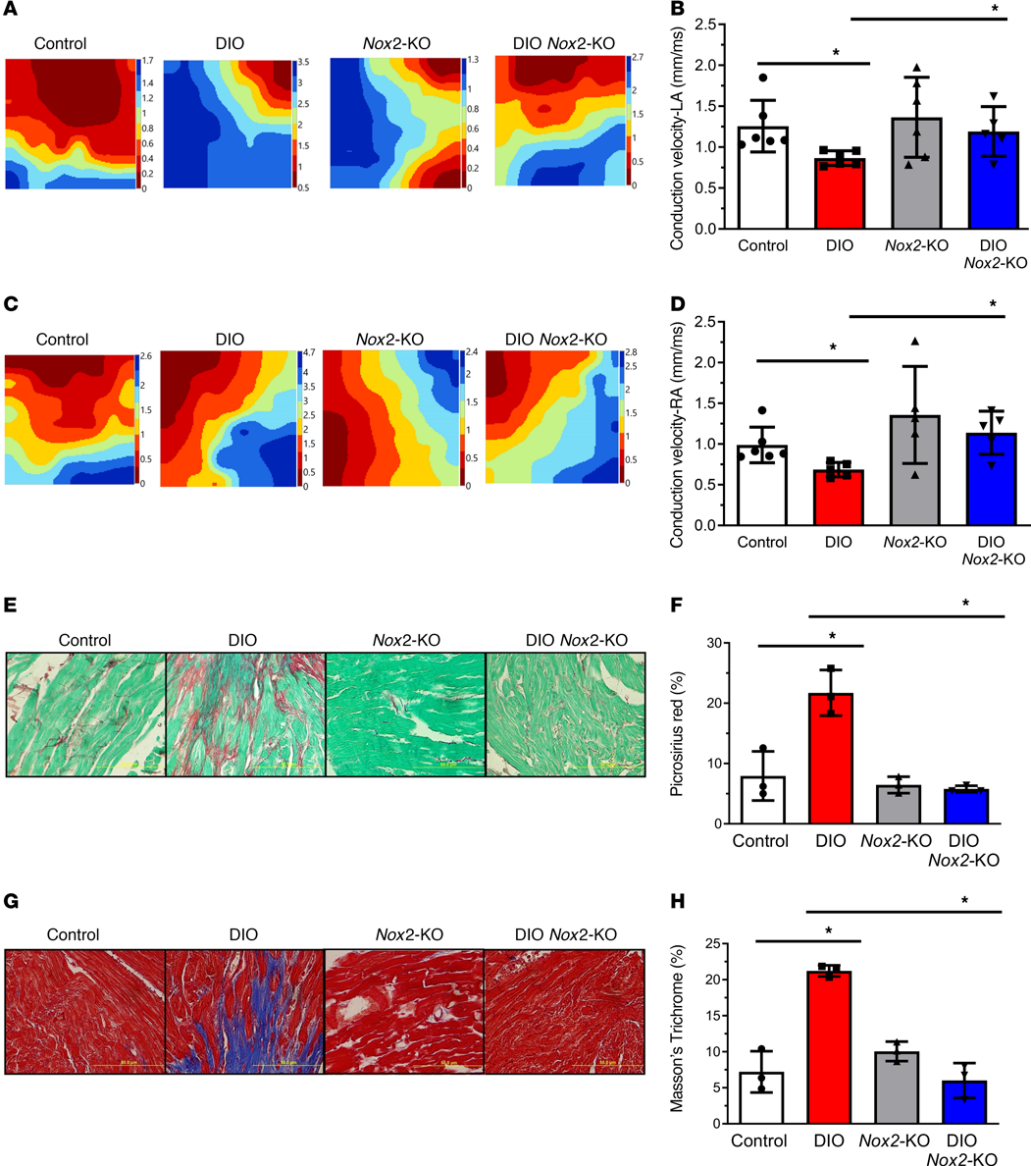
NOX2 inhibition prevents atrial fibrosis and increases atrial CV in DIO mice. (**A**) Representative isochronal maps of the LA in the 3 groups of mice using electrical mapping. (**B**) Quantification of mean LA CV in control (*n* = 6), DIO (*n* = 6), *Nox2*-KO (*n* = 5), and DIO *Nox2*-KO mice (*n* = 6). (**C**) Representative isochronal maps of the RA in the 3 groups of mice using electrical mapping. (**D**) Quantification of mean RA CV in control (*n* = 6), DIO (*n* = 6), *Nox2*-KO (*n* = 5), and DIO *Nox2*-KO mice (*n* = 6). (**E**) Picrosirius red staining of atrial myocytes from control, DIO, *Nox2*-KO, and DIO *Nox2*-KO. (**F**) Change in fibrosis (%) in the 3 groups of mice showing a significant reduction in fibrosis in DIO *Nox2*-KO compared with DIO mice (*n* = 3 mice each). Scale bar: 50 μm. (**G**) Masson’s trichrome staining of atrial myocytes from control, DIO, and DIO *Nox2*-KO. Scale bar: 50 μm. (**H**) Change in fibrosis (%) in the 4 groups of mice showing a significant reduction in fibrosis in DIO *Nox2*-KO compared with DIO mice (*n* = 3 mice each). *P < 0.05, **P < 0.01, by 2-tailed, unpaired Student’s *t* test.

To study changes in conduction, epicardial multielectrode array mapping of both atria and the left ventricle was performed in isolated Langendorff-perfused beating hearts from all 4 mouse groups. The CVs were reduced in both atria and the left ventricle in DIO, *Nox2*-KO, and DIO *Nox2*-KO mice compared with controls. However, DIO *Nox2*-KO mice showed improved LA and RA and left ventricular CV compared with DIO mice (Figure 5, A–D, and Supplemental Figure 6, B and C). The isochronal maps also illustrated a pattern consistent with improved CV in both atria of DIO *Nox2*-KO mice compared with DIO mice (Figure 5, A–D). We then determined whether NOX2 inhibition reduces obesity-induced atrial fibrosis by using Picrosirius red and Masson’s trichrome staining on histological sections from control, DIO, and DIO *Nox2*-KO mice. LA and RA sections from DIO mice showed increased fibrosis in comparison with both control and DIO *Nox2*-KO mice (Figure 5, E–H). However, there were no differences in fibrosis in the ventricular slices of all 4 mouse groups (Supplemental Figure 7A).

### Nox2 inhibition reduces ROS production in both DIO mice and PA-hiPSC-aCMs.

**Figure 6:**
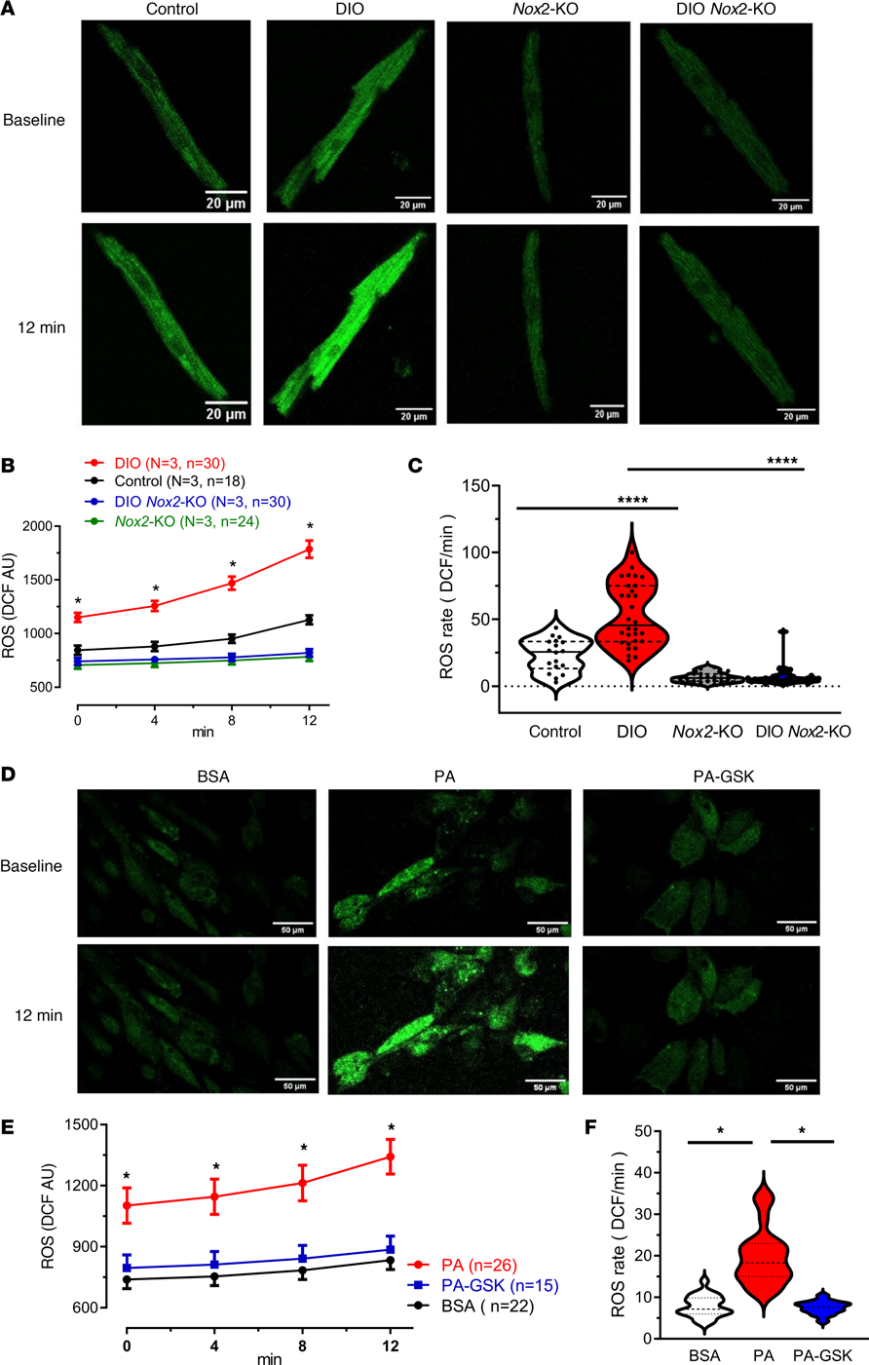
NOX2 inhibition in PA-treated hiPSC-aCMs using the NOX2 small-molecule inhibitor GSK-2795039 reverses obesity-induced ion channel remodeling. (**A**) Representative H2DCF staining of atrial cells from control, DIO, *Nox2*-KO, and DIO *Nox2*-KO mice. Scale bars: 20 μm. (**B**) Measured H2DCF fluorescence of atrial cells from control (*n* = 18 cells), DIO (*n* = 30 cells), *Nox2*-KO (*n* = 24 cells), and DIO *Nox2*-KO mice (*n* = 24 cells) at 0, 4, 8, and 12 minutes. (**C**) Rate of H2DCF increase in atrial cells from the 4 mouse groups. (**D**) Representative H2DCF staining of BSA-, PA-, and PA-GSK-hiPSC-aCMs. Scale bars: 50 μm. (**E**) Measured H2DCF fluorescence of BSA- (*n* = 22 cells), PA- (*n* = 26 cells), and PA-GSK-hiPSC-aCMs (*n* = 15 cells) at 0, 4, 8, and 12 minutes. (**F**) Rate of H2DCF increase in the 3 hiPSC-aCM groups. *P* > 0.05, *P < 0.05, ****P < 0.0001, by 2-tailed, unpaired Student’s *t* test.

Using H2DCFDA staining, a well-established technique for both visualizing and quantifying cytosolic ROS (34–36), we assessed ROS levels in control mice, DIO mice, *Nox2*-KO mice, DIO *Nox2*-KO atrial myocytes (pooled LA and RA cardiomyocytes), and BSA-, PA-, and PA-GSK-hiPSC-aCMs (Figure 6, A–F). Measurements were taken at baseline and after 12 minutes of staining. Substantial elevations in ROS levels were observed in DIO mice from baseline, distinct from control, *Nox2*-KO, and DIO *Nox2*-KO mouse atrial myocytes. This increase continued progressively over 12 minutes, a trend exclusive to the DIO group (Figure 6, B and C). Similarly, PA-treated hiPSC-aCMs showed increased ROS levels from the start with a marked rise noted over the 12-minute period, compared with the BSA- and PA-GSK-hiPSC-aCMs (Figure 6, E and F).

### Global proteomics and pathway enrichment analysis.

To identify the potential pathways involved in obesity-mediated AF, we performed proteomic profiling of pooled LA and RA protein lysates from control, DIO, and DIO *Nox2*-KO mice using a Q Exactive HF mass spectrometer coupled with an UltiMate 3000 RSLC nanosystem with a Nanospray Flex Ion Source. The total number of identified proteins across the 3 groups was 3,370 proteins, of which primarily cardiac-related genes were identified (Supplemental Figure 8, A–D). Contractile proteins such as Myl3, Ace, TnnI2, and TnnI3k were seen to be downregulated in DIO atria in comparison with both control and DIO *Nox2*-KO mouse atria (Supplemental Figure 8, A and B). In comparison, proteins involved in FA metabolism such as Cpt1α, Fabp4, and Acsl5 were upregulated in DIO mouse atria compared with control and DIO *Nox2*-KO mouse atria (Supplemental Figure 8, A and B). The number of differentially regulated proteins (log_2_ fold change ± 0.5) in the DIO versus control comparison was 41, of which 33 were upregulated and 8 downregulated (Supplemental Figure 8C). In the DIO *Nox2*-KO mice compared with DIO mice, 7 were upregulated and 48 downregulated (Supplemental Figure 8D).

To identify the signaling pathways that modulate Nox2*-*mediated atrial remodeling in DIO and DIO *Nox2*-KO mice, we used Kyoto Encyclopedia of Genes and Genomes (KEGG) pathway enrichment analysis. Major common downregulated pathways that were enriched in both the DIO versus control and DIO versus DIO *Nox2*-KO mice included the cardiac muscle contraction pathway (hsa04260), the dilated cardiomyopathy pathway (hsa05414), and the hypertrophic cardiomyopathy pathway (hsa05410) (Supplemental Figure 8, E and F). In contrast, pathways that regulate increased FA metabolism and FA digestion and absorption were the commonly implicated upregulated pathways (Supplemental Figure 8, G and H). In total, 22 pathways were commonly downregulated, and 6 pathways were upregulated (Supplemental Figure 8, I and J). We further validated 3 major pathways with Western blot: cardiac muscle contraction pathway (hsa04260), FA metabolism pathway (hsa0061), and oxidative phosphorylation pathway (hsa00190) (Supplemental Figure 8, E–H). This revealed that proteins modulating the cardiac muscle contraction pathway, such as cTnI, cTnT, Mybpc3, Mymo1, and Mlc2v, were ubiquitously decreased in DIO mice and their expression levels were restored in DIO *Nox2*-KO mice (Supplemental Figure 9, A–D and F). Only Myl7 decrease was not reversed after NOX2 inhibition (Supplemental Figure 9E). The increased expression of proteins involved in FA metabolism such as Cpt1a, Ppara, and Fabp3, observed in DIO mice, were abrogated in DIO *Nox2*-KO mice (Supplemental Figure 10, A–C).

### Transcriptomic and gene regulatory network analysis on human atrial tissue and hiPSC-aCMs.

**Figure 7:**
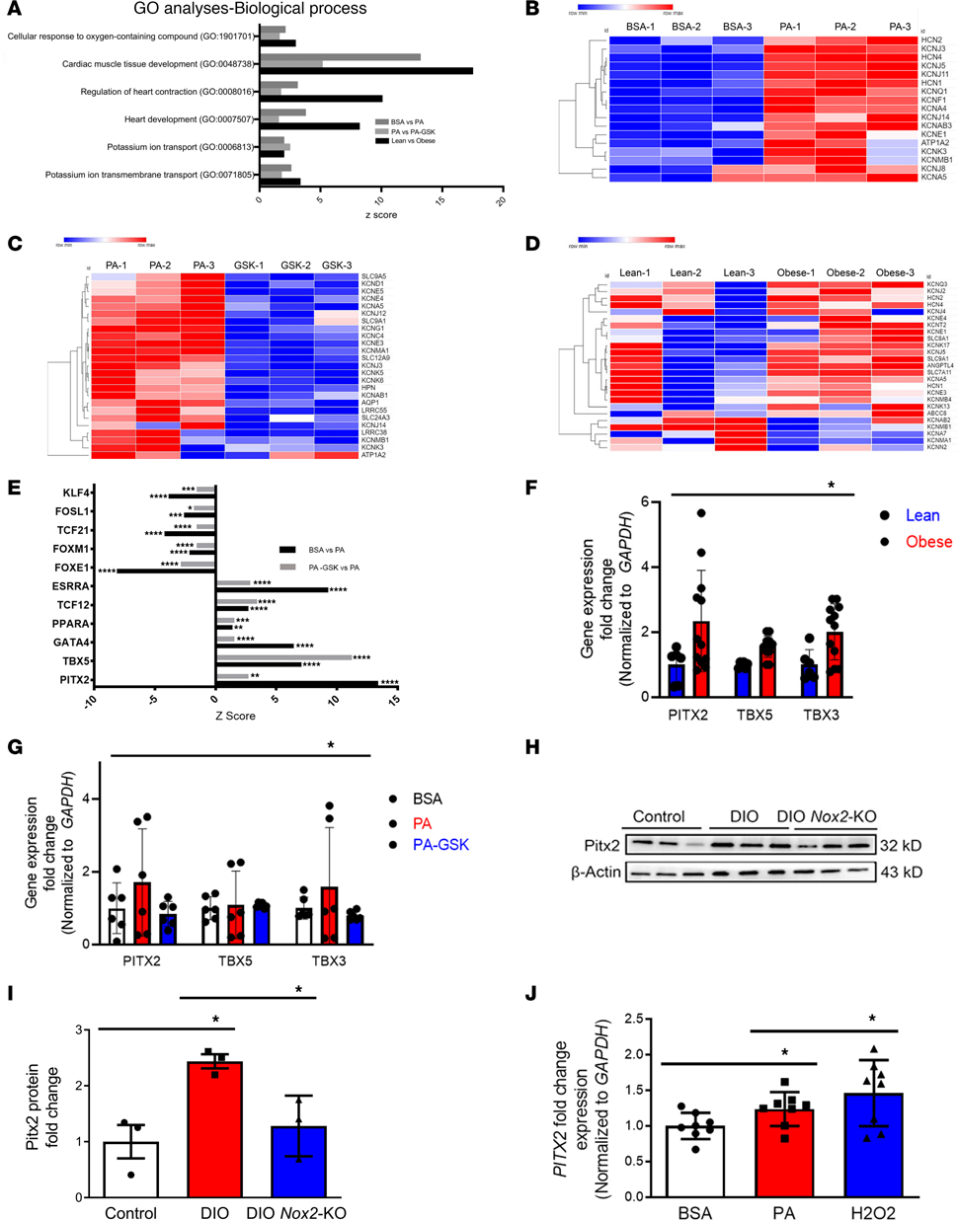
Transcriptomic and pathway enrichment analysis in BSA-, PA-, and PA-GSK-hiPSC-aCMs and lean and obese human atrial tissues. (**A**) Common biological process Gene Ontology (GO) pathways between the 3 comparisons. (**B**–**D**) Heatmaps of top upregulated and downregulated differentially expressed genes associated with the key GO pathway, potassium transmembrane transport heart contraction (GO0071805), in BSA- versus PA-hiPSC-aCMs (**B**), PA- versus PA-GSK-hiPSC-aCMs (**C**), and lean versus obese human atrial tissue (HAT) (**D**). (**E**) Common upregulated and downregulated cardiac-related transcription factors in hiPSC-aCMs. (**F** and **G**) qPCR validation of *PITX2*, *TBX5*, and *TBX3* genes in both hiPSC-aCMs (*n* = 3 each group) and HAT (*n* = 3 for lean, *n* = 6 for obese). (**H** and **I**) Pitx2 protein quantification using Western blotting in control, DIO, and DIO *Nox2*-KO mice. (**J**) *PITX2* qPCR quantification on BSA-, PA-, and H_2_O_2_-hiPSC-aCMs (*n* = 8 each group) (25 μM). *P < 0.05, **P < 0.01, ***P < 0.001, ****P < 0.0001, by 2-tailed, unpaired Student’s *t* test and 1-tailed ANOVA with Tukey’s multiple-comparison test.

Human atrial tissue (HAT) from obese individuals showed increased *NOX2* mRNA expression along with increased *KCNA5* and decreased *GJA5* mRNA expression compared with lean individual HAT (Figure 1, A and B, and Supplemental Figure 1, A and B). We then performed an unbiased global transcriptomic analysis by RNA sequencing (RNA-Seq) separately on lean versus obese HAT, control hiPSC-aCMs versus PA-hiPSC-aCMs, and PA-hiPSC-aCMs versus PA-GSK-hiPSC-aCMs to compare the differentially regulated gene expression pathways (Figure 7, A–D). Pathway enrichment analysis showed that the 3 comparisons shared 5 KEGG pathways (Supplemental Figure 11A), 21 Gene Ontology (GO) molecular function pathways (Supplemental Figure 11C), and 32 GO biological process pathways (Supplemental Figure 11E). Cardiac-related GO biological process pathways commonly enriched between the 3 comparisons were (a) cellular response to oxygen-containing compound (GO1901701), (b) cardiac muscle tissue development (GO0048738), (c) regulation of heart contraction (GO0008016), (d) potassium ion transport (GO0006813), and (e) potassium ion transmembrane transport (GO0071805) (Figure 7A). Potassium ion transmembrane transport (GO0071805), a pathway consisting of major potassium channels involved in AF, was specifically studied in all 3 comparisons (Figure 7, B–D). Common cardiac-related KEGG pathways between the 3 comparisons were (a) PPAR signaling pathway (hsa03320) (Supplemental Figure 11, I and J), (b) MAPK signaling pathway (hsa04010), (c) calcium signaling pathway (hsa04020) (Supplemental Figure 11, F–H), (d) hypertrophic cardiomyopathy pathway (hsa05410), and (e) dilated cardiomyopathy pathway (hsa05414) (Supplemental Figure 11B). Common cardiac GO molecular function pathways were (a) calcium ion binding (GO0005509), (b) potassium channel activity (GO005267), (c) voltage-gated potassium channel activity (GO005249), and (d) voltage-gated cation channel activity (GO022843) (Supplemental Figure 11D). Of the common KEGG and GO pathways, we selected potassium ion transport, potassium ion transmembrane transport, voltage-gated potassium channel activity, lipid localization (GO term GO0010876), and voltage-gated cation channel activity to perform upstream regulator analysis to identify potentially novel independent and integrated transcription factor (TF) networks and upstream TFs that might regulate the above key pathways (Figure 7E). Common TFs that were upregulated in all the RNA-Seq comparisons were *PPARA*, *PITX2*, *ESRRA*, *TBX5*, *GATA4*, and *TCF12*. Common TFs that were downregulated in all the RNA-Seq comparisons were *FOSL1*, *TCF21*, *FOXM1*, *FOXE1*, and *KLF4*. qPCR validation of both HATs and hiPSC-aCMs showed that *PITX2* and *TBX5* were increased in obese HATs and PA-hiPSC-aCMs (Figure 7, F and G). Similarly, DIO *Nox2*-KO mice showed reduced protein expression of *PITX2*, suggesting that NOX2 inhibition prevents obesity-induced atrial remodeling through *PITX2* (Figure 7, H and I). Lastly, to investigate whether NOX2 increase is associated with increased mRNA *PITX2* expression, we treated BSA-hiPSC-aCMs with 25 μM of hydrogen peroxide (H_2_O_2_) for 5 days. H_2_O_2_-hiPSC-aCMs showed a similar increase in *PITX2* mRNA expression compared with PA-hiPSC-aCMs, suggesting that PITX2 is indeed upregulated by ROS (Figure 7J).

**Figure 8:**
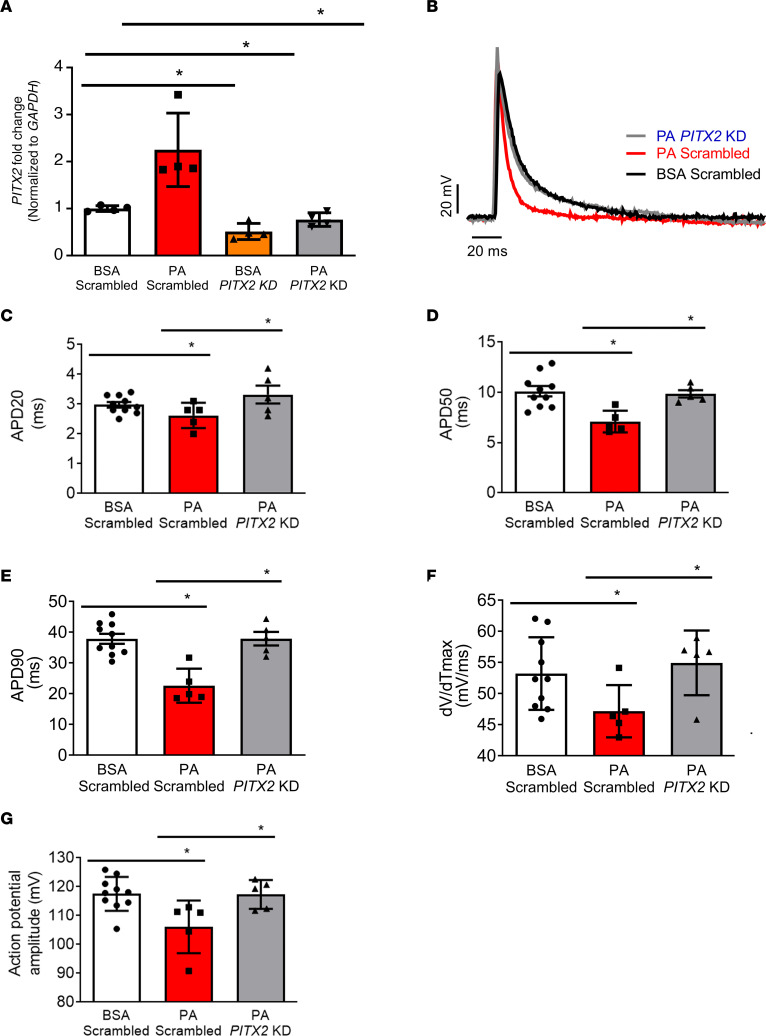
siRNA knockdown of PITX2 abrogates the effect of PA on hiPSC-aCMs. (**A**) *PITX2* expression in BSA-scrambled, PA-scrambled, BSA-*PITX2*-KD, and PA-*PITX2-*KD hiPSC-aCMs (*n* = 4 each). (**B**) Whole-cell patch clamping of BSA-scrambled (*n* = 10), PA-scrambled (*n* = 5), and PA-*PITX2-*KD hiPSC-aCMs (*n* = 5). (**C**) Measured APD at 20% repolarization (APD20). (**D**) Measured APD at 50% repolarization (APD50). (**E**) Measured APD at 90% repolarization (APD90). (**F**) Instantaneous rate of voltage change over time (dV/dT_max_), an indicator of atrial CV. (**G**) Maximum action potential amplitude (APA_max_). *P* > 0.05, *P < 0.05, by 2-tailed unpaired Student’s *t* test.

To directly assess the role of PITX2 in obesity-mediated ion channel remodeling, we performed small interfering RNA (siRNA) knockdown (KD) experiments in PA-hiPSC-aCMs (Figure 8). HiPSC-aCMs transfected with *PITX2*-specific siRNA and treated with both BSA and PA showed an approximately 40% decrease in *PITX2* expression compared with hiPSC-aCMs transfected with a scrambled sequence (Figure 8A). *PITX2* knockdown markedly abrogated PA-induced electrophysiological (EP) changes, including reversing the shortening of atrial APD20, APD50, and APD90 (Figure 8, B–E); the decrease in maximum upstroke velocity (Figure 8F); and the decrease in maximum AP amplitude (Figure 8G), compared with PA-scrambled-hiPSC-aCMs. Our results provide strong evidence that increase in PITX2 directly modulates EP changes in obesity-mediated AF.

## Discussion

Oxidative stress plays a key role in mediating obesity-induced AF by the activation of NOX2, a major non-mitochondrial source of ROS production (11–13). Antioxidants, however, have not shown any benefit for treating AF, in part because of their failure to target the specific pathways of ROS production (20, 21). Here, using both *Nox2*-KO mice and mature hiPSC-aCMs, we showed that NOX2 mediates increased oxidative stress and ROS production in obesity-mediated AF. Treatment of DIO and DIO *Nox2*-KO mice with a generic NOX blocker, apocynin, and of PA-treated hiPSC-aCMs with a NOX2-specific inhibitor, GSK2795039, abrogated obesity-mediated ion channel remodeling and atrial fibrosis by reducing oxidative stress and ROS production. Unbiased transcriptomics and gene regulatory network analysis revealed that NOX2 mediates atrial ion channel and structural remodeling in obesity-mediated AF in DIO mice, PA-treated hiPSC-aCMs, and HAT from obese individuals by the upregulation of PITX2. Treatment of hiPSC-aCMs with hydrogen peroxide (H_2_O_2_), a known by-product of NOX2, increased *PITX2* mRNA expression, suggesting ROS-mediated upregulation of PITX2 in obesity-mediated AF. Collectively, our findings show that genetic and pharmacological inhibition of NOX2 abrogates ion channel and structural remodeling in both *Nox2*-KO mice and mature hiPSC-aCMs and prevents the development of obesity-mediated AF by modulating PITX2 expression.

Oxidative stress and ROS production mediate myocardial remodeling through the creation of an electrophysiological (EP) substrate for arrhythmogenesis (18, 19). The assessment of atrial EP of DIO-apocynin mice, DIO *Nox2*-KO mice, and PA-GSK-hiPSC-aCMs showed prolongation of atrial APD, normalization of remodeled ion channels, a decrease in atrial fibrosis, and an increase in atrial CV in comparison with the DIO mice. We previously reported that DIO mice are more prone to AF by upregulating the K_v_1.5 and I_Kur_ along with downregulation of Na_v_1.5 and I_Na_, causing shortening of the atrial APD (9). In humans, loss-of-function *SCN5A* mutations, encoding Na_v_1.5, not only decrease cardiac conduction and shorten atrial APD, but also increase susceptibility to AF (37, 38). Thus, improvements in both atrial APD and CV and restoration of I_Na_ in DIO *Nox2*-KO mice support reduced AF vulnerability in these mice. Studies have reported that both cytosolic and mitochondrial ROS downregulate Na_v_1.5 via PKC-dependent phosphorylation of the channel (12, 31, 32). Secondly, chronic treatment with angiotensin II, a mediator of NOX2 increase, has been seen to directly significantly reduce I_Na_ at 10 days and 21 days (39). Thus, our data that showed failure to increase PKC-α and PKC-δ expression in DIO *Nox2*-KO mice as compared with DIO mice provide a plausible explanation for the modulation of I_Na_.

We previously reported that DIO mice are more susceptible to AF because of ion channel remodeling, which causes shortening of the atrial APD (9). Here, we showed that cardiac muscle contraction, potassium transmembrane transport, and PPAR signaling were enriched in the proteomics analysis of control, DIO, and DIO *Nox2*-KO mice and the RNA-Seq analysis of BSA-, PA-, and PA-GSK-hiPSC-aCMs and obese HAT. Upstream regulator analysis identified *PITX2* as a major upregulated gene in obese HATs and PA-treated hiPSC-aCMs compared with lean HATs and BSA- and PA-GSK-hiPSC-aCMs, respectively. While there has been extensive research on the impact of loss-of-function *PITX2* in atrial arrhythmogenesis (26, 27), the role of increased *PITX2* in atrial remodeling is unclear. First, measuring *PITX2* expression in human atrial myocytes from patients in sinus rhythm and AF, Pérez-Hernández et al. found that increased I_Ks_ and reduced I_Ca,L_ were mediated by increased *PITX2* expression, implicating its role in electrical remodeling during AF (40, 41). Second, treating BSA-hiPSC-aCMs with H_2_O_2_, which has been shown to increase NOX2-mediated ROS production (42), increased *PITX2* as seen with PA treatment. Importantly, studies have shown that H_2_O_2_ incubation in ventricular myocytes leads to decreased cardiac I_Na_ density (13). In addition, PITX2 knockdown using siRNA abrogated the EP effects of PA treatment in PA-hiPSC-aCMs compared with PA-treated scrambled-hiPSC-aCMs. Collectively, our findings suggest that increased NOX2 is associated with *PITX2* upregulation, which mediates ion channel remodeling in obesity-mediated AF. Nonetheless, additional studies will be necessary to fully elucidate this link in both in vivo and in vitro models.

A major determinant of altered atrial APD is potassium channel activity (43). Mutations in *KCNQ1*, *KCNA5*, and *KCNH2* encoding I_Ks_, I_Kur_, and I_Kr_, respectively, have been implicated in the pathogenesis of early-onset AF (43–45). DIO *Nox2*-KO mice showed a marked reduction in total I_K_ and I_Ks_, and reduced mRNA expression of *KCNQ1* and *KCNE1*, which encode I_Ks_. The normalization of I_Ks_ in DIO *Nox2*-KO mice and PA-GSK-hiPSC-aCMs as compared with DIO mice and PA-treated hiPSC-aCMs, respectively, is in part explained by the oxidation of potassium channels, especially K_v_7.1 (39). We and others have shown that gain-of-function mutations in *NPPA*, encoding ANP, have also been associated with increased I_Ks_ (25, 46, 47). We recently reported that increased I_Ks_ expression and function were accompanied by atrial AP shortening in hiPSC-aCMs expressing an *NPPA* mutation (23). Secondly, ANP expression intrinsically mediates electrical remodeling and cardiac electrophysiology through cAMP signaling as evidenced by previous studies (46, 47). ANP and NOX generation have also been linked through a feed-forward cycle via the NOX/Src axis, promoting excess production of both (48). Importantly, *NPPA* mRNA expression is increased in DIO mice but is unchanged in DIO *Nox2*-KO mice. Thus, we postulate that enhanced I_Ks_ is due to increased NOX2 expression directly modulating increased ANP secretion in DIO mice.

We applied an electro-metabolic maturation approach to generate mature hiPSC-aCMs that are the best available surrogate for atrial tissue (22–25). Our findings demonstrate that blocking NOX2 effectively prevents obesity-mediated ion channel remodeling and atrial fibrosis by reducing oxidative stress and ROS production in both DIO mice and PA-treated hiPSC-aCMs. Furthermore, to model obesity in vitro and recapitulate increased oxidative stress and lipid overload, mature hiPSC-aCMs were chronically treated with PA, the most common FA in the human diet, to increase serum-free FA circulation in the heart (49–51). Studies on PA-treated murine cardiomyocytes and HL-1 cells showed that PA alone selectively increases NOX2 expression, mitochondrial abnormalities, aberrant calcium transients, and arrhythmia as compared with OA (14). Our findings also revealed a distinct response to OA treatment, characterized by a prolongation of atrial AP with increase in the APD50 and the maximum upstroke velocity in contrast to PA treatment. Importantly, the differential effects observed between OA and PA treatments suggest that the abnormalities in obesity-related cardiac dysfunction are mostly driven by saturated fats like PA.

We used a global *Nox2*-KO mouse model to examine the role of NOX2 in obesity-mediated AF. As NOX2 is expressed not only in cardiomyocytes and endothelial cells but also in fibroblasts and inflammatory cells, it is possible that other cell types may contribute to the adverse atrial remodeling (52). Studies have also shown previously that an increase in ROS in DIO hearts is primarily generated by an increase in NOX2 rather than mitochondria-specific NOX4 (53–55). Interestingly, a recent report showed that superoxides can directly activate mitochondrial K_ATP_ channels, which may explain the effectiveness of generic mitochondrial ROS scavengers such as MitoTEMPO in reversing the atrial phenotype in obese mice (56, 57). Furthermore, H2DCFDA staining in both rescue groups, DIO *Nox2*-KO mice, and PA-GSK-hiPSC-aCMs showed reduced ROS in comparison with the obese groups, DIO mice, and PA-hiPSC-aCMs, which suggests that ROS production in the atria of DIO mice and PA-hiPSC-aCMs is driven by increased NOX2 expression.

NOX2-derived ROS and changes in intracellular redox state lead to aberrant calcium release and arrhythmias by modulating excitation-contraction coupling through oxidative modifications of ryanodine receptor 2 (RyR2), CamKII, phospholamban, and sarco/endoplasmic reticulum calcium-ATPase (SERCA2a) (58, 59). For example, oxidative activation of CaMKII through ROS signaling has been shown to be pro-arrhythmic in diabetic mice and is linked to the pathogenesis of several cardiac diseases, including AF (60, 61). Our proteomics analysis suggests that cardiac muscle contraction was markedly reduced in DIO mice but restored in DIO *Nox2*-KO mice. DIO mice also exhibited reduced I_Ca,L_ and atrial contractility, which was reversed in DIO *Nox2*-KO mice. Given the important relationship between NOX2 and redox-mediated changes to excitation-contraction, a combined strategy targeting both calcium handling proteins and NOX2 should be a focus for future studies. Our data suggest that the reduction in the Cai amplitude is more reflective of the reduction in the I_Ca,L_ (Figure 2, G and H) that decreases the excitation-contraction coupling gain and not due to changes in RyR2 that could affect the sarcoplasmic reticulum calcium release. We also showed that NOX2 protein inhibition prevents obesity-induced LA enlargement in DIO *Nox2*-KO mice. As NOX2 has been implicated in cardiac hypertrophy (17–21), our data suggest that NOX2 inhibition may be a viable therapeutic approach for atrial hypertrophy.

Our studies emphasize the key role played by NOX2-generated ROS and PITX2 in obesity-mediated AF. Our mechanistic studies demonstrate that targeting atrial oxidative injury and ROS production with both a NOX blocker and a NOX2-specific inhibitor and genetic ablation of NOX2 in obese mice and PA-treated hiPSC-aCMs may not only prevent and treat AF but also slow its progression in obese individuals. Our findings have important implications for targeted therapy for AF patients with obesity. As the response to current antiarrhythmic drugs in an individual patient is highly variable and membrane-active drugs can be associated with significant toxicity, targeted inhibition of NOX2 may be a novel therapeutic approach for obese individuals at risk for AF and as adjunctive therapy for patients with obesity-mediated AF.

### Limitations.

First, we used both LA and RA samples for whole-cell patch clamping, EP, and molecular analysis. However, it is essential to acknowledge that the RA and LA are known to exhibit significant differences in gene expression and function. These intrinsic disparities could impact our findings and should be carefully considered in interpreting the results. This limitation underscores the importance of analyzing both atria separately to fully understand the distinct electrophysiological and molecular characteristics of each chamber in the context of our study. Second, the use of mature hiPSC-aCMs treated with FAs as an experimental model, though valuable, represents a simplified approach and thus cannot recapitulate the in vivo complexity of obesity, which consists of many lipids. Third, it has been established that current protocols do not achieve fully differentiated atrial cardiomyocytes and result in some heterogeneity. Our protocol typically yields about 80%–90% pure iPSC-aCMs and less than 6% fibroblasts based on immunostaining analysis as we have previously described and show in Supplemental Figure 11 (23–25). Fourth, GAPDH and β-actin were used as housekeeping genes for qPCR and Western blotting analyses. Reports indicate that the expression levels of GAPDH and β-actin can be affected in the left ventricle and RA of hearts from aged and diabetic models and that these alterations are chamber specific (62, 63). Despite this, some findings suggest that GAPDH possesses high expression stability in heart failure and normal heart conditions (62). Nonetheless, a broader range of housekeeping genes may further strengthen our findings.

## Methods

### Sex as a biological variable.

For the human studies involving collection of human atrial tissue, samples were obtained from both males and females. Sex as a biological variable was not considered because of insufficient statistical power to analyze sex-stratified effects. For the mouse studies, only male mice were used because female mice do not reach the 33 g obesity threshold after 10 weeks on an HFD owing to their resistance to obesogenic effects (9). Control C57BL/6J and *Nox2*-KO male mice were fed a 60% HFD from Teklad (TD06414) for 10 weeks (9). Further research is needed to determine whether our findings in male mice also apply to female mice and whether there are sex-specific differences in obesity-mediated AF.

### Human atrial tissue.

We obtained written informed consent to collect adult human atrial tissue during cardiothoracic surgery under a University of Illinois–Chicago Institutional Review Board–approved protocol. The atrial tissue was obtained, stored, and processed as we previously described (23). Briefly summarizing, we obtained atrial tissue from the RA and LA at the time of surgery during venous cannulation, after which it was transported to the laboratory in warmed EDTA, as previously described (64). Half the tissue was used for atrial cardiomyocyte isolation using a Langendorff-free isolation protocol (64), and the other half was immediately snap-frozen using liquid nitrogen for further use for RNA and protein isolation (23). For RNA extraction, 0.5 mL of TRIzol was added to the sectioned tissue, which was stored at –80°C.

### Generation of mouse models.

Animal studies were conducted following protocols approved by the University of Illinois–Chicago’s institutional animal care and use committee (Animal Care Committee [ACC]) and adhering to NIH guidelines. The study used *Nox2*-KO mice (gp91phox^–/–^ or Cybb^–/–^) with a neomycin resistance gene inserted at exon3 of gp91phox, resulting in the absence of NOX2 heterodimer complexes and subsequent lack of superoxide production (65). Breeding experiments involved male hemizygous mice crossed with control C57BL/6J mice and *Nox2*-KO mice.

### DIO mice generation.

Control C57BL/6J and *Nox2*-KO mice were fed a 60% HFD from Teklad (TD06414) for 10 weeks as we previously described (9). We previously reported that female mice do not reach the 33 g threshold for obesity after 10 weeks. Thus, only male mice participated in the study.

### Human iPSC culture and hiPSC-aCM differentiation.

HiPSC-aCMs were generated from reprogrammed peripheral blood mononuclear cells (PBMCs) from patient 1, with no prior diagnosis of AF, recruited to the Human Cardiac Atrial Tissue Biorepository as previously described (23). HiPSCs were seeded at 500,000 cells per well on vitronectin-coated plates and cultured in mTesR medium until 80%–90% confluent. Differentiation was initiated using the Cardiomyocyte Differentiation Kit (Gibco) and guided toward the atrial subtype using all-*trans* retinoic acid (22–25). The cellular population was purified through glucose starvation and lactate replacement, resulting in contracting monolayers. Our protocol typically yields approximately 80%–90% pure iPSC-aCMs and less than 6% fibroblasts based on immunostaining analysis as we have previously described (23–25) and show in Supplemental Figure 11. iPSC-aCMs were then matured after dissociation and replating on fibronectin-coated plates and maintained in cardiomyocyte maintenance medium supplemented with T3, insulin-like growth factor-1, dexamethasone, and BSA-bound PA/OA as previously described (22–25).

### Fatty acid treatment and Nox2 inhibition in hiPSC-aCMs.

To prepare PA and OA solutions, approximately 1 mL of 250 mM fatty acid (FA) solutions were made in 100% molecular-grade ethanol. PA, being a powder, and OA, being liquid at room temperature, were measured out into Eppendorf tubes under sterile conditions. The saturated FAs in ethanol were then placed in a 70°C water bath until they dissolved, and the solution became clear, a step made necessary by their insolubility at room temperature. For a 7:1 FA mixture of 25 mL, 1.061 mL of the solution was used, adding 265.3 μL of each FA to achieve the target FA stock concentration of 10.5 μM. The stock concentration was in turn dissolved in cardiomyocyte maintenance medium to get a final FA concentration of 500 μM. Mature hiPSC-aCMs were exposed to either BSA or BSA-conjugated PA or OA at a concentration of 500 μM each for 5 days to mimic FA exposure observed in obesity. In the case of PA-GSK-hiPSC-aCMs, GSK-2795039 was resuspended in DMSO and dissolved in PA-conjugated medium to achieve a final concentration of 20 μM. Similarly to PA-hiPSC-aCMs, PA-GSK-hiPSC-aCMs were treated with this medium for 5 days.

### Cellular electrophysiology, calcium transient recordings, and electrical mapping studies.

Left and right atrial cardiomyocytes from mice were isolated in a Langendorff perfusion system, as previously described. Whole-cell patch clamping on both mouse cardiomyocytes and hiPSC-aCMs for APD, I_Na_, I_Ca,L_, and I_Ks_ recordings was performed according to previously published protocols (9, 62, 63). Optical voltage mapping recordings were performed on the IonOptix MyoPacer system using the FluoVolt Membrane Potential Kit (Thermo Fisher Scientific). HiPSC-aCMs attached to confocal dishes were incubated with Tyrode’s solution (140 mM NaCl, 4.56 mM KCl, 0.73 mM MgCl_2_, 10 mM HEPES, 5.0 mM dextrose, 1.25 mM CaCl_2_) containing 1× FluoVolt (Sigma-Aldrich) for 15–20 minutes. Cells were then washed with normal Tyrode’s solution before being viewed on the eGFP setting for APD recordings (23).

For calcium transients, isolated LA and RA cardiomyocytes were plated on laminin-coated coverslips and observed under an inverted Nikon TE300 microscope. The cells were incubated with 2 μM Fura-2 AM for 15 minutes, followed by a washout period of 20 minutes for dye de-esterification. Excitation was performed at 340 and 380 nm, and emission was detected at 510 nm. Transillumination with red light (>650 nm) was used to avoid interference with Fura-2 epifluorescence. Contractility was measured by tracing of sarcomere length. Field stimulation was applied at 1 Hz until a steady state was reached, and contractility and [Ca^2+^]_i_ transients (amplitude and kinetics) were analyzed using IonOptix software (63, 64).

Isolated Langendorff-perfused hearts underwent LA and RA and ventricular epicardial activation mapping (66). Using previously published protocols that used a Mapping Labs electrical mapping system, a 2-dimensional multielectrode array containing 64 electrodes in an 8 × 8 grid was used to study activation times and atrial and ventricular conduction velocities computed at a cycle time of 100 milliseconds (67). The propagation of the beats across the LA, RA, and left ventricle over 5 seconds was analyzed. Activation maps were generated for each beat, depicting sequential activation from one localized region to the entire matrix area. CV was determined by measurement of the time required for propagation from the point of minimum to maximum activation. The averaged CV was computed and binned, with each bin containing enough vectors that had similar directions. This process was repeated across beats from different recordings for a specific sample, and the overall average CV for each sample was calculated from these measurements (67).

### Atrial fibrosis measurements.

Preparation of paraffin sections and subsequent staining were done using the services of the Research Histology Core at the University of Illinois–Chicago. For atrial fibrosis analysis, we followed previously published protocols (9). We harvested mouse hearts, fixed them in 10% neutral formalin overnight, embedded them in paraffin, and cut 5-μm-thick sections using the Epredia HM 340E Electronic Rotary Microtome at the histology core. These sections were stained with Masson’s trichrome and Picrosirius red stains (MilliporeSigma) after deparaffinization. The cardiac fibrosis ratio was analyzed and calculated using ImageJ (NIH) by division of the total cardiomyocyte area in the atrium.

### qPCR analyses.

Total RNA was isolated from human atrial tissue, mouse LA and RA, and hiPSC-aCMs using TRIzol reagent (Invitrogen), following the manufacturer’s instructions to ensure the extraction of high-quality RNA. The concentration and purity of the isolated RNA were meticulously assessed using a NanoDrop 2000 spectrophotometer (Thermo Fisher Scientific), with 1 μg of total RNA used for each reverse transcription reaction. Reverse transcription to synthesize cDNA was conducted using SuperScript III Reverse Transcriptase (Thermo Fisher Scientific), adhering to the manufacturer’s protocol to optimize the fidelity and efficiency of the cDNA synthesis.

For the qPCR analysis, specific assays and primers were selected for target genes (detailed in Supplemental Table 2) with glyceraldehyde 3-phosphate dehydrogenase (GAPDH) serving as the normalization reference gene. qPCR reactions were performed on an ABI QuantStudio 5 system (Applied Biosystems), using SYBR Green PCR Master Mix to accurately detect and quantify PCR amplification products. The thermal cycling conditions were carefully optimized specific to each target assay, comprising an initial denaturation step followed by 40 cycles of denaturation, annealing, and extension. Relative expression levels of the target genes were calculated using the ΔΔCt method, by the quantification of gene expression changes in the experimental samples relative to control. For the ΔCt​ calculation, the cycling time (Ct) value of the target gene was subtracted from the Ct value of GAPDH in the same sample using ΔCt = Ct_target gene_ − Ct_reference_ _gene_. The ΔΔCt value was then calculated using ΔΔCt = ΔCt_experimental_ *–* ΔCT_control_. The relative expression for the gene was in turn calculated using relative gene expression = 2^−ΔΔCt^.

### Protein isolation and Western blots.

Proteins from both mouse hearts and hiPSC-aCMs were isolated based on previously published protocols using 1× RIPA buffer (9, 23). Each sample containing 50 μg of protein was subjected to SDS-PAGE gel electrophoresis. The resolved gels were then electrotransferred onto 0.2 μm PVDF membranes. After a 2-hour blocking step with 5% BSA, membranes were probed with specific antibodies for target proteins (Supplemental Table 3). Blots were developed using either anti-rabbit HRP or anti-mouse HRP and scanned with C280 imaging systems (Azure Biosystems). ImageJ software was used to determine protein signal densities, which were subsequently normalized to corresponding β-actin signal densities.

### Transthoracic echocardiography.

Echocardiography measurements were conducted in unconscious mice using an induction chamber with 3% isoflurane. Isoflurane was adjusted to 0.5%–1.5% to maintain a target heart rate of 450 ± 50 bpm. Ultrasound scans were obtained with the Vevo 2100 imaging system and MS550D probe (FUJIFILM VisualSonics Inc.) at a center frequency of 40 MHz. M-mode tracings, mitral inflow, and tissue velocities were measured using pulsed-wave Doppler and tissue Doppler modes. Measurements were taken from at least 3 consistent cardiac cycles within the target heart rate range. Mice were monitored for recovery after echocardiography.

### RNA-Seq and proteomics analyses.

RNA quality and quantity were evaluated using the Agilent Bioanalyzer (25). RNA-Seq was conducted following the TruSEQ mRNA-Seq library protocol and performed on the Illumina NovaSEQ6000 platform, as previously described (25). For analysis of RNA-Seq raw FASTQ files, the BioJupies online RNA-Seq platform (https://maayanlab.cloud/biojupies) was used for pathway and upstream transcription factor analyses. The Enrichr option available on the same platform (https://maayanlab.cloud/Enrichr/) was used for upstream regulator analysis. The Enrichr-KG database contains gene set libraries, including GO pathways, KEGG analysis, REACTOME pathway analysis, and TRRUST transcription factor analysis, among others.

The mass spectrometry analysis was performed by the Mass Spectrometry Core in the Research Resources Center of the University of Illinois–Chicago. For the analyses, 9 samples (*n* = 3 for control, DIO, and DIO *Nox2*-KO) at 100 μg each were subjected to tryptic digestion using the S-Trap Micro kit (ProtiFi). The digested proteins were labeled with a TMT10plex Isobaric Label Reagent Set (Thermo Fisher Scientific), combined, and desalted using an Oasis PRiME HLB 96-well plate (Waters). The pooled TMT-labeled peptides were fractionated into 80 fractions using offline high-pH reverse-phase (HPRP) liquid chromatography with an XBridge BEH C18 Column, 130 Å, 3.5 μm, 4.6 mm × 250 mm (Waters). Every 13th fraction was concatenated together, resulting in 12 concatenated fractions that were dried and resuspended for LC-MS analysis. Approximately 1 μg of concatenated HPRP fractions were analyzed using a Q Exactive HF mass spectrometer coupled with an UltiMate 3000 RSLC nanosystem and a Nanospray Flex Ion Source (Thermo Fisher Scientific). Digested peptides were separated on a Waters BEH C18 column at a flow rate of 300 nL/min using a gradient of 0.05% trifluoroacetic acid in H_2_O, solvent A, and 0.05% TFA in acetonitrile, solvent B. Full MS scans (resolution 120,000) were acquired over an *m*/*z* range of 350–1,400, and the 15 most intense peaks were fragmented for tandem mass spectra (resolution 60,000). Ion selection thresholds and maximum allowed ion injection times were set for both full scans and fragment ion scans. Spectra were searched against the UniProt mouse database using Mascot Daemon (2.6.0; https://www.matrixscience.com/server.html; Matrix Science) with specified parameters. The search results were analyzed using Scaffold Q+S software (https://www.proteomesoftware.com/products/scaffold-qs, current version 5.3.3) for compilation and normalization of spectral counts. TMT purity correction was applied. Protein identification filtering criteria included a 1% false discovery rate and minimum peptide count. The acquired differentially expressed genes file was further analyzed using the BioJupies online platform to look at pathway enrichment analyses and upstream regulators.

### H2DCFDA staining of mouse atria cells and hiPSC-aCMs and ROS measurements.

As previously described, we measured ROS levels using a well-established technique (34–36, 66, 68). Briefly summarizing, freshly isolated mouse LA and RA cells and hiPSC-aCMs in Tyrode’s solution (in mM: NaCl 140, KCl 5.4, CaCl_2_ 1, MgCl_2_ 1, glucose 5.5, HEPES 10, pH 7.4) were incubated for 30 minutes at 37°C with 10 μM 29,79-dichlorofluoresceindiacetate (DCFH-DA). DCFH-DA, being nonpolar, readily diffuses into cells, where it is hydrolyzed to the nonfluorescent polar derivative DCFH, thereby trapped within the cells. In the presence of ROS, DCFH is oxidized to the highly fluorescent 29,79-dichlorofluorescein (DCF). Extracellular DCFH-DA was washed out, and DCF fluorescence measurements were taken at room temperature with a Zeiss LSM 710 confocal microscope. Cells were excited with low laser power at 480 nm, and the emitted fluorescence was recorded at 610 nm with 400-millisecond scans every 3 minutes (68). The rate of ROS production during 12 minutes was obtained from the fitting of a linear regression to the DCF/min slope (GraphPad Prism).

### siRNA knockdown experiments.

Mature hiPSC-aCMs were treated with either scrambled or PITX2-specific siRNAs (#SR321325B, 10 pmol) using Lipofectamine RNAiMAX (Invitrogen) (69). Stock solutions of both Lipofectamine and siRNA, prepared at 10 μM, were initially diluted in Opti-MEM medium (Gibco, Life Technologies). These solutions were then mixed at a 1:1 ratio and allowed to incubate for 5 minutes to form the siRNA-lipid complex. This complex was subsequently added to the cells dropwise, and the cells were incubated for 2 days, after which the medium was replaced (69). Following a recovery period of 2 days, the transfected cells were treated with either BSA or PA for a period of 5 days.

### Statistics.

Data are presented as mean ± SD unless otherwise specified. Significance is denoted as *P < 0.05, **P < 0.01, ***P < 0.001, ****P < 0.0001, with *P* < 0.05 considered significant. Statistical analyses included nonparametric unpaired and 2-tailed Mann-Whitney *U* test for data with normal distribution, and either 1-way or 1-tailed ANOVA with post hoc Bonferroni’s corrections for multiple groups. Skewed data are expressed as medians with the first and third quartiles. Continuous variables were evaluated using unpaired 2-tailed Student’s *t* test or ANOVA, while categorical data were compared using Fisher’s exact test (9, 25).

### Study approval.

To collect human atrial tissue from different cardiac surgery patients, we used the University of Illinois–Chicago Institutional Review Board–approved protocol to enroll participants after receipt of informed written consent. Mouse studies were conducted according to previously approved Animal Care Committee (ACC) protocols approved by the Office of Animal Care and Institutional Biosafety, University of Illinois–Chicago.

### Data availability.

Data are available in public repositories, in the Supporting Data Values file, or from the corresponding author upon request. The RNA-Seq and proteomics data reported in this article were deposited into the NCBI’s Gene Expression Omnibus (GEO) database with the accession number GSE271748.

## Author contributions

AS, JD, HC, MAP, and DD designed the experiments. AS was responsible for generating and maintaining different mouse strains, conducting iPSC culture and iPSC-aCM differentiation, applying fatty acid treatment, and performing RNA isolation and sample preparation for RNA-Seq, real-time PCR, Western blots, optical voltage mapping, and data analysis. AS also wrote the manuscript and performed and interpreted the RNA-Seq analysis using BioJupies software. JD conducted EP whole-cell current-clamp recordings and calcium transient measurements on both mouse aCMs and iPSC-aCMs and, together with AS, performed ROS measurements on these cells. MAP assisted with EP whole-cell voltage-clamp recordings and analyzed the data. HC, along with AO and MB, assisted with iPSC line generation, aCM differentiation, and optical voltage mapping measurements. HC also performed flow cytometry on differentiated iPSC-aCMs. OL assisted in recruiting cardiac surgery patients for atrial tissue and conducted real-time PCR on iPSC-aCMs. JJ performed mouse echocardiography across all mouse groups. SBN analyzed proteomics data in atrial lysates of different mouse groups. KA, MM, and LER provided HAT and whole blood for PBMC extraction. DD supervised the experiments and provided funding support, in addition to offering critical revisions of the manuscript. SGO and JR also contributed critical revisions to the manuscript. All authors provided critical feedback and contributed to the final manuscript.

## Supplementary Material

Supplemental data

Unedited blot and gel images

Supporting data values

## Version 1 08/15/2024

In-Press Preview

## Version 2 09/17/2024

Electronic publication
