# Supplementary material for: Modulation of NOX2 causes obesity-mediated atrial fibrillation
Source: J Clin Invest. 2024 Aug 15;134(18):e175447. doi: 10.1172/JCI175447 (PMC11405042; doi:10.1172/JCI175447)
Supplement: Supplemental data [file jci-134-175447-s126.pdf]

Table S1. Clinical characteristics of patients undergoing cardiac surgery from whom atrial tissue was obtained.

	**Lean Weight (n = 6)**	**Overweight (n = 6)**	**Obesity (n = 12)**
Age (years)	53.67 ± 20.20	65.67 ± 15.23	54.33 ± 13.69
Body Mass Index (kg/m^2)	22.43 ± 1.33	27.22 ± 0.65*	33.40 ± 3.93*†
Ejection Fraction (%)	60 ± 7.75	55.33 ± 16.57	51.02 ± 12.16
Left Atrial Size (cm)	3.56 ± 0.56	3.73 ± 0.46	4.28 ± 1.04
Gender: Male	5 (83.3%)	5 (80%)	9 (70%)
Female	1 (16.7%)	1 (20%)	3 (30%)
Ethnicity/Race: African American	2 (33.3%)	1 (16.7%)	6 (50%)
Asian	1 (16.7%)	0 (0%)	1 (8.3%)
Hispanic	3 (50%)	3 (50%)	4 (33.3%)
White	0 (0%)	2 (33.3%)	1 (8.3%)
Cardiac Procedure: CABG	4 (66.7%)	3 (60%)	7 (58.3%)
Valve replacement	2 (33.3%)	2 (33.3%)	4 (33.3%)
Other	0 (0%)	1 (16.7%)	1 (8.3%)
Diabetes Mellitus	1 (16.7%)	3 (60%)	5 (50%)
Hypertension	6 (100%)	5 (83.3%)	10 (100%)
Coronary Artery Disease	4 (66.7%)	4 (66.7%)	8 (66.67%)
History of Myocardial Infarction	1 (16.7%)	0 (0%)	3 (25%)
Congestive Heart Failure	0 (0%)	2 (33.3%)	6 (50%)
Valvular Heart Disease	2 (33.3%)	3 (50%)	6 (50%)

Stroke or Transient Ischemic Attack	0 (0%)	0 (0%)	2 (16.67%)

*p-value <0.05 when compared to the “Lean Weight” group. *† p value <0.05 when compared to the “Overweight” group

Table S2

	**Control (n=7)**	**DIO (n=5)**	***Nox2*-KO (n=5)**	**DIO *Nox2*-KO (n=5)**	$P_{Control\ vs.\ DIO}$	$P_{DIO\ vs.\ DIO\ Nox2\text{-}KO}$	P_{ANOVA}	***F***
**BW (g)**	27.5 ± 2.9	44.8 ± 3.9	24.5 ± 0.9	40.9 ± 3.7	**<0.0001**	0.15	**<0.0001**	43.1
**EF**	61.8 ± 7.1	59.4 ± 6.3	59.4 ± 6.3	60.3 ± 9.6	0.57	0.83	0.94	0.12
**FS (%)**	31.9 ± 4.5	31.3 ± 4.3	30.9 ± 7.2	31.8 ± 2.3	0.56	0.82	0.97	0.03
**A'/E'**	0.9 ± 0.2	1.1 ± 0.3	1.0 ± 0.1	1.1 ± 0.1	0.28	0.52	0.27	1.44
**MV E/A**	1.3 ± 0.2	1.3 ± 0.2	1.4 ± 0.2	1.3 ± 0.1	0.6	0.72	0.94	0.05
**CO (mL/min)**	20.3 ± 3.2	18.8 ± 4.0	17.4 ± 2.4	22.9 ± 3.9	0.94	0.5	0.24	1.6
**LVPWs (mm)**	1.1 ± 0.19	1.1 ± 0.14	1.1 ± 0.14	0.9 ± 0.60	0.94	0.52	0.68	0.4
**LVPWd (mm)**	0.7 ± 0.10	0.8 ± 0.1	0.7 ± 0.1	0.8 ± 0.1	0.16	0.83	0.28	1.4
**LA (mm)**	1.7 ± 0.20	2.3 ± 0.20	1.7 ± 0.03	1.9 ± 0.30	**0.0008**	**0.04**	0.28	1.4
**RAs (mm^2)**	2.9 ± 0.70	2.8 ± 0.60	2.70 ± 0.50	3.40 ± 0.50	0.75	0.15	0.68	0.4
**RAd (mm^2)**	1.6 ± .04	1.8 ± 0.2	1.7± 0.8	1.8 ± 0.2	**0.03**	0.78	0.28	1.4
**RVs (mm^2)**	6.3 ± .4	8.0 ± 1.4	7.8± 0.9	8.1 ± 0.5	0.11	0.91	0.28	1.4

BW, body weight; EF, ejection fraction; FS, fractional shortening; A'/E', pulse wave ratio between active atrial contraction to mitral valve passive ventricle filling; CO, cardiac output; LVPWs, left ventricular posterior wall in systole; LVPWd, left ventricular wall in diastole; LA , Left atrial size; RAs, Right atrial area in systole; RAd, Right atrial area in diastole; RVs, Right ventricular area in systole**.**

Gene	Primer/Probe	Method
GAPDH-Human	Forward 5’-AGCCACATCGCTCAGACACC-3’<br>Reverse 5’-GTACTCAGCGCCAGCATCG-3’	SYBR
SCN5A-Human	Forward 5’-GAAGAAGCTGGGCTCCAAGA-3’<br>Reverse 5’-CATCGAAGGCCTGCTTGGTC-3’	SYBR
GJA5-Human	Forward 5’-GAAGAAGCTGGGCTCCAAGA-3’<br>Reverse 5’-CATCGAAGGCCTGCTTGGTC-3’	SYBR
CACNA1C-Human	Forward 5’-CCAACCTCATCCTCTTCTTCA-3’<br>Reverse 5’-ACATAGTCTGCATTGCCTAGGAT-3’	SYBR
NOX2-Human	Forward 5’- CTCCTGCAACCCGAGAAAGAC -3’<br>Reverse 5’- TGCTCGAAAACTTCCACAATGA –3’	SYBR
PITX2-Human	Forward 5’-AAGGCGAGGATGAGATCCAG-3’<br>Reverse 5’-TTCTTTGTGGATGGTCGCCG-3’	SYBR
TBX5-Human	Forward 5’- CTGTGGCTAAAATTCCACGAAGT -3’<br>Reverse 5’- GTGATCGTCGGCAGGTACAAT -3’	SYBR
Human TBX3	Forward 5’-TGTACATTCACCCGGACAGC-3’<br>Reverse 5’-TTGGGAAGGCCAAAGTAAATCCA-3’	SYBR
Kcnq1-Mouse	Forward 5’- CGCGGTGGTCAAGAAGTGT -3’<br>Reverse 5’- GCACTGTAGATGGAGACCCG -3’	SYBR
Kcne1-mouse	Forward 5’- TCGTCCCTGACCCTTTCGT -3’<br>Reverse 5’- GATAAGCGTCGTTACCCTTGG -3’	SYBR
Kcnj3-mouse	Forward 5’- GGGGACGATTACCAGGTAGTG -3’<br>Reverse 5’- CGCTGCCGTTTCTTCTTGG -3’	SYBR
Kcna5-mouse	Forward 5’- TCCGACGGCTGGACTCAATAA -3’<br>Reverse 5’- CAGCTCCTGAGGCATAGGG -3’	SYBR
Anp-mouse	Forward 5’- ACCTCCCGAAGCTACCTAAGT -3’<br>Reverse 5’- CAACCTTTTCAACGGCTCCAA -3’	SYBR
Gapdh-mouse	Forward 5’- AATGGATTTGGACGCATTGGT -3’<br>Reverse 5’-	SYBR

	TTTGCACTGGTACGTGTTGAT<br>-3’	

Table S3: Primers used for RT-PCR to determine relative expression levels in iPSC-aCMs and human atrial tissue. HAT

Antibody	Antibody vendor	Dilution	Experiment
anti-cTnT polyclonal (rabbit)	proteintech (15513-1-AP)	1:500	Western Blot
anti-cTnI polyclonal (rabbit)	abcam (ab47003)	1:200	Western Blot
anti-Kv1.5 polyclonal (rabbit)	Alomone (APC-150)	1:200	Western Blot
anti-Nav1.5 polyclonal (rabbit)	Alomone (ASC-005)	1:200	Western Blot
anti-Kv7.1 polyclonal (rabbit)	alomone (APC-022)	1:200	Western Blot
anti-Nox4 polyclonal (rabbit)	Proteintech (14347-1-AP)	1:1000	Western Blot
anti-KCNQ1 polyclonal (rabbit)	alomone (APC-022)	1:500	Western Blot
Anti- PKA C-α (rabbit)	Cell Signaling Technology (4872)	1:1000	Western Blot
anti-mouse HRP	Cell Signaling Technology (7076)	1:1500	Western Blot
anti-Myom1 polyclonal (rabbit)	proteintech (22084-1-AP)	1:500	Western Blot
anti-Mybpc3 monoclonal (rabbit)	proteintech (67608-1-Ig)	1:500	Western Blot
anti-Myl7 polyclonal (rabbit)	abcam (ab127001)	1:500	Western Blot
Anti-PKC-δ polyclonal (rabbit)	abcam (ab182126)	1:1000	Western Blot
Anti-PKC-α polyclonal (rabbit)	abcam (ab4124)	1:1000	Western Blot
Anti-Fabp3 polyclonal (rabbit)	Proteintech (10676-1-AP)	1:1000	Western Blot
Anti-Cpt1α polyclonal (rabbit)	abcam (ab128568)	1:1000	Western Blot
Anti-PPArα monoclonal (mouse)	Santa Cruz Biotechnology (sc-398394)	1:1000	Western Blot
anti-Mlc2v polyclonal (rabbit)	abcam (ab92721)	1:500	Western Blot
anti-rabbit HRP	Cell Signaling Technology (7076)	1:1500	Western Blot
anti-Actin HRP	Santa Cruz Biotechnology (sc-47778 HRP)	1:2500	Western Blot
Anti-Pitx2 monoclonal (mouse)	Santa Cruz Biotechnology (sc-390457)	1:1000	Western Blot
Anti-Kir3.1 polyclonal (mouse)	Santa Cruz Biotechnology (sc-50410)	1:500	Western Blot

Table S4: Antibodies used in western blots and immunofluorescence to probe protein expression levels and localization.

Supplementary Figure S1

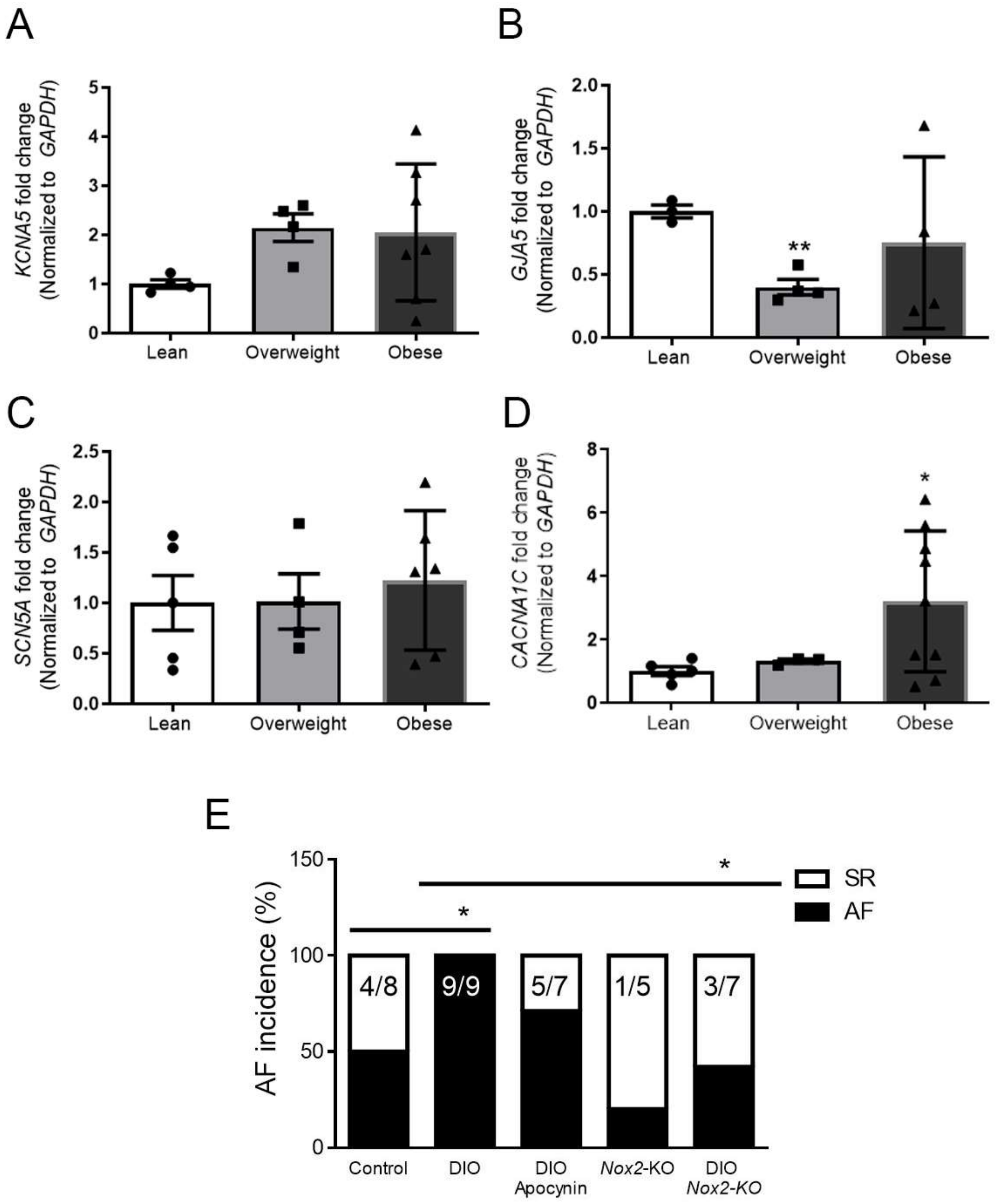

Figure S1. Expression of ion channel and structural genes in atrial tissues from lean, overweight, and obese individuals. (A) qPCR data showing the transcriptional level of Human Potassium Voltage-Gated Channel Subfamily A Member 5 (*KCNA5*), encoding for I_{Kur} current from

lean (n=4), overweight (n=4), and obese individuals (n=7). **(B)** Human gap junction alpha protein encoding for atrial specific connexin 40 (*GJA5*) current from lean (n=3), overweight (n=4), and obese individuals (n=4). **(C)** Human cardiac sodium channel type V α-subunit (*SCN5A*) lean (n=5), overweight (n=4), and obese individuals (n=6) **(D)** Calcium Voltage-Gated Channel Subunit Alpha1 C (*CACNA1C*), encoding for the L -type calcium current protein lean (n=6), overweight (n=3), and obese individuals (n=9). **(E)** Incidence of AF (%) in Control (N=8), DIO (N=9), DIO-Apocynin (N=7), *Nox2*-KO (N=5), and DIO *Nox2*-KO (N=7) mice. P>0.05; *P<0.05; **P<0.01; ***P<0.001; ****P<0.0001, by 2-tailed, unpaired Student's *t* test.

Supplementary Figure S2

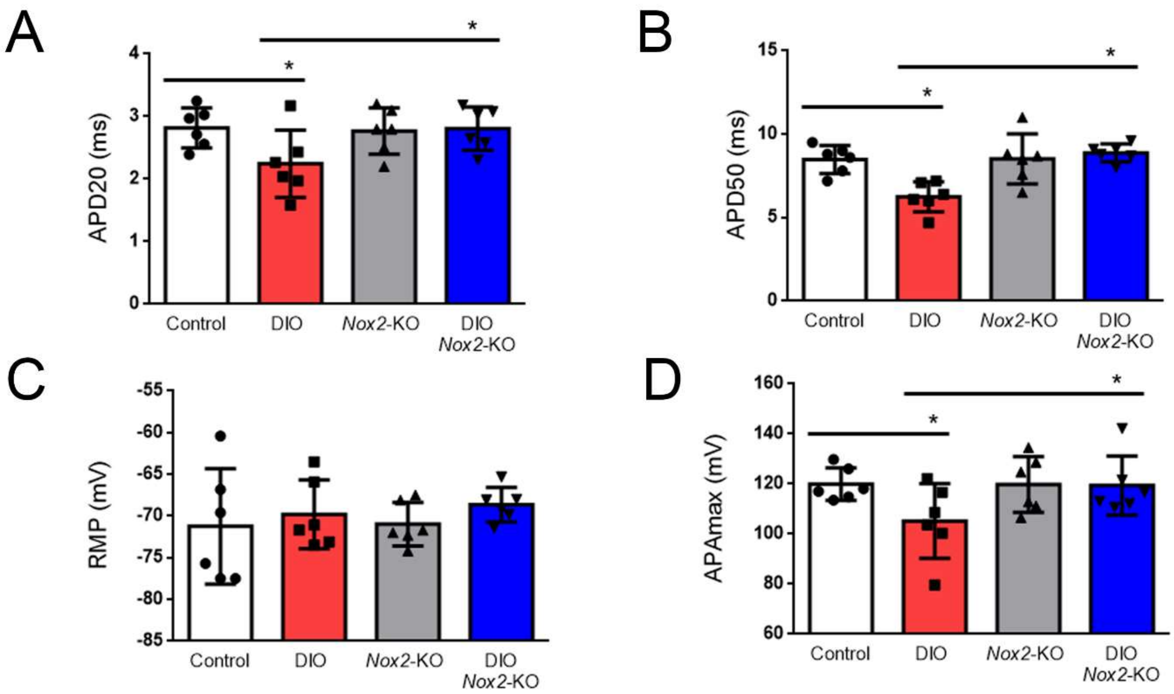

Figure S2. Measured AP parameters in control (n=6 cells, N= 4 mice), DIO (n=6 cells, N= 4 mice), *Nox2*-KO (n=6 cells, N= 3 mice), and DIO-*Nox2*-KO mice (n=6 cells, N= 3 mice). (A) Action potential duration (APD) at 20% repolarization (APD20). **(B)** APD at 50% repolarization (APD50). **(C)** Resting membrane potential (RMP). **(D)** Maximum action potential amplitude (APA_{max}). P>0.05; *P<0.05; **P<0.01; ***P<0.001; ****P<0.0001, by 2-tailed, unpaired Student's *t* test.

Supplementary Figure S3

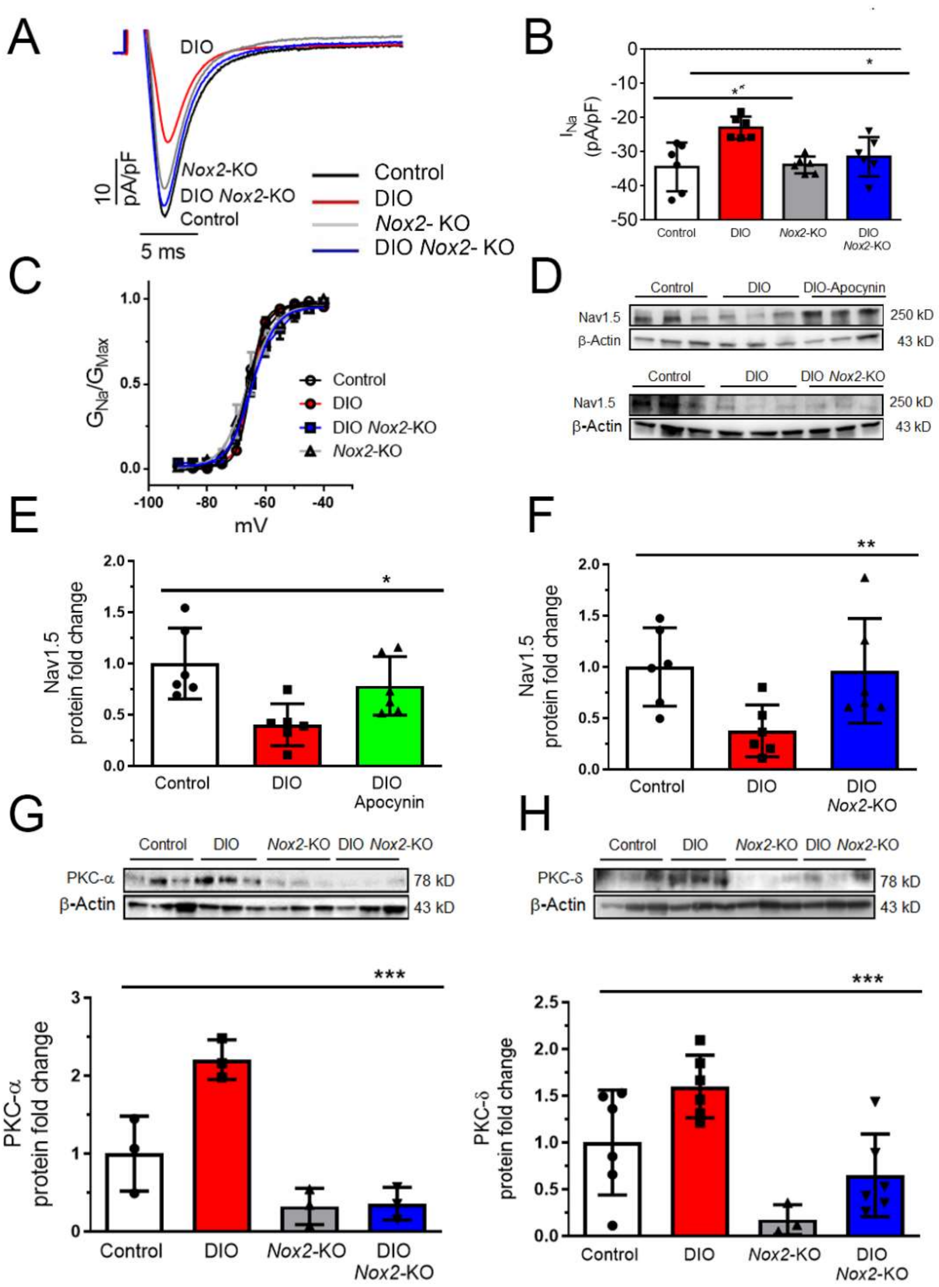

Figure S3. Normalized I_{Na} in DIO *Nox2*-KO compared to DIO mice. (A) Representative I_{Na} tracings from all 4 groups of mice showing increased I_{Na} in DIO *Nox2*-KO mice versus DIO mice. (B) Peak sodium (I_{Na}) current density in control, DIO, *Nox2*-KO, and DIO *Nox2*-KO mice

(n=6 cells, N= 3 mice each). (C) Activation curve of I_{Na} current in the 4 groups of mice. (D) Western blot showing cardiac sodium channel type 5 alpha subunit ($Na_v1.5$) expression in atria of control, DIO, DIO-Apocynin, and *Nox2*-KO DIO mice (N=6 each). (E-F) Quantification of $Na_v1.5$ expression among the 4 groups (N=6 each). (G) Western blot and quantification showing PKC-α expression in atria of control, DIO, *Nox2*-KO and *Nox2*-KO-DIO mice (N=3 each). (H) Western blot and quantification showing PKC-δ expression in atria of control, DIO, DIO-*Nox2*-KO mice (N=6 each), and *Nox2*-KO (N=3). One-way ANOVA was used to compute statistical significane in Panels E-H. *P<0.05; **P<0.01; ***P<0.001.

Supplementary Figure S4

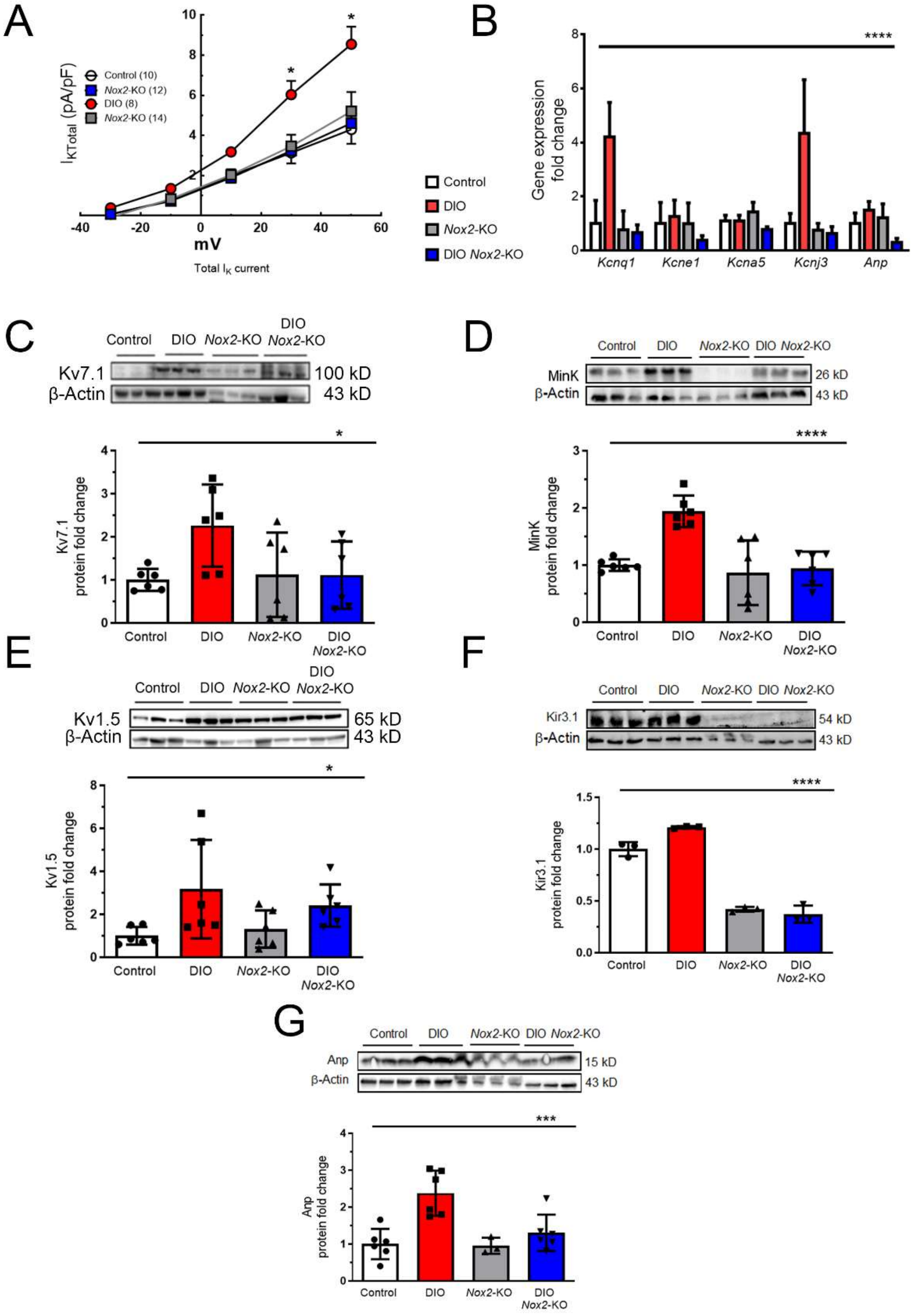

Figure S4. Increased expression of K+ channels in DIO-*Nox2*-KO compared to DIO mice. (A) Total potassium (K^+) current (I_K) current and voltage relationship (I–V curves) in control (N=10), DIO (N=8), *Nox2*-KO (14) and DIO *Nox2*-KO mice (N=12). (B) qPCR data on K^+ channel genes- *Kcnq1*, *Kcne1*, *Kcna5*, *Kcnj3* and *Anp* in the 4 mouse groups (N=3 each). (C) Western blot and quantification of showing I_{Ks} encoding Kv7.1 in control, DIO, *Nox2*-KO, and DIO *Nox2*-KO mice (N=6 each). (D) Western blot and quantification of showing I_{Ks} encoding minK in the 4 mouse groups (N=6 each). (E) Western blot and quantification of showing Kv1.5 encoding in the 4 mouse groups (N=6 each). (F) Western blot and quantification of showing Kir3.1 encoding *Kcnj3* in the 4 mouse groups (N=3 each). (G) Western blot and quantification of showing Anp in the Control, DIO, DIO *Nox2*-KO mouse groups (N=6 each) and *Nox2*-KO (N=3). Two-way ANOVA was used in qPCR analysis in Panel B. One-way ANOVA was used in western blot analyses in panels C-G. *P<0.05; **P<0.01; ***P<0.001; ****P<0.0001.

Supplementary Figure S5

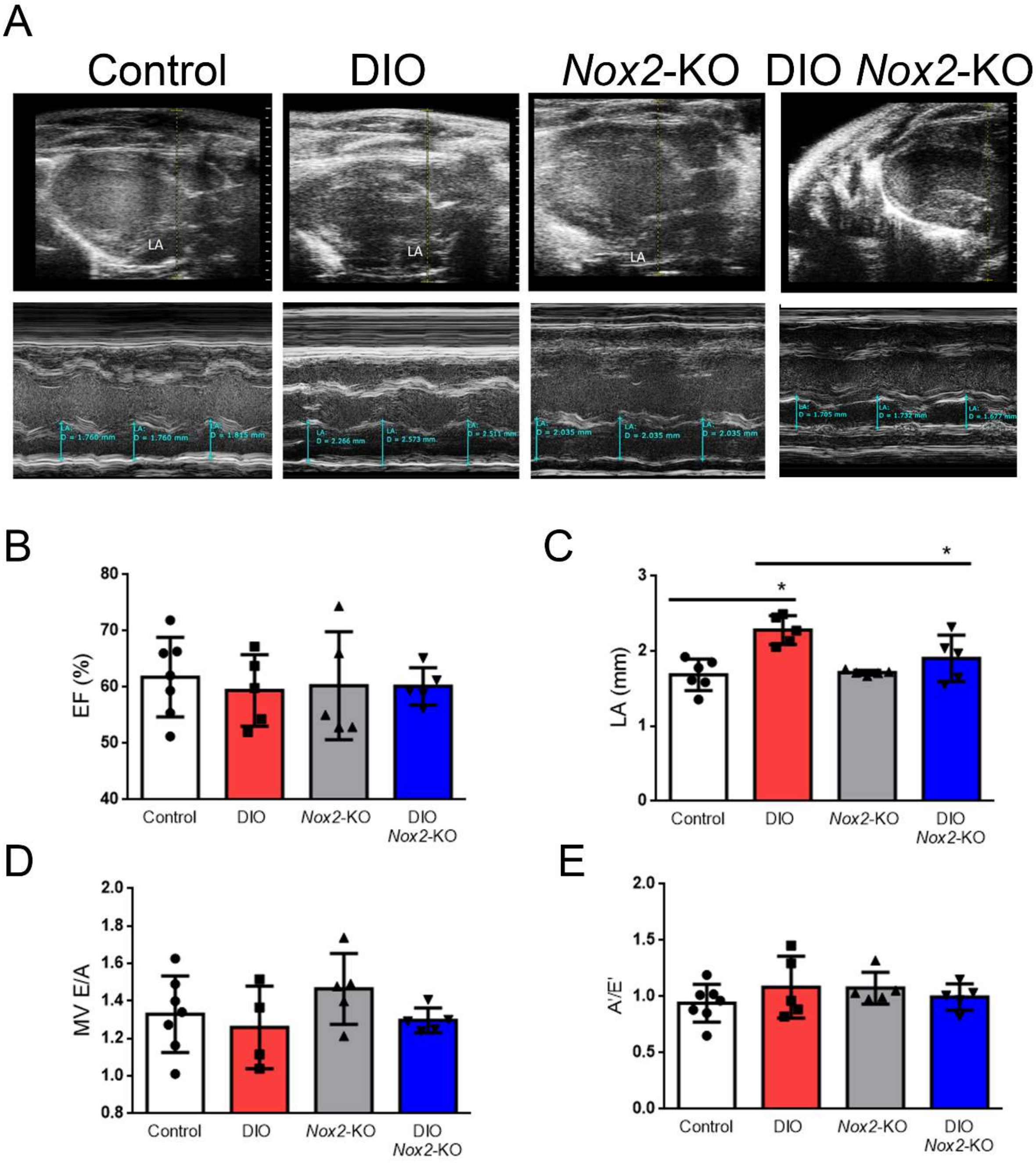

Figure S5. Left atrial (LA) enlargement in DIO mice is reduced after Nox2 inhibition. **(A)** Representative long axis (LAX) images of control (n=7), DIO (n=5), *Nox2*-KO (n=5) and DIO

Nox2-KO mice (n=5). **(B)** Quantified ejection fraction (EF) in the 4 groups of mice each. **(C)** LA diameter in control, DIO, and *Nox2*-KO DIO mice. **(D)** Quantified doppler ratio between mitral valve active atrial contraction to passive ventricle filling (E'/A'). **(E)** Quantified doppler ratio between passive ventricle filling to atrial contraction. 1-way ANOVA test- P>0.05; *P<0.05; **P<0.01

Supplementary Figure S6

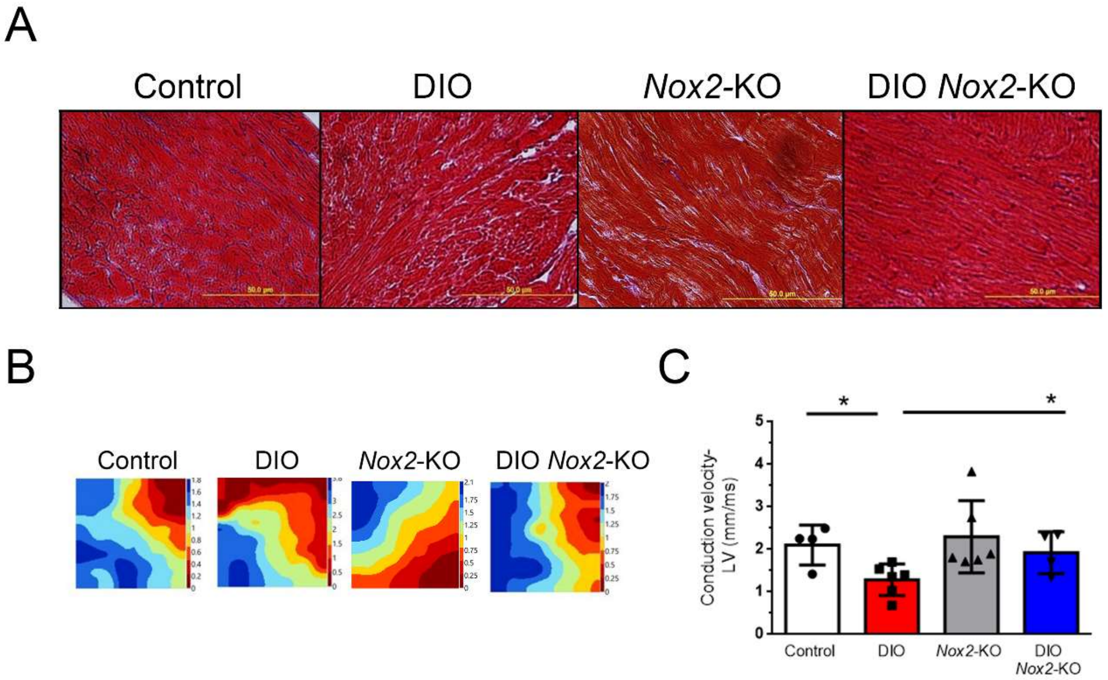

Figure S6. Effect of Nox2 inhibition on left ventricular fibrosis and conduction velocity (CV) in DIO mice. (A) Masson trichrome staining of ventricular myocytes from control, DIO, and DIO-*Nox2*-KO mice. **(B)** Representative isochronal maps of the left ventricle in using electrical mapping in control (N=4 mice), DIO (N=6 mice), *Nox2*-KO (N=6) , and DIO *Nox2*-KO (N=4) **and** quantification of mean left ventricular CV *P<0.05; **P< 0.01; ****P<0.0001, by 2-tailed, unpaired Student's *t* test.

Supplementary Figure S7.

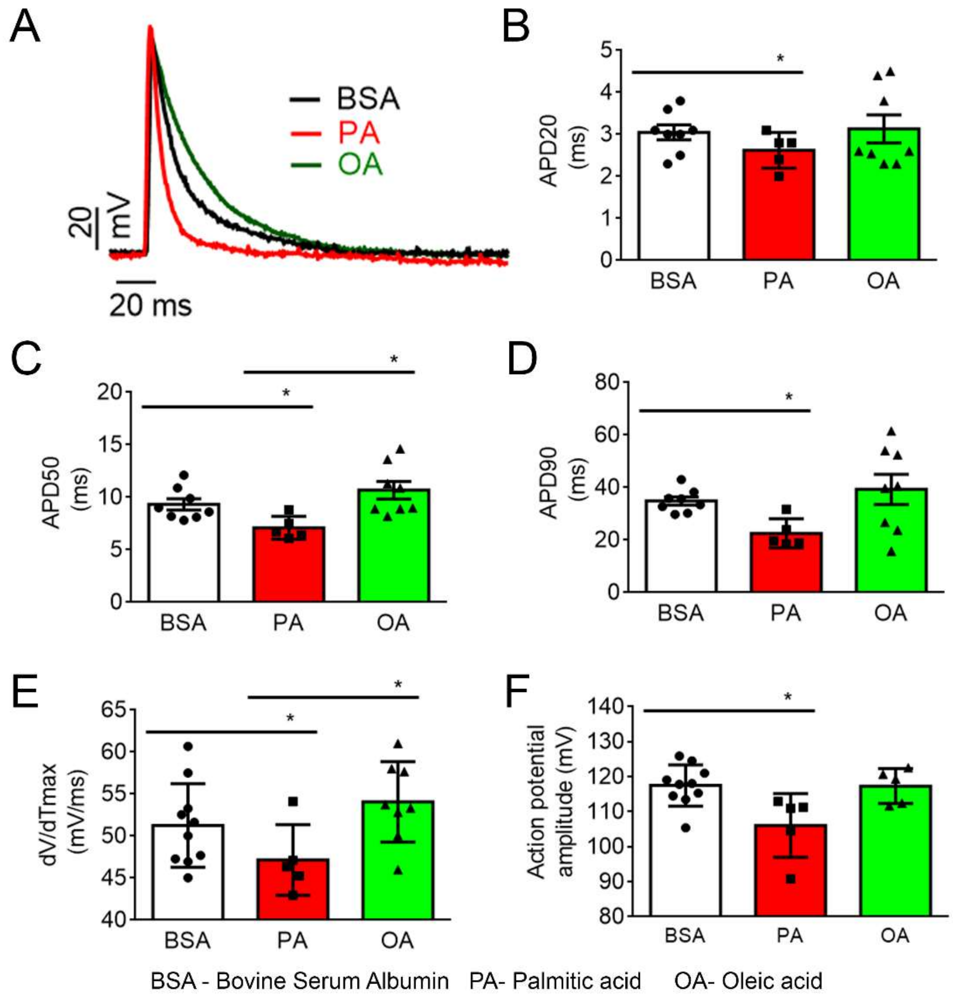

Figure S7. Treatment of hiPSC-aCMs with palmitic acid (PA) shortens the atrial AP compared to bovine serum albumin (BSA) and oleic acid (OA) treated-hiPSC-aCMs: (**A**) Whole-cell patch-clamping of BSA, PA, and OA treated-hiPSC-aCMs. **(B)** Measured APD at 20% repolarization (APD20) in BSA (n=8), PA (n=5), and OA treated-hiPSC-aCMs (n=5 cells). **(C)** Measured APD at 50% repolarization (APD50) in BSA (n=8), PA (n=5), and OA treated-hiPSC-aCMs (n=5 cells) **(D)** Measured APD at 90% repolarization (APD90) in BSA (n=8), PA (n=5), and OA treated-hiPSC-aCMs (n=5 cells) **(E)** Instantaneous rate of voltage change over time (dV/dT_{max}), an indicator of atrial conduction velocity (CV) in BSA (n=10), PA (n=5), and OA

treated-hiPSC-aCMs (n=5 cells). (**F)** Maximum atrial potential amplitude (APAmax) in BSA (n=10), PA (n=5), and OA treated-hiPSC-aCMs (n=5 cells). P>0.05; *P<0.05; **P<0.01; ***P<0.001; ****P<0.0001, by 2-tailed, unpaired Student's *t* test.

Supplementary Figure S8.

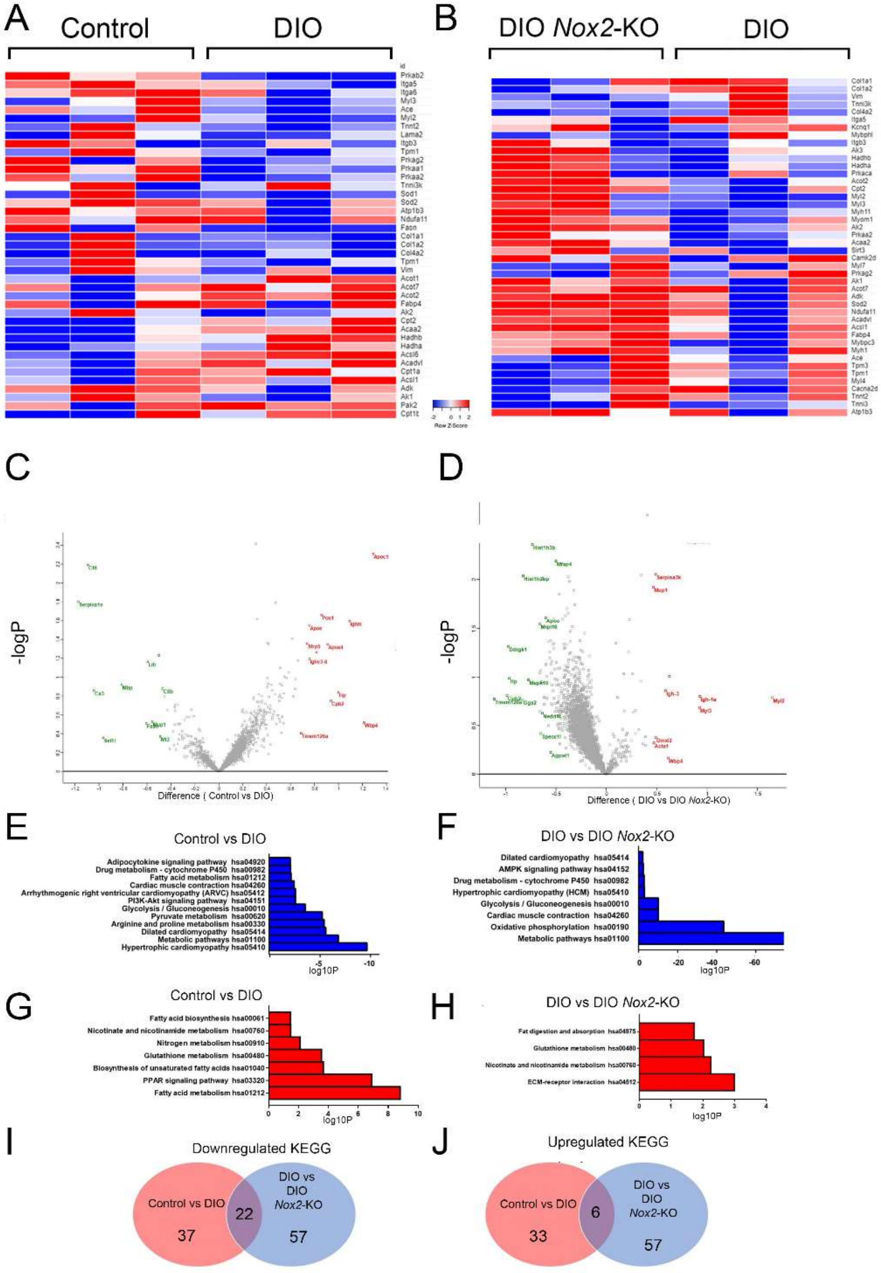

Figure S8. Global quantitative proteomics and pathway enrichment analysis in the control, DIO, and DIO *Nox2*-KO atrial lysates. (**A**) Heatmap of cardiac related upregulated and downregulated proteins in control (no HFD) versus DIO (N=3 mice each). (**B**) Heatmap of cardiac related upregulated and downregulated proteins in DIO versus DIO-*Nox2*-KO atrial lysates (N=3

each). (**C-D**) Volcano plots showing both significantly upregulated and downregulated proteins in both comparisons. (**E-F**) Major downregulated pathways using Kyoto Encyclopedia of Genes and Genomes (KEGG) pathway enrichment analysis. (**G-H**) Major upregulated KEGG pathways in both comparisons. (**I-J**) Venn diagrams showing common upregulated and downregulated pathways between Control vs DIO and DIO vs DIO *Nox2*-KO comparisons.

Supplementary Figure S9.

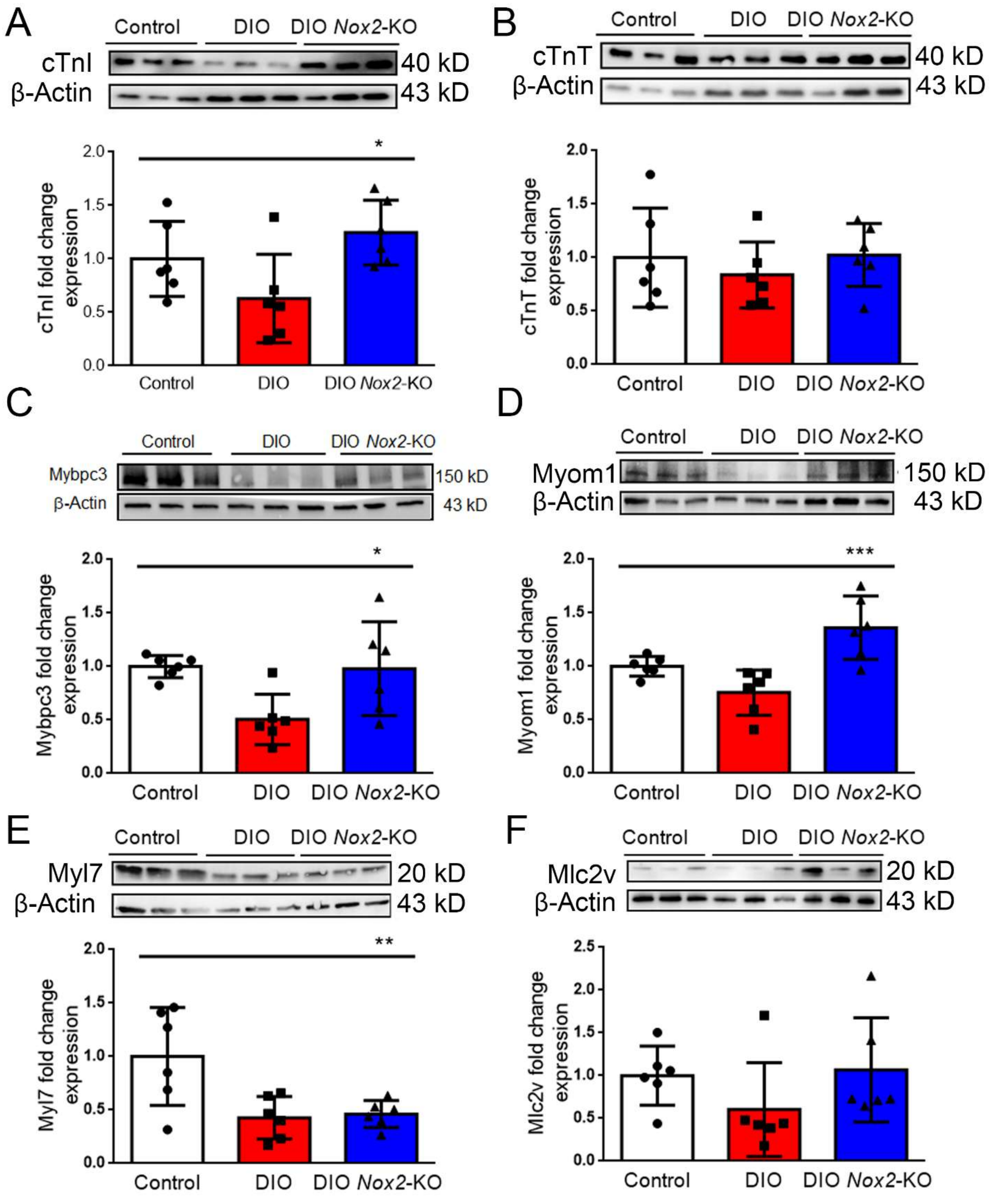

Figure S9. Western blot and quantification of proteins involved in the **Cardiac muscle contraction pathway (hsa04260)**. **(A)** Cardiac troponin T (**cTnT**) **)** in Control, DIO, and DIO *Nox2*-KO (N=6 each). **(B)** Cardiac troponin I (**cTnI**) in Control, DIO, and DIO *Nox2*-KO (N=6 each). **(C)** Cardiac myosin binding protein C **(Mybpc3)** in Control, DIO, and DIO *Nox2*-KO (N=6 each). **(D)** Myomesin 1 **(Myom1)** in Control, DIO, and DIO *Nox2*-KO (N=6 each). **(E)** Myosin Light Chain 7 **(Myl7)** in Control, DIO, and DIO *Nox2*-KO (N=6 each). **(F)** Myosin Light Chain 2 **(Mlc2v)** in Control, DIO, and DIO *Nox2*-KO (N=6 each). Western blot and quantification of **Fatty acid metabolism pathway proteins (hsa0061).** One-way ANOVA was used to compute statistical significance. *P<0.05; **P<0.01; ****P<0.0001.

Supplementary Figure S10.

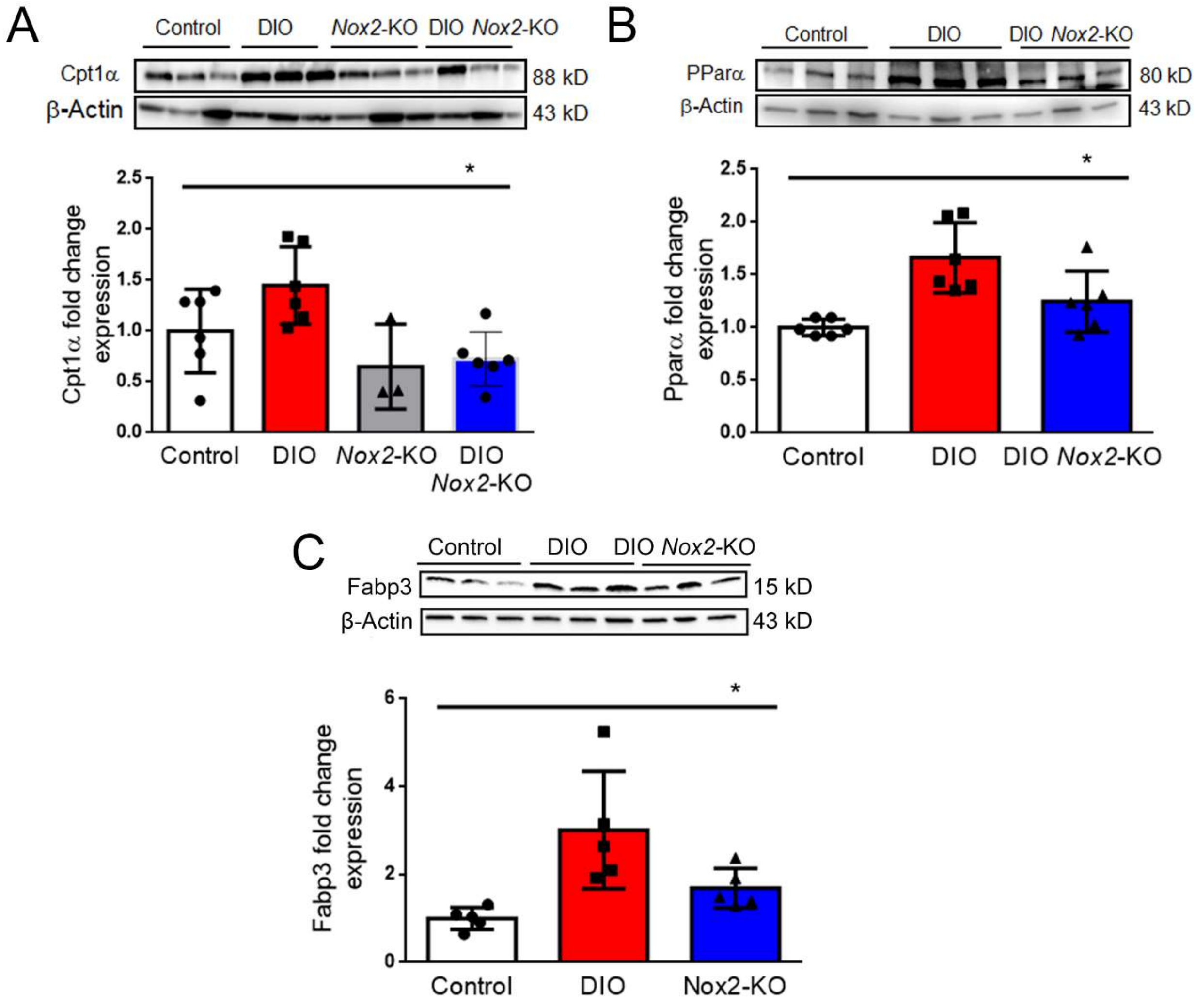

Figure S10. Western blot and quantification of proteins involved in the **Fatty acid metabolism: (A)** Carnitine palmitoyltransferase 1A **(Cpt1α) in** Control, DIO, DIO *Nox2*-KO (N=6 each), and *Nox2*-KO (N=3). **(B)** Peroxisome Proliferator Activated Receptor α **(Pparα)** in Control, DIO, and DIO *Nox2*-KO (N=6 each). (**C**) Heart type Fatty Acid Binding Protein 3 (**Fabp3**) in Control, DIO, and DIO *Nox2*-KO (N=6 each). One-way ANOVA was used to compute statistical significance. *P<0.05; **P<0.01; ****P<0.0001.

Supplementary Figure S11.

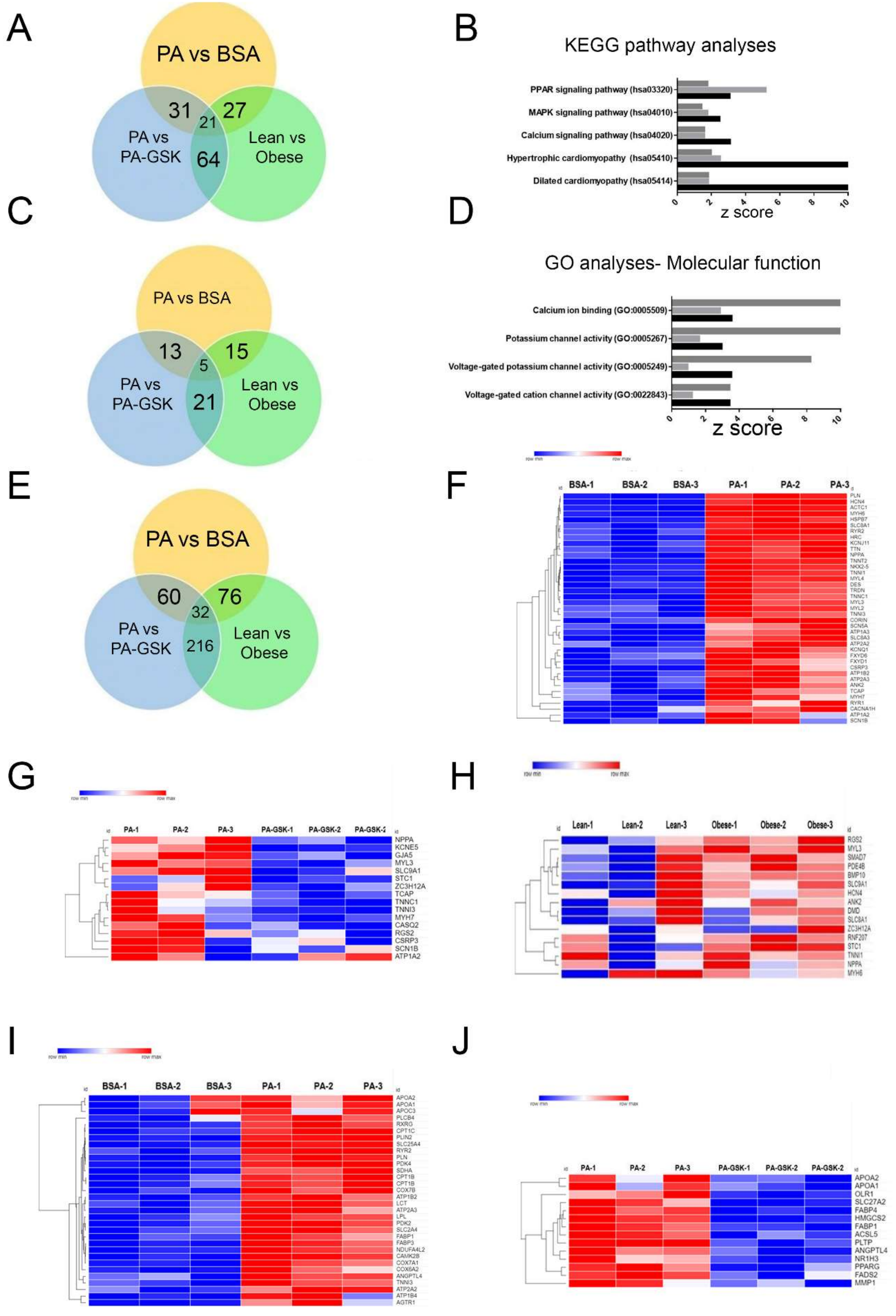

Figure S11. Common RNAseq pathways from hiPSC-aCM and Human atrial tissue data sets **(A)** Venn diagram showing number of common upregulated KEGG pathways between BSA vs PA-hiPSC-aCMs, PA vs PA-GSK-hiPSC-aCMs, and lean vs obese human atrial tissues (HATs). **(B)** Common cardiac related upregulated KEGG pathways between the 3 comparisons. **(C)** Venn diagram showing number of common upregulated gene ontology (GO) molecular function pathways between BSA vs PA-hiPSC-aCMs, PA vs PA-GSK-hiPSC-aCMs, and lean vs obese HATs. **(D)** Common cardiac GO molecular function pathways pathways between the 3 comparisons. **(E)** Venn diagram showing number of common GO biological process pathways between BSA vs PA-hiPSC-aCMs, PA vs PA-GSK-hiPSC-aCMs, and lean vs obese HATs. (**F–H**) Heatmaps of top upregulated and downregulated DEGs associated with the key GO pathway-Regulation of heart contraction (GO0008016) in **(F)** BSA vs PA-hiPSC-aCMs, **(G)** PA vs PA-GSK-hiPSC-aCMs and **(H)** Lean vs Obese HATs. (**I–J**) Heatmaps of top upregulated and downregulated DEGs associated with the key KEGG pathway- PPAR signaling pathway in **(I)** BSA vs PA-hiPSC-aCMs and **(J)** PA vs PA-GSK-hiPSC-aCMs.

Supplementary Figure S12.

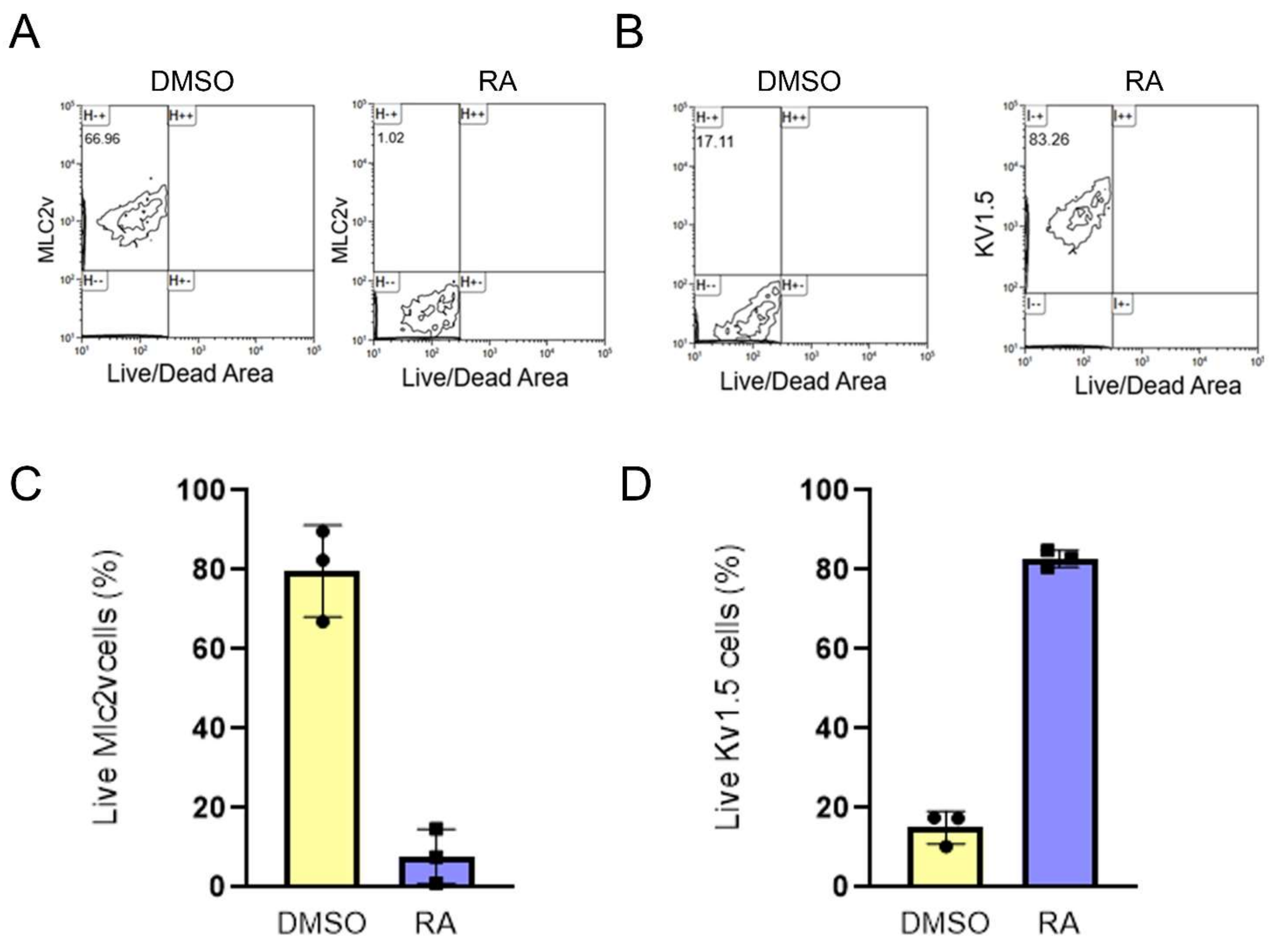

Supplementary Figure S12. A: Representative flow cytometry contour plots of RA-treated and dimethyl sulfoxide (DMSO; Control, N= 3) cells sorted into MLC2v positive and live fractions at day 10 (quadrant 1, upper left). **B**: Representative flow cytometry contour plots of RA-treated and CT cells sorted into Kv1.5 positive and live fractions at day 10 (quadrant 1, upper left). (**C-D**) Averaged flow cytometry data from three biological replicates for live MLC2v (C) and Kv1.5 (D) flow cytometry fractions, respectively, by 2-tailed, unpaired Student's *t* test.
