# Supplementary material for: Modulation of NOX2 causes obesity-mediated atrial fibrillation
Source: J Clin Invest. 2024 Aug 15;134(18):e175447. doi: 10.1172/JCI175447 (PMC11405042; doi:10.1172/JCI175447)
Supplement: Unedited blot and gel images [file jci-134-175447-s127.pdf]

Unedited Figure 7H- Pitx2 blot

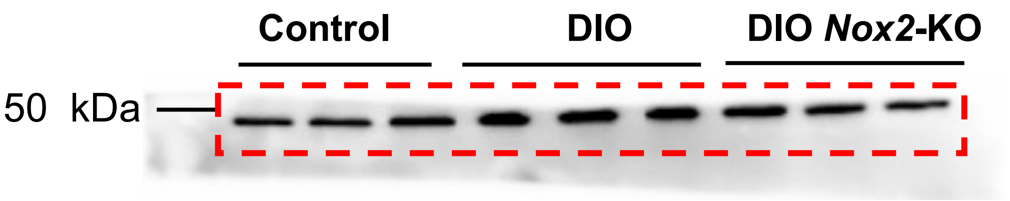

Blot 1- Actin

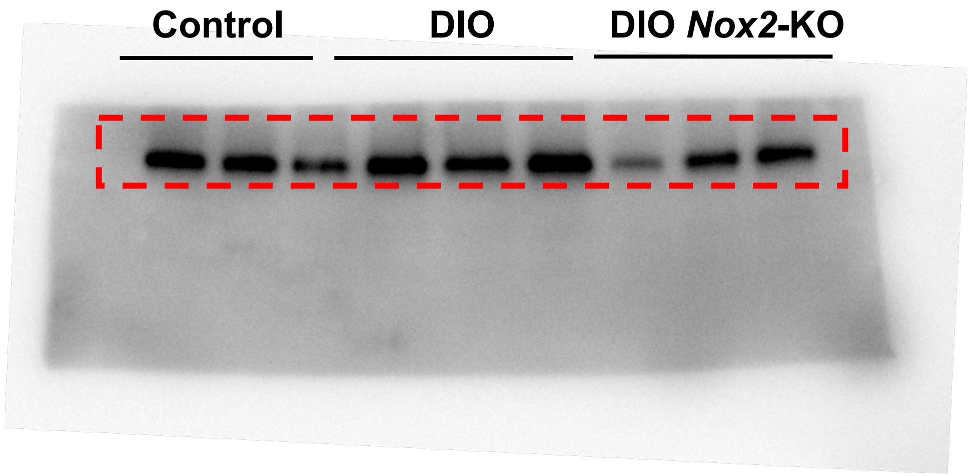

Blot 1- Pitx2

Red box refers to blot used in figure

Unedited Figure S3D- Nav1.5 blots

Blot-1 Actin

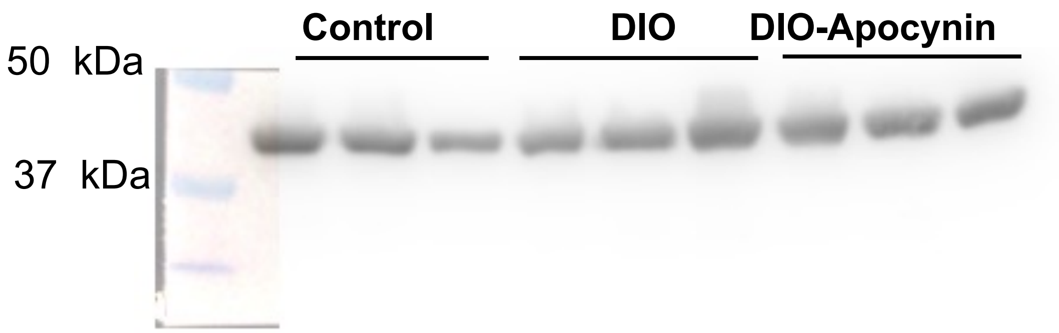

Blot-2 Actin

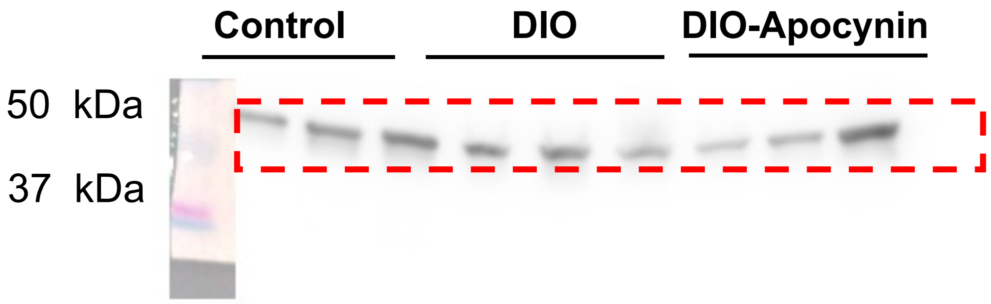

Blot-1 Nav1.5

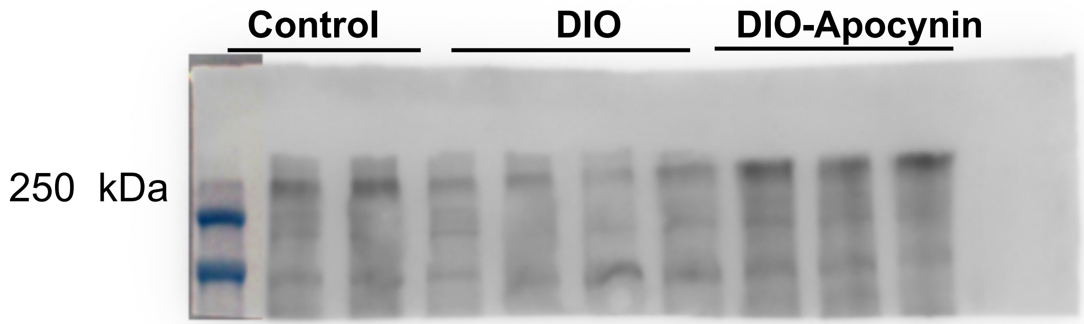

Blot-2 Nav1.5

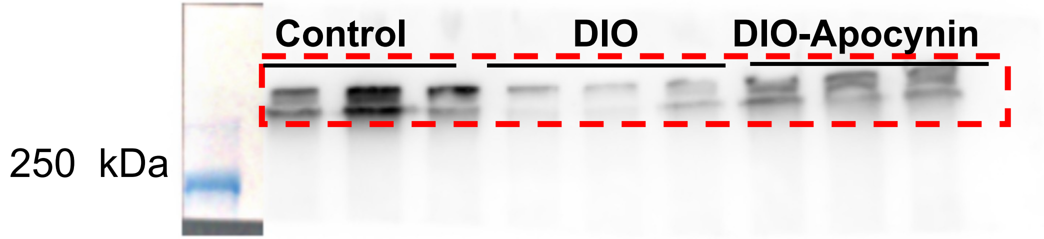

Red box refers to blot used in figure

Unedited Figure S3D/E- Nav1.5 blots

Control DIO DIO *Nox2*-KO

Actin -1

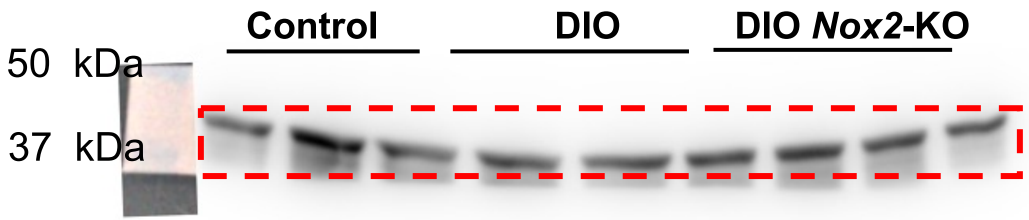

Actin -2

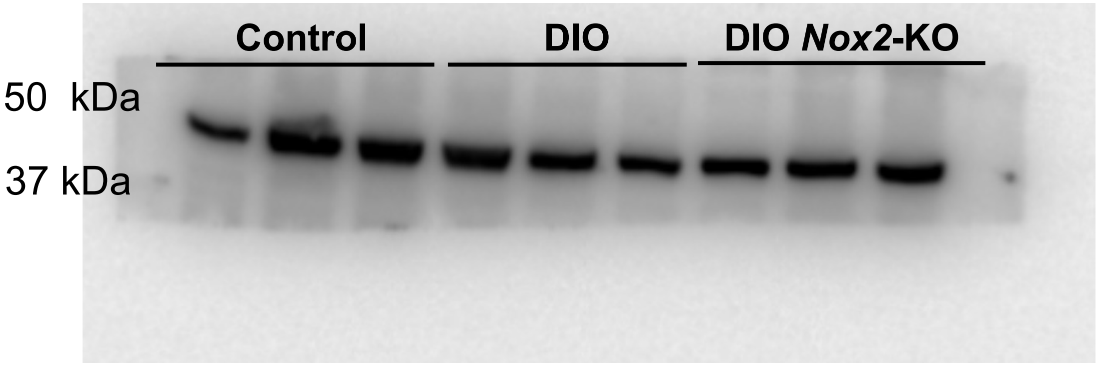

Nav1.5-1

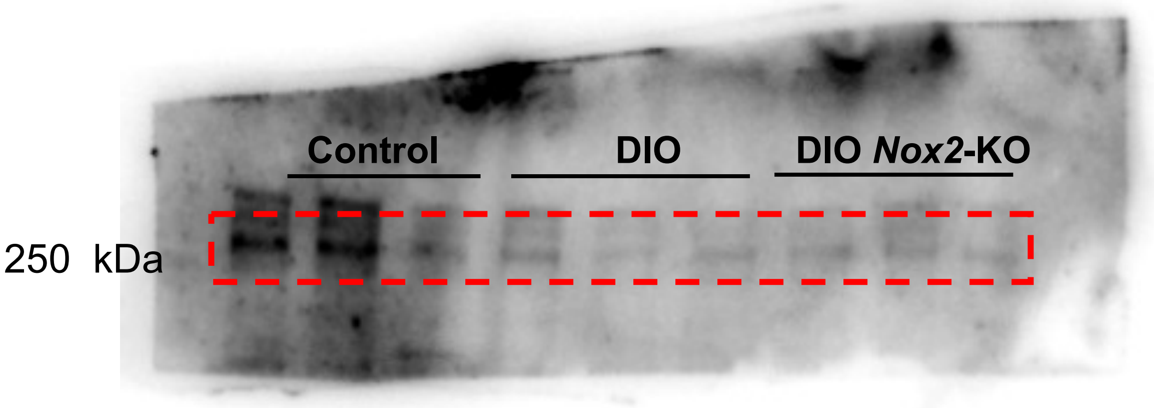

Nav1.5-2

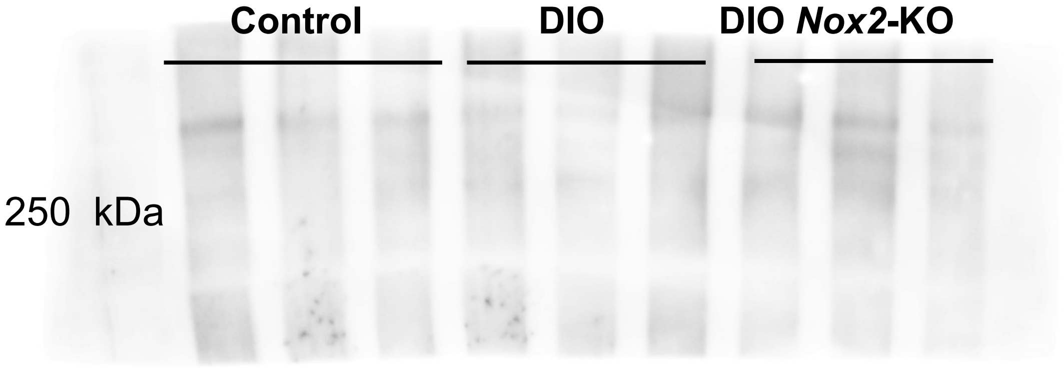

Red box refers to blot used in figure

Unedited Figure S3G- PKC-α blots

Actin

Control DIO *Nox2*-KO DIO *Nox2*-KO

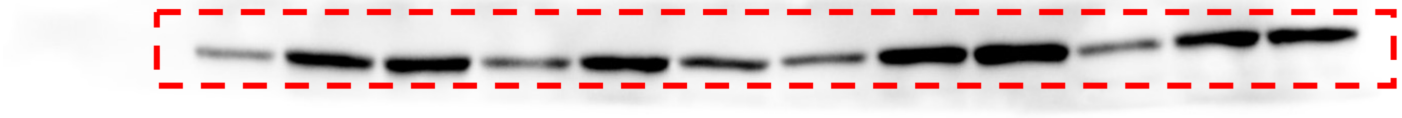

Pkc-a

Control DIO *Nox2*-KO DIO *Nox2*-KO

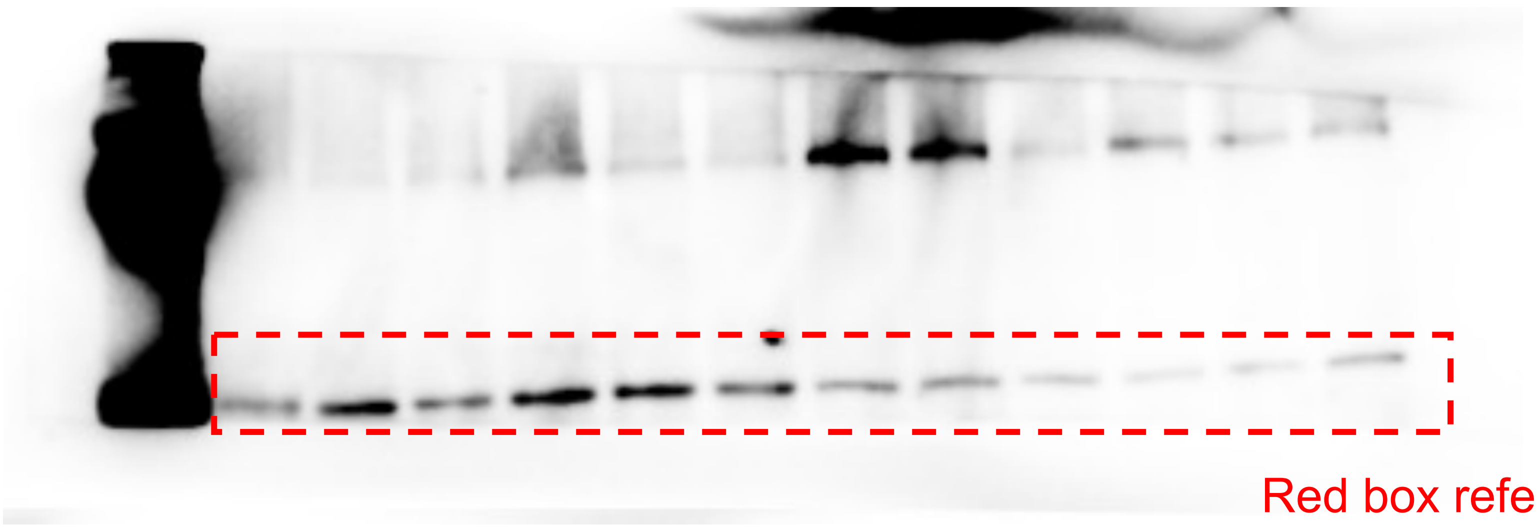

Red box refers to blot used in figure

Unedited Figure S3H- PKC-δ blots

Actin -1

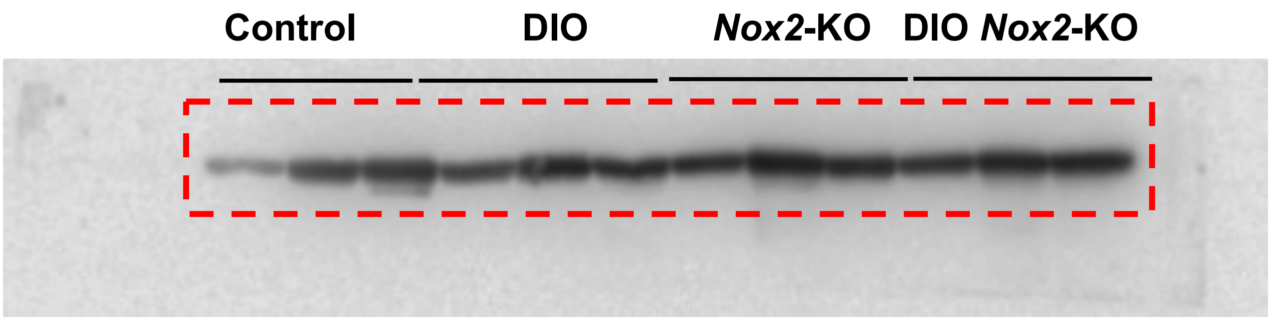

Actin -2

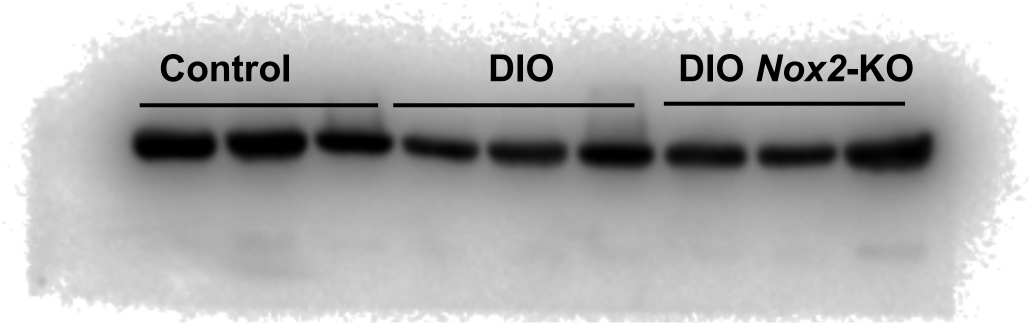

Pkc-d-1

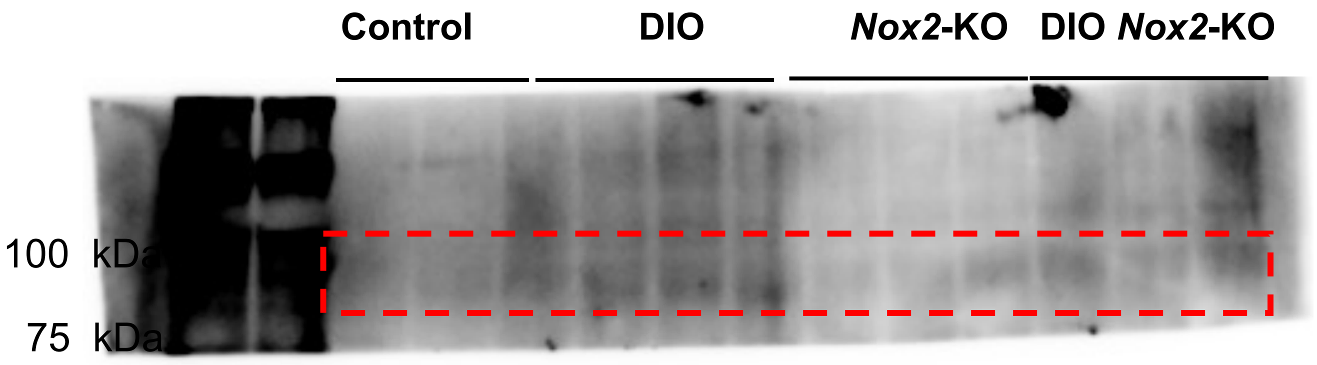

Pkc-d- 2

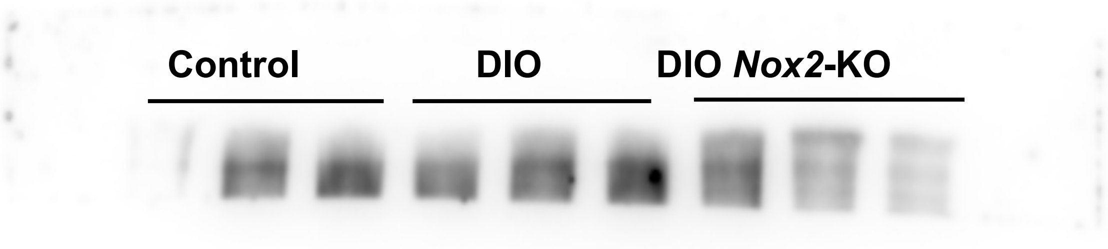

Red box refers to blot used in figure

Unedited Figure S4C- Kv7.1 blots

Actin -1

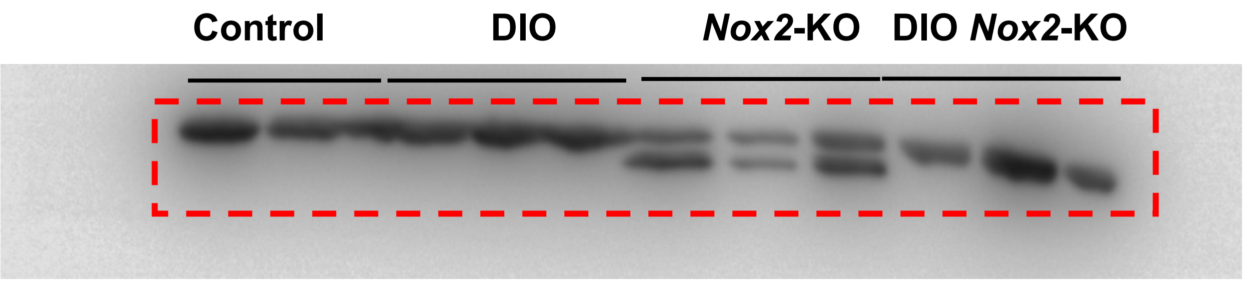

Actin -2

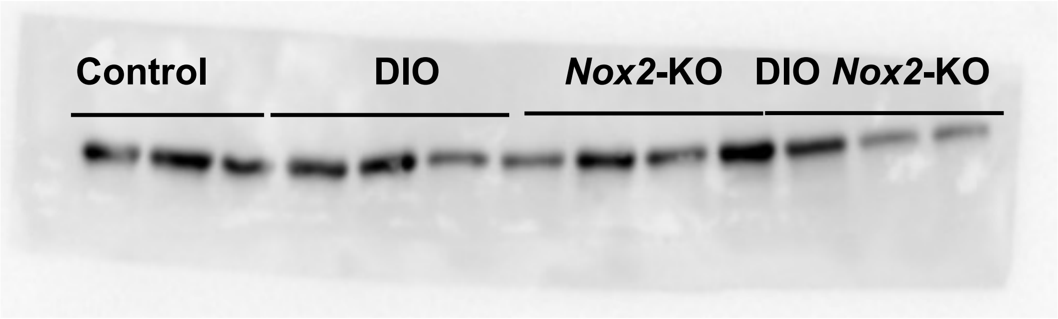

Kv7.1-1

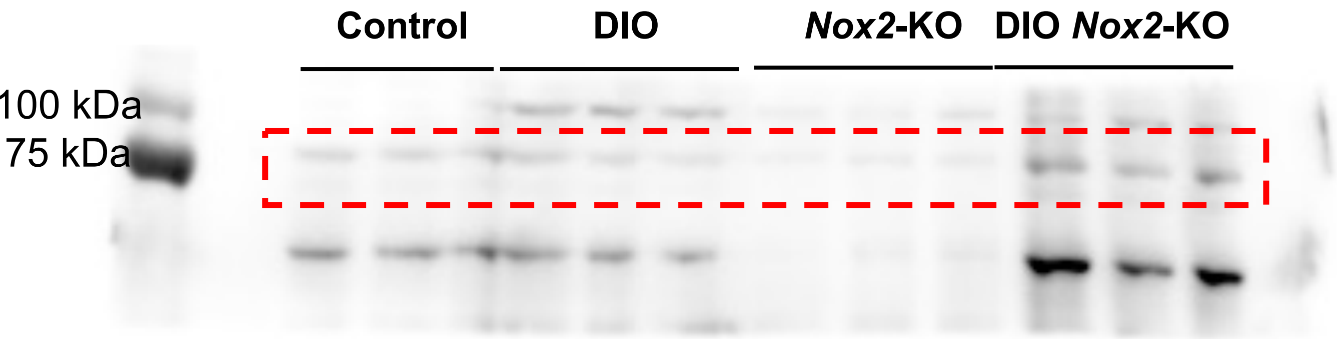

Kv7.1-2

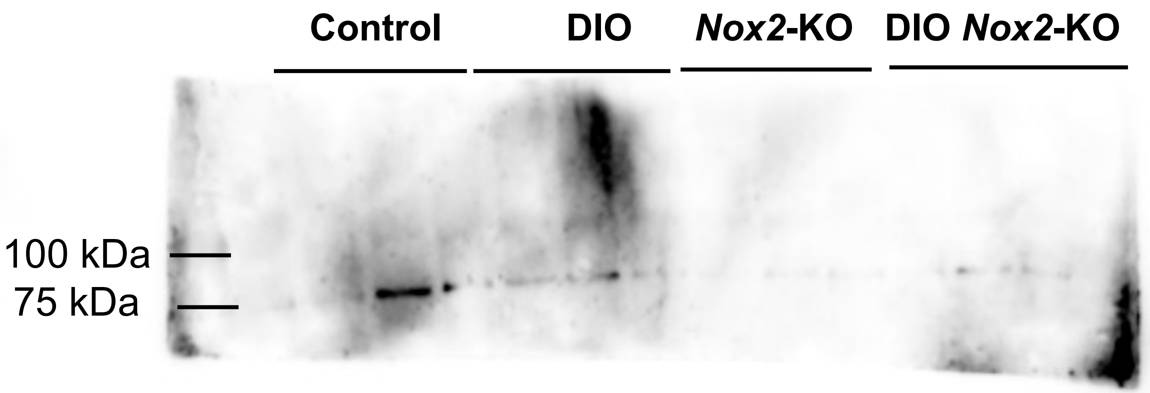

Red box refers to blot used in figure

Unedited Figure S4D- MinK blots

Actin -1

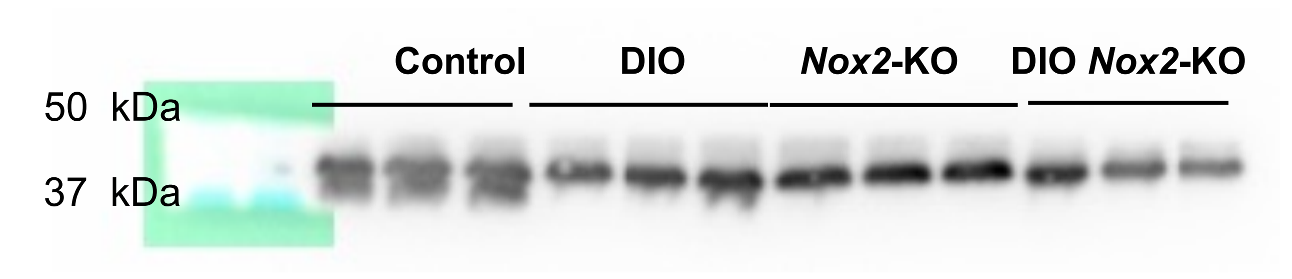

Actin -2

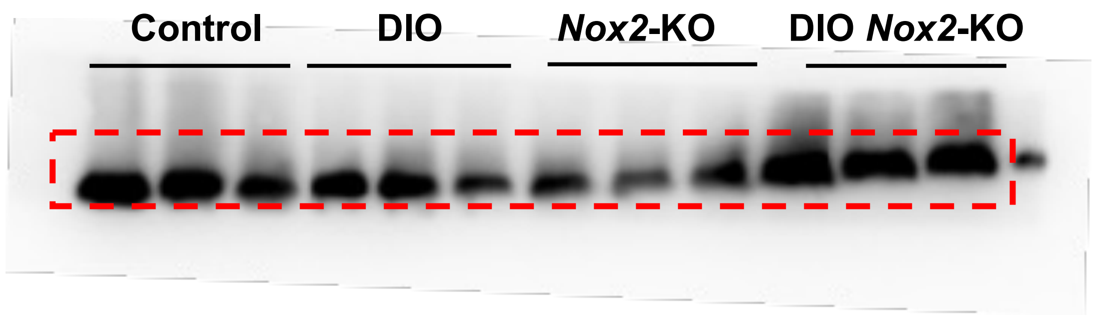

MinK-1

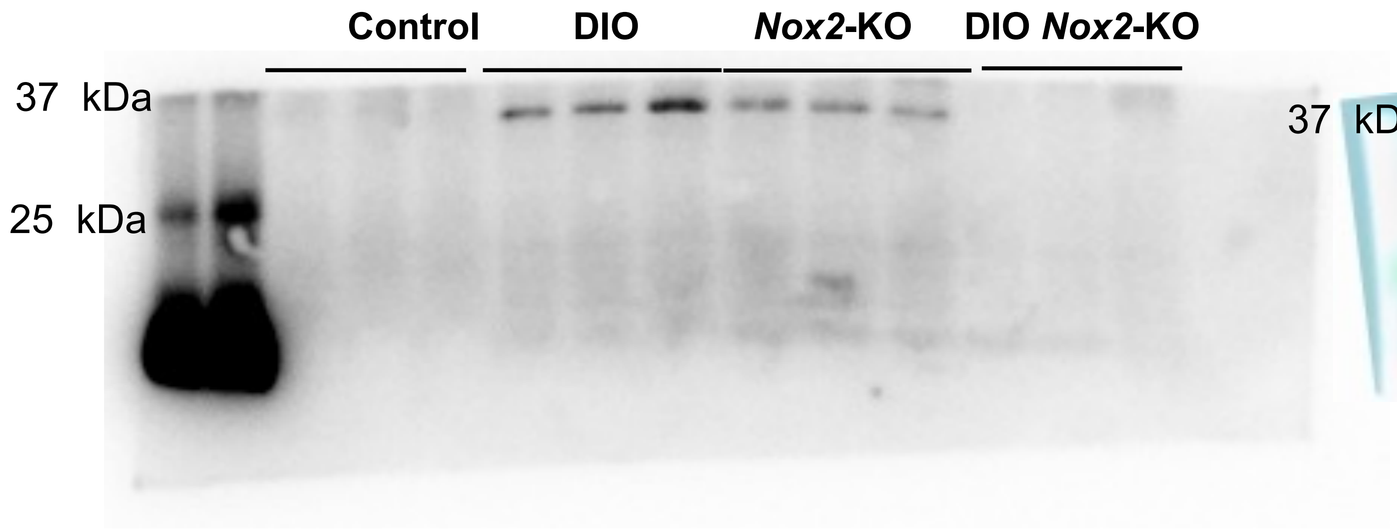

MinK-2

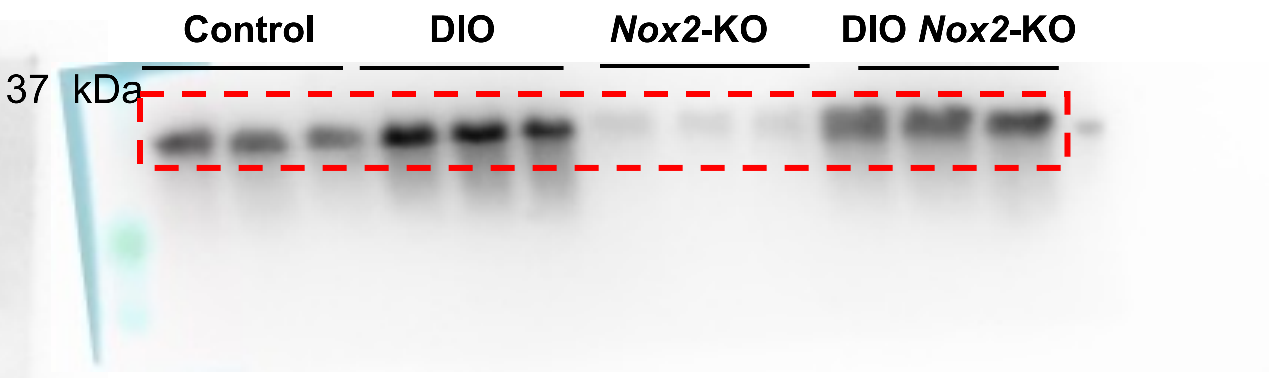

Red box refers to blot used in figure

Unedited Figure S4E- Kv1.5 blots

Actin -1

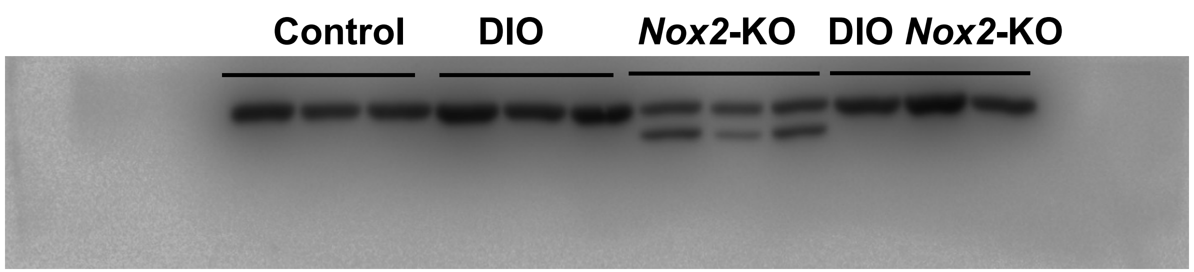

Actin -2

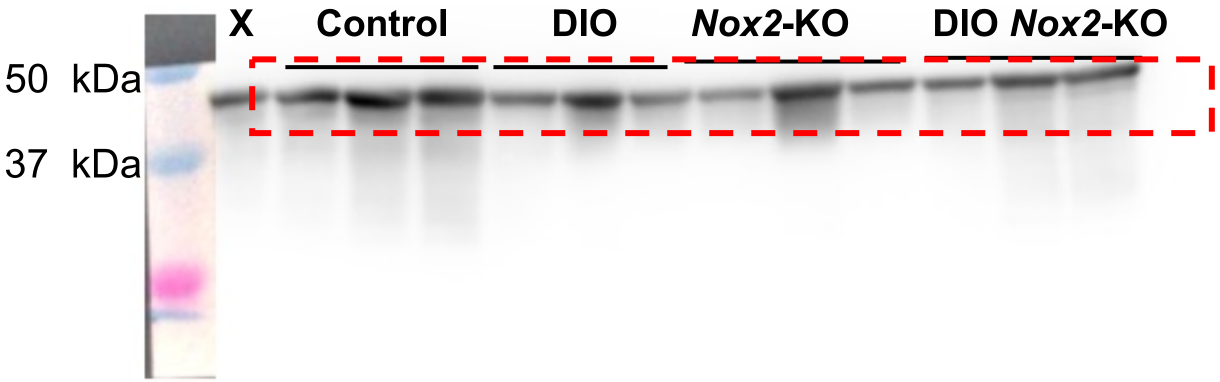

Kv1.5-1

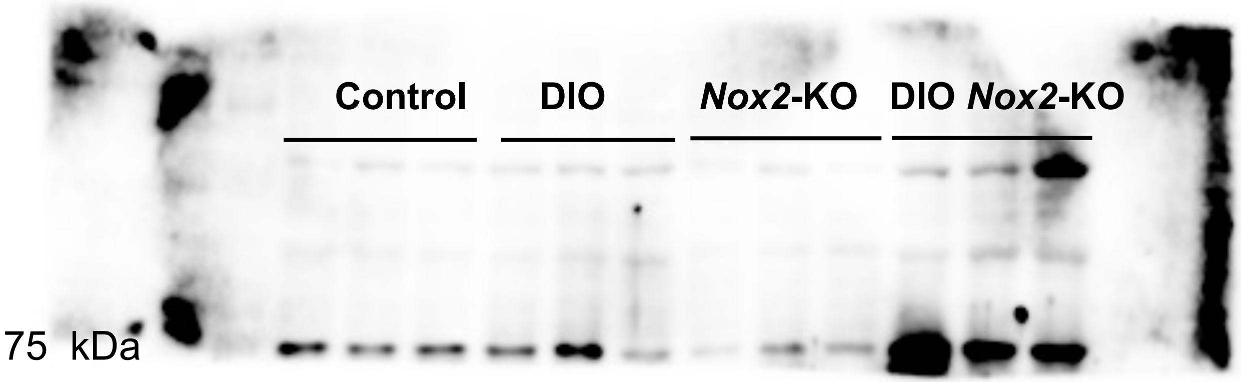

Kv1.5-2

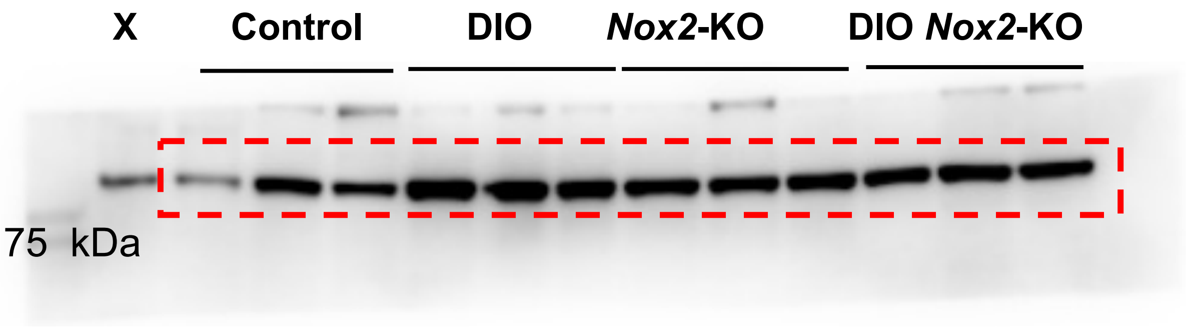

Red box refers to blot used in figure

Unedited Figure S4F- Kir3.1 blot

Blot 1- Actin

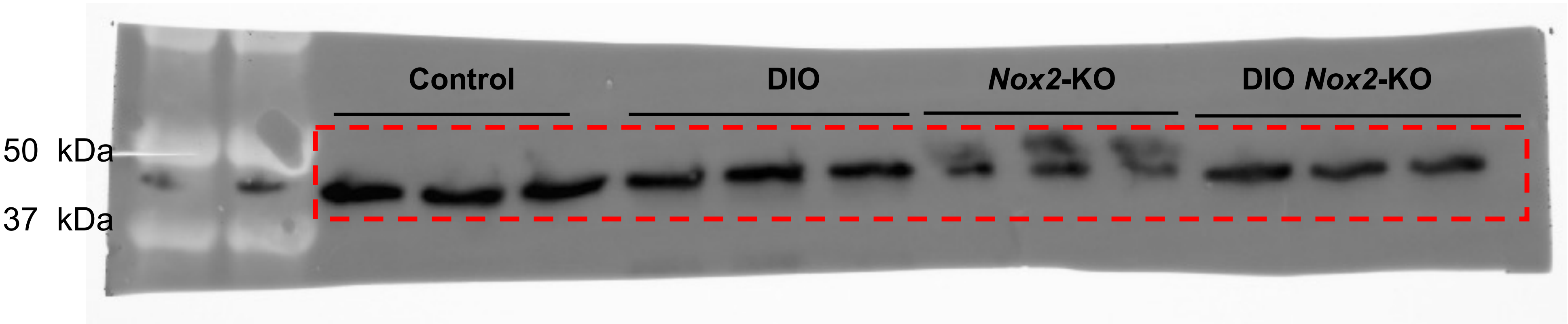

Blot 1- Kir3.1

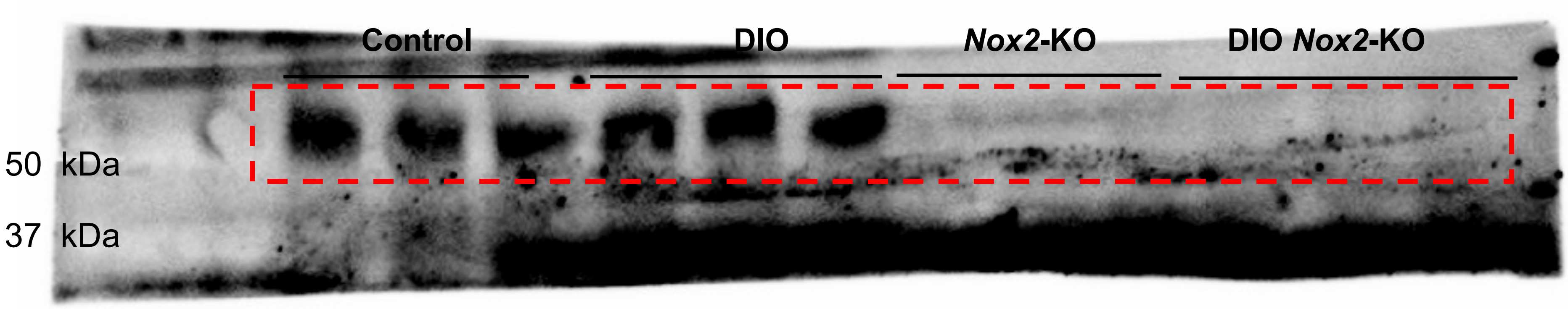

Red box refers to blot used in figure

Unedited Figure S4G- Anp blots

Actin -1

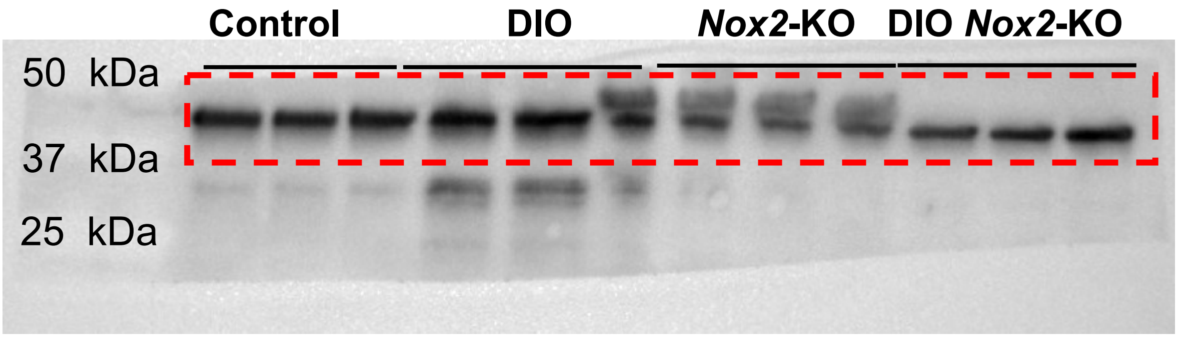

Actin -2

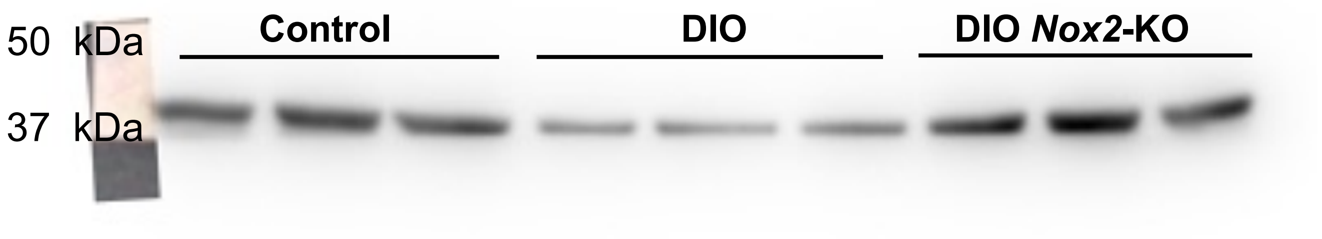

Anp-1

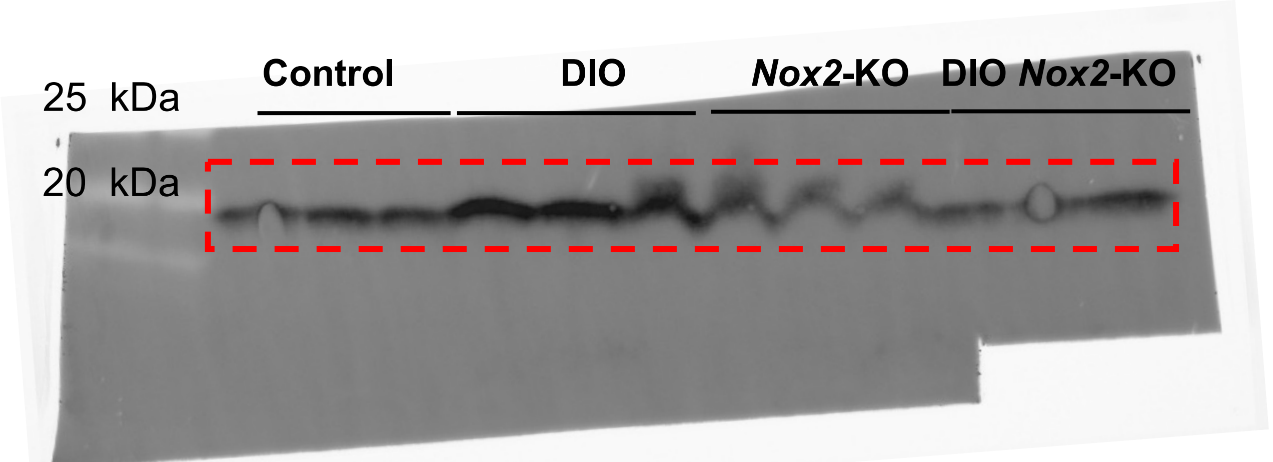

Anp- 2

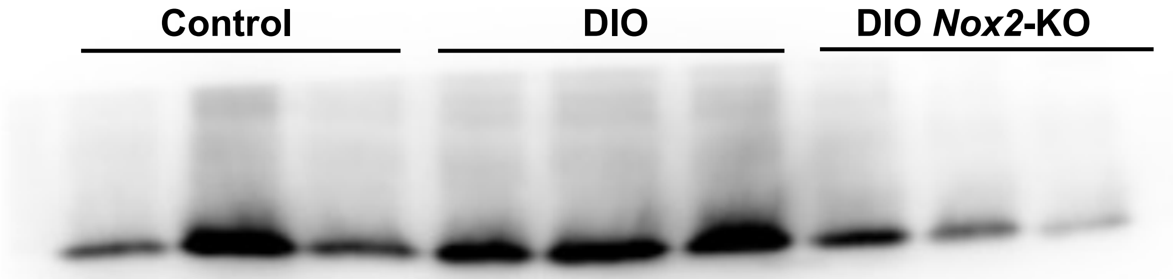

Red box refers to blot used in figure

Unedited Figure S9A- cTnI blots

Actin -1

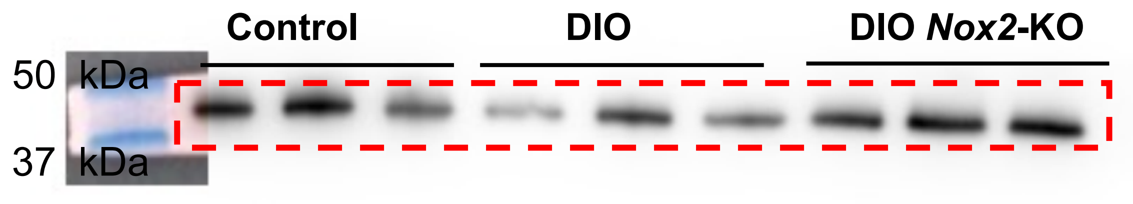

Actin -2

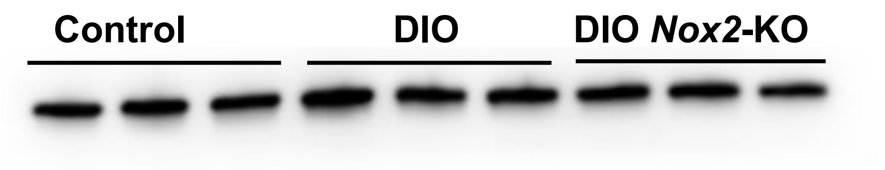

cTnI-1

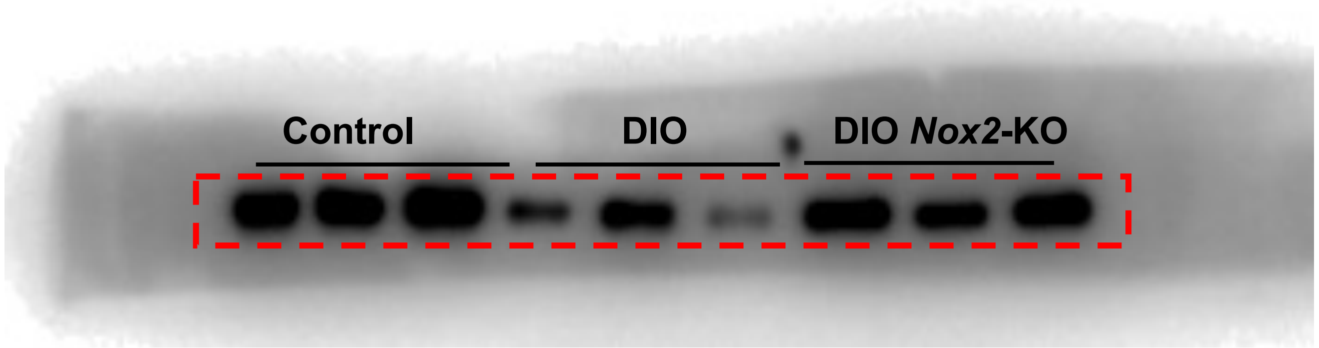

cTnI- 2

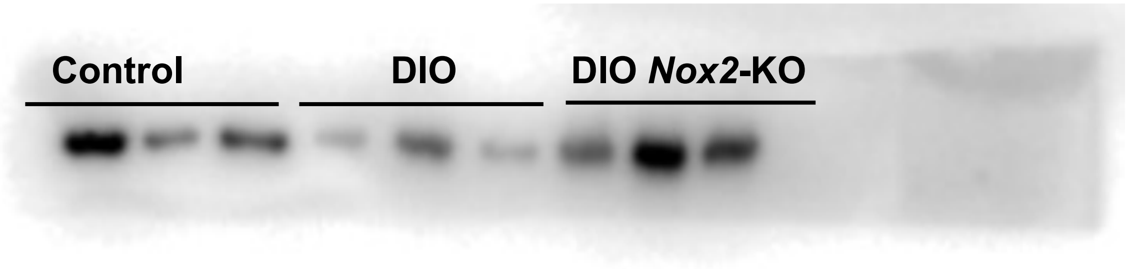

Red box refers to blot used in figure

Unedited Figure S9B- cTnT blots

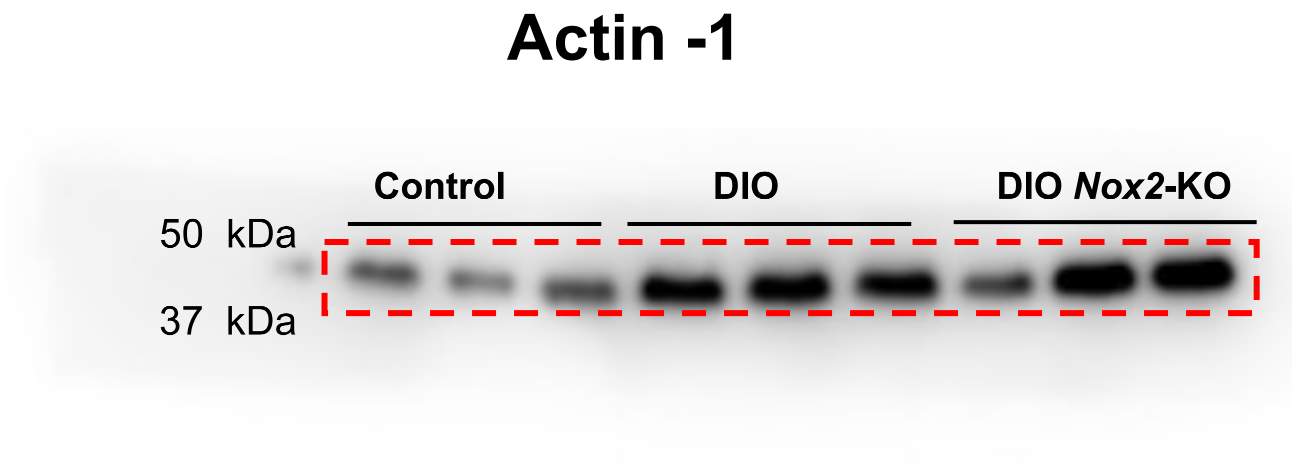

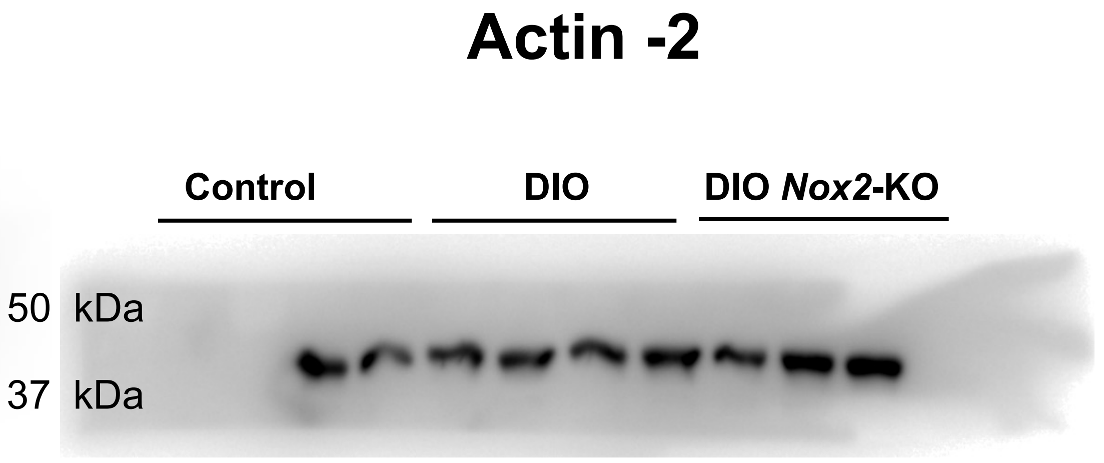

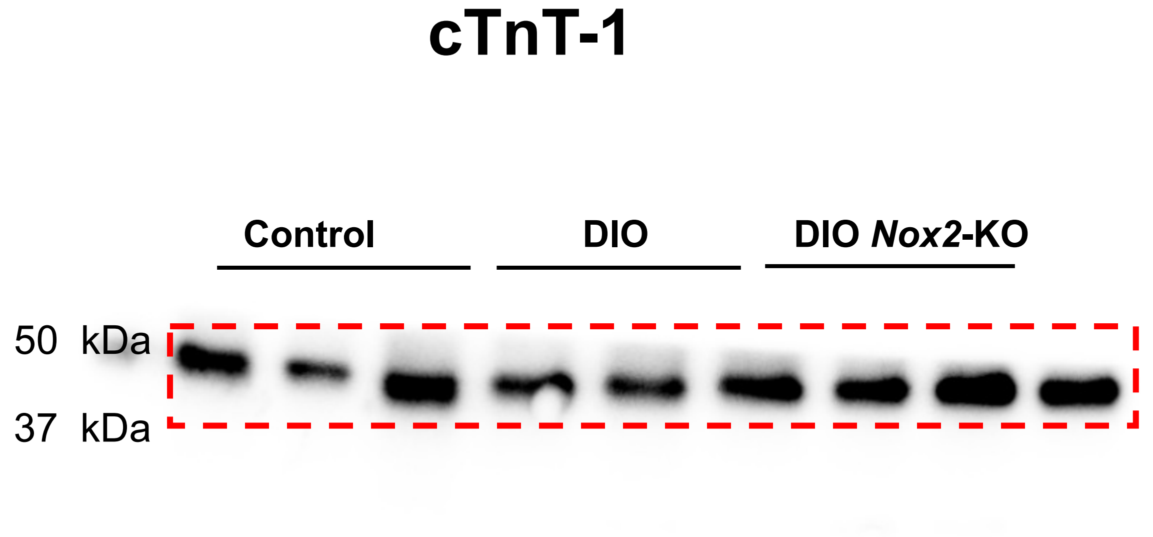

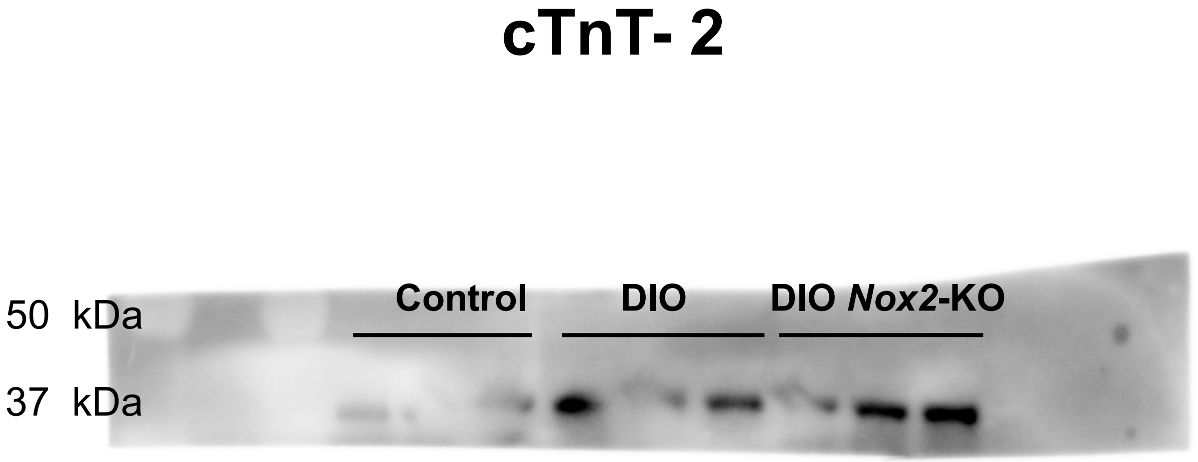

Red box refers to blot used in figure

Unedited Figure S9C- Mybpc3 blots

Actin -1

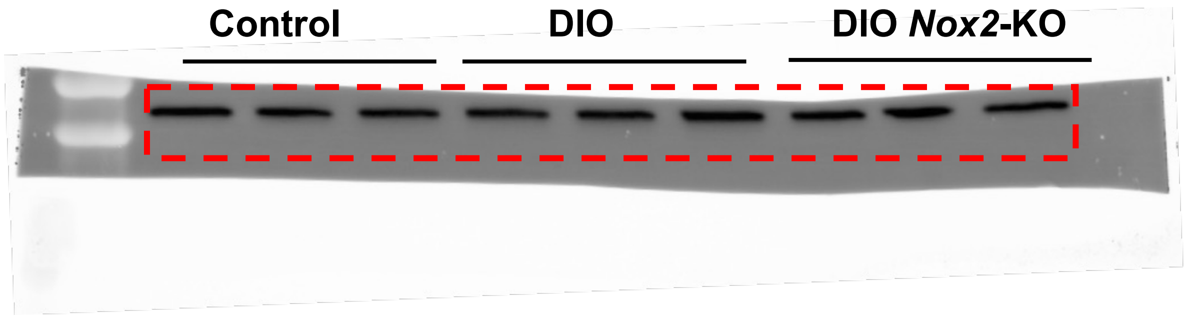

Actin -2

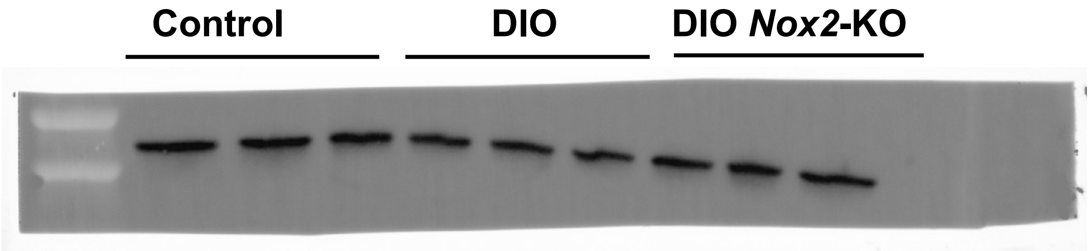

Mybpc3-1

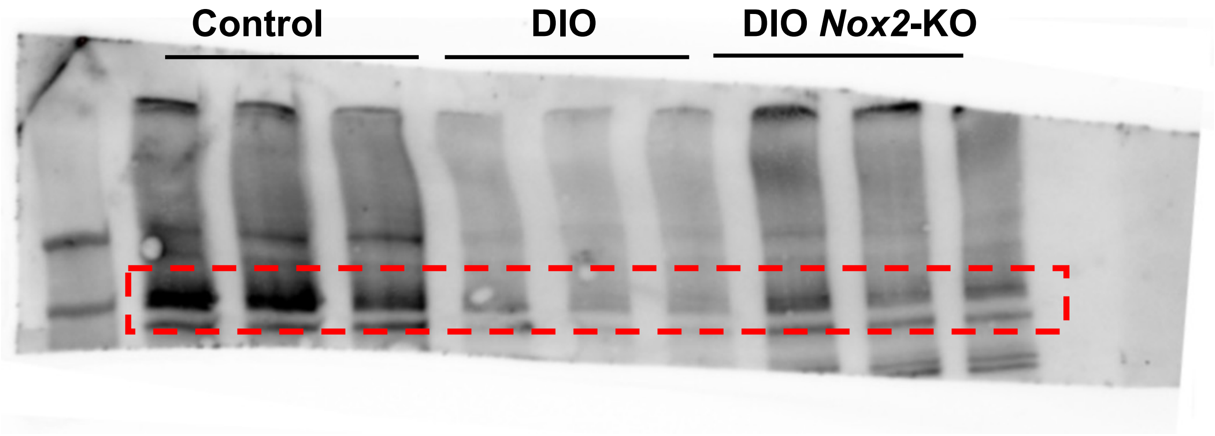

Mymo1- 1

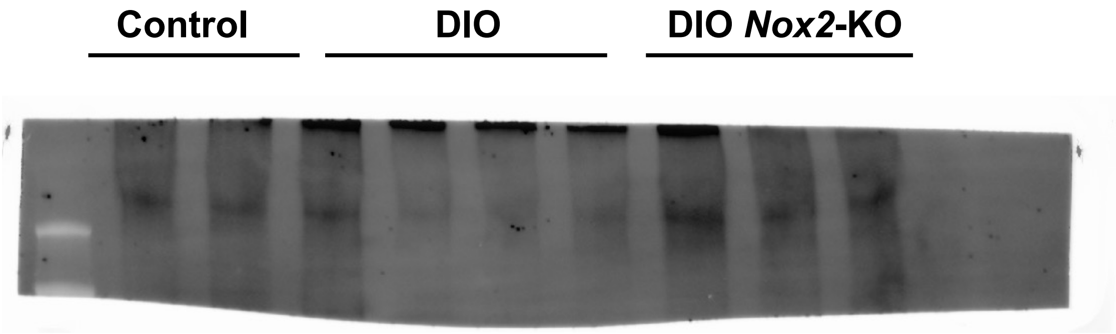

Red box refers to blot used in figure

Unedited Figure S9D- Myom1 blots

Actin -1

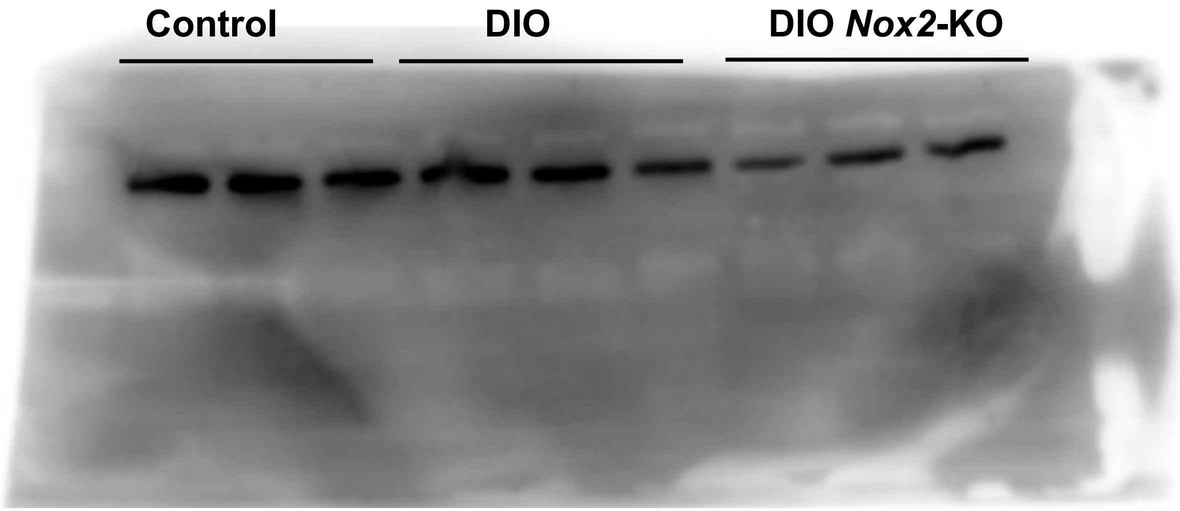

Actin -2

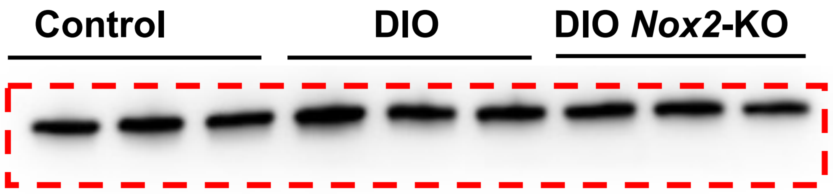

Myom1-1

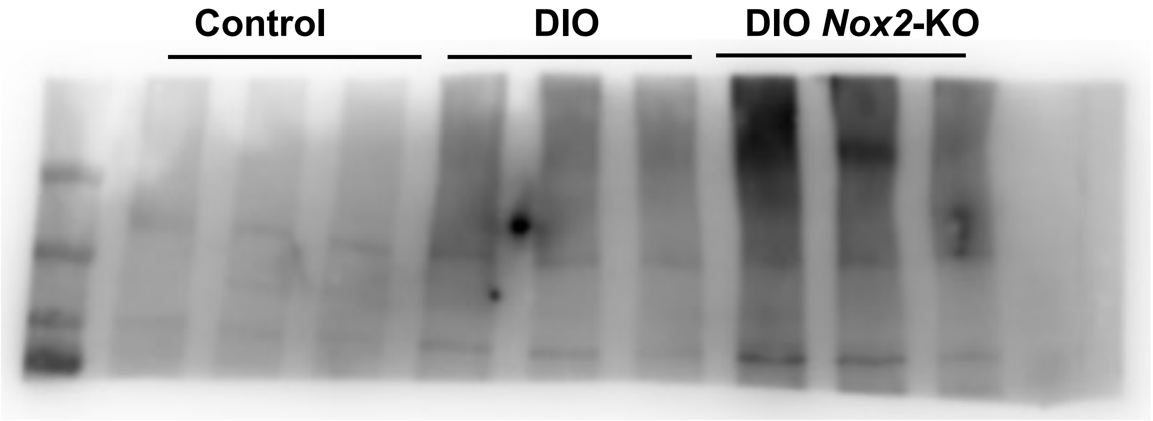

Mymo1- 1

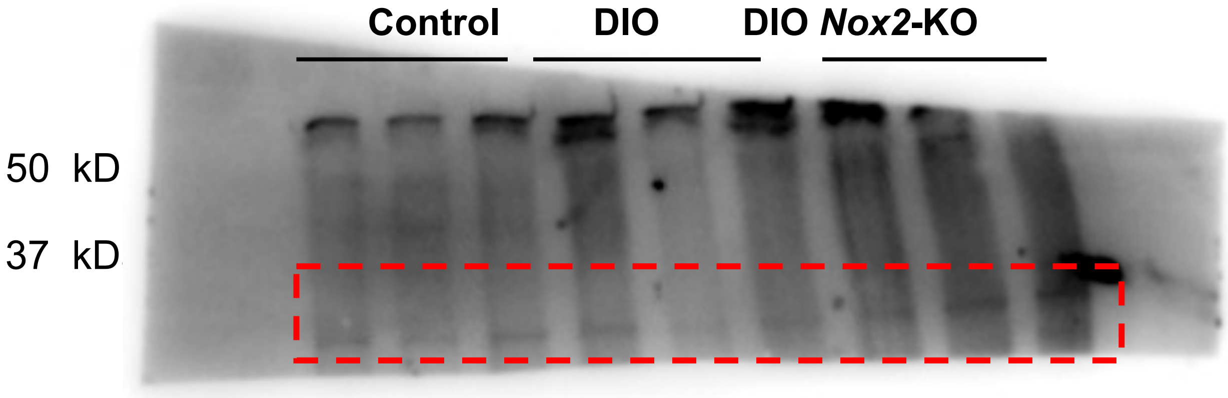

Red box refers to blot used in figure

Unedited Figure S9E- Myl7 blots

Actin -1

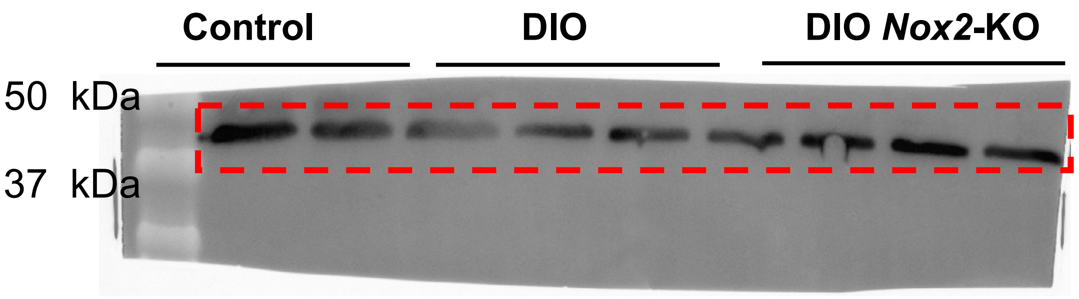

Actin -2

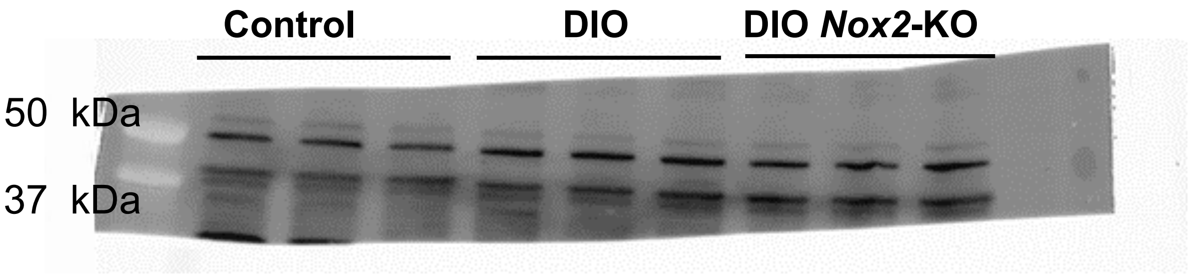

Myl7-1

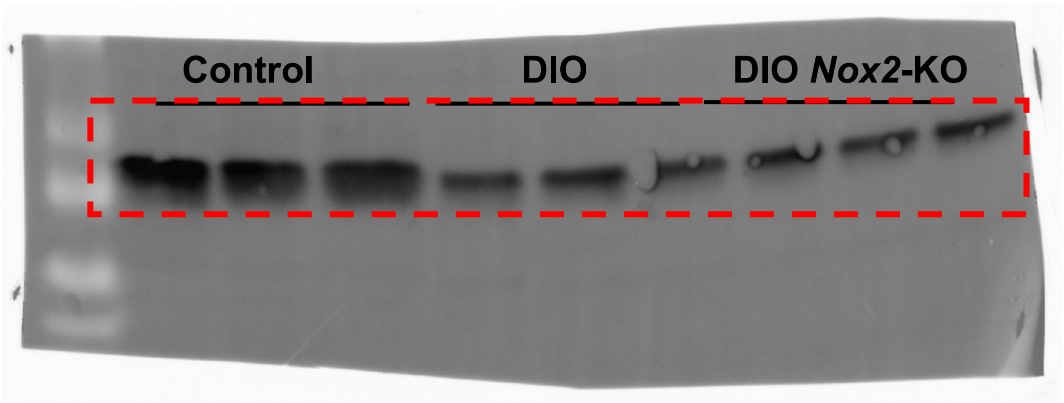

Myl7- 2

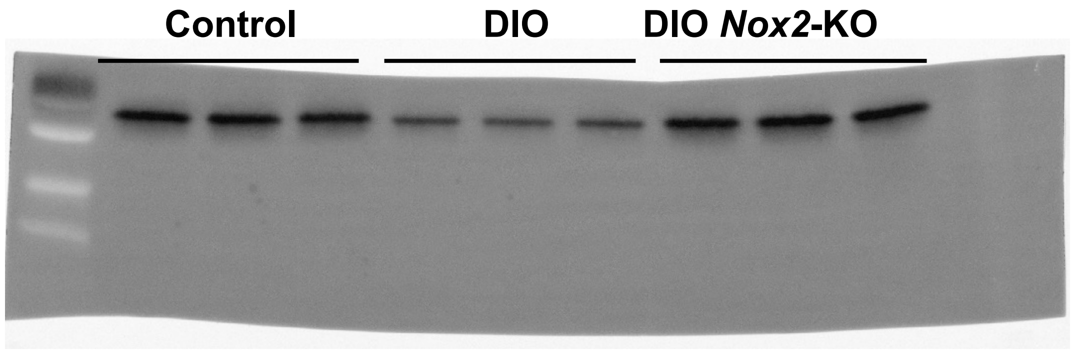

Red box refers to blot used in figure

Unedited Figure S9F- Mlc2v blots

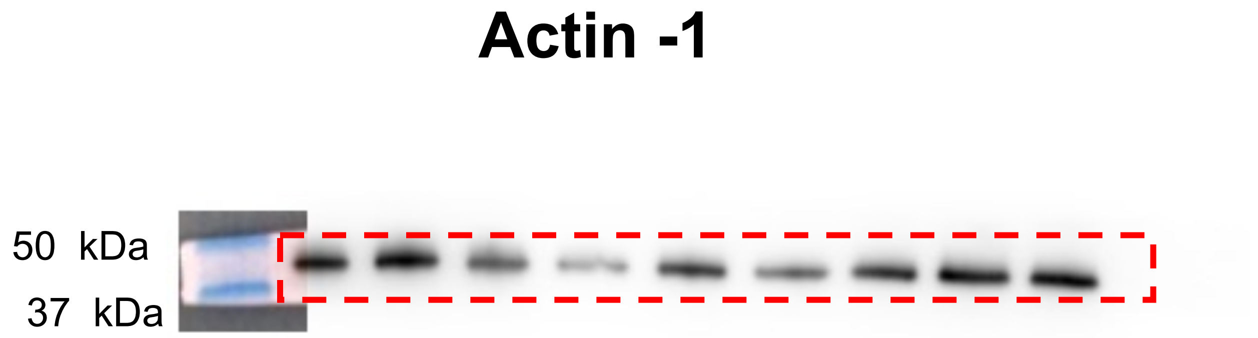

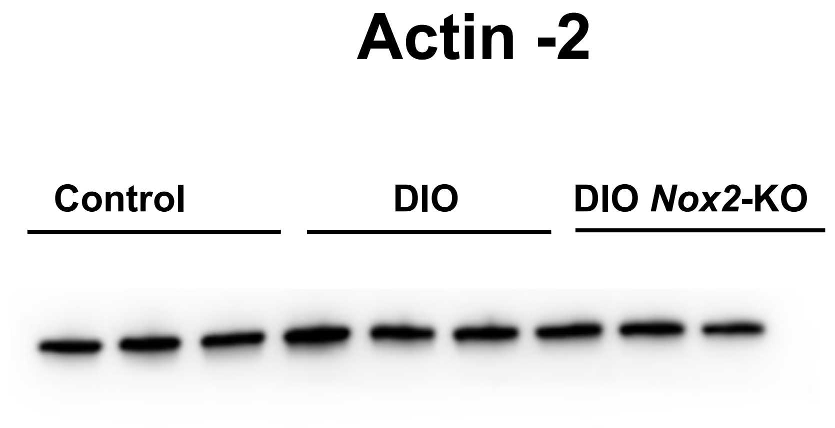

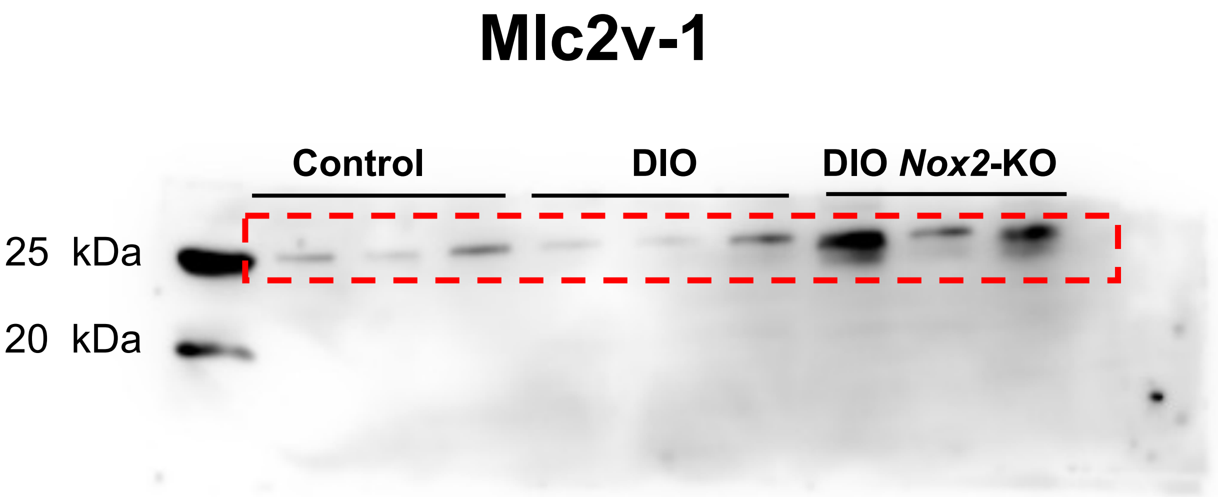

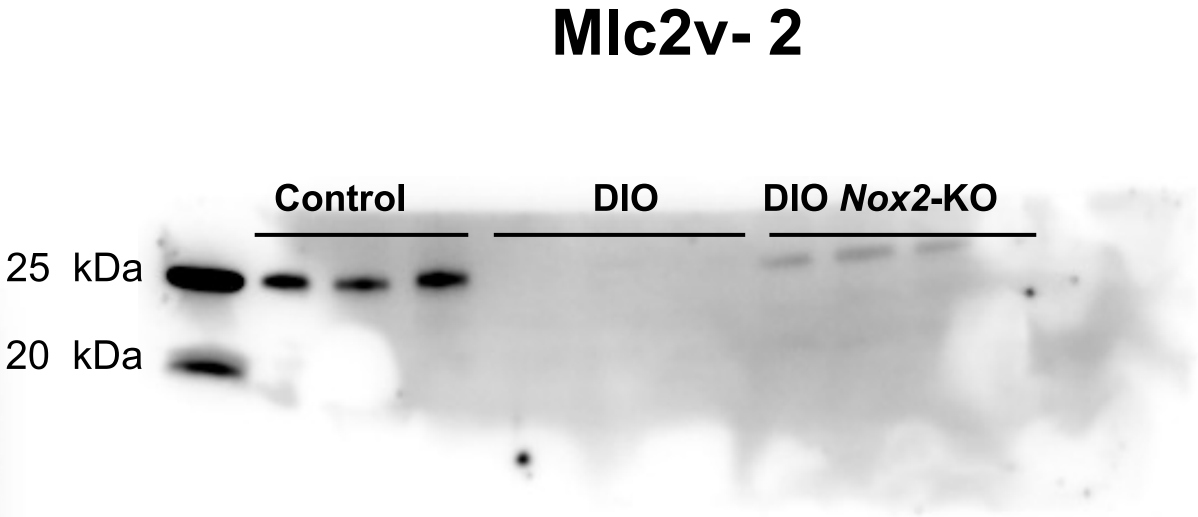

Red box refers to blot used in figure

Unedited Figure S9G- Cpt1α blots

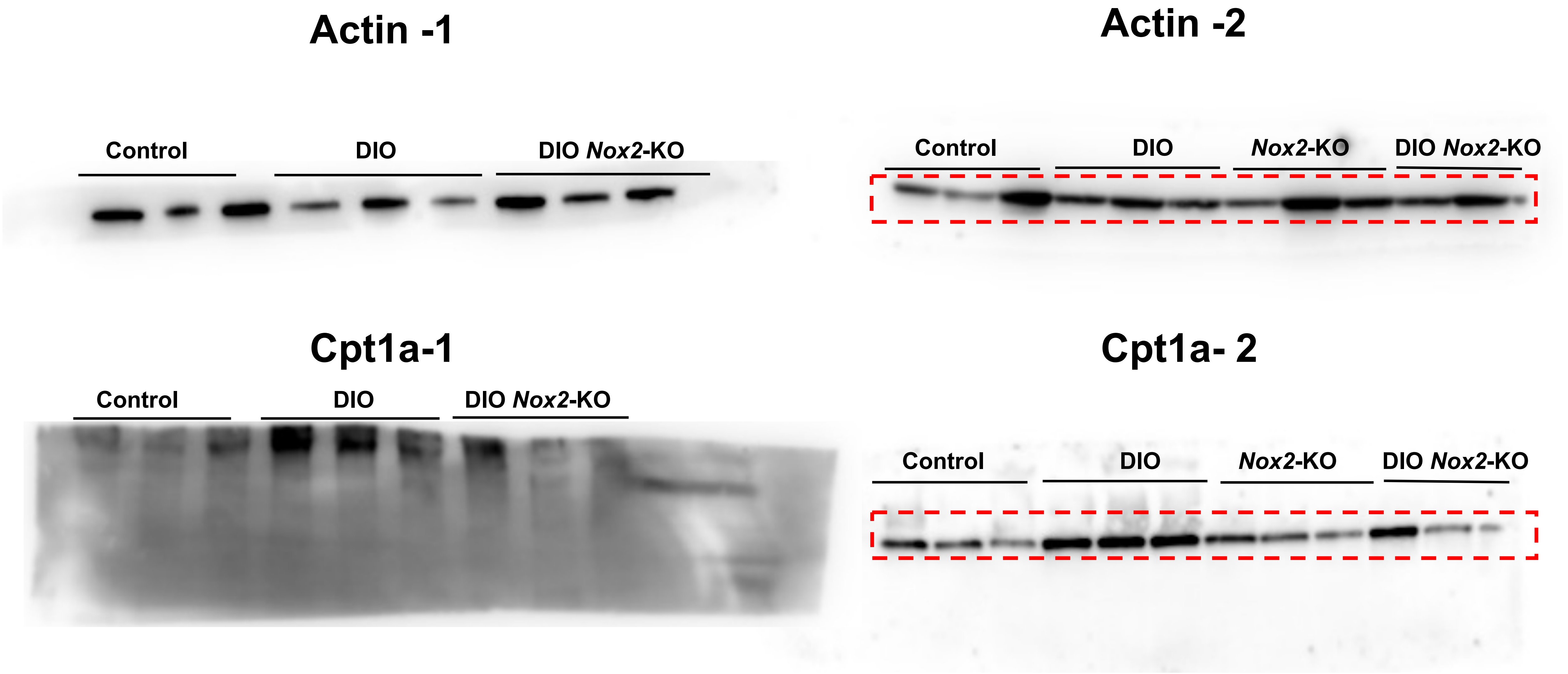

Red box refers to blot used in figure

Unedited Figure S9H- Pparα blots

Actin -1

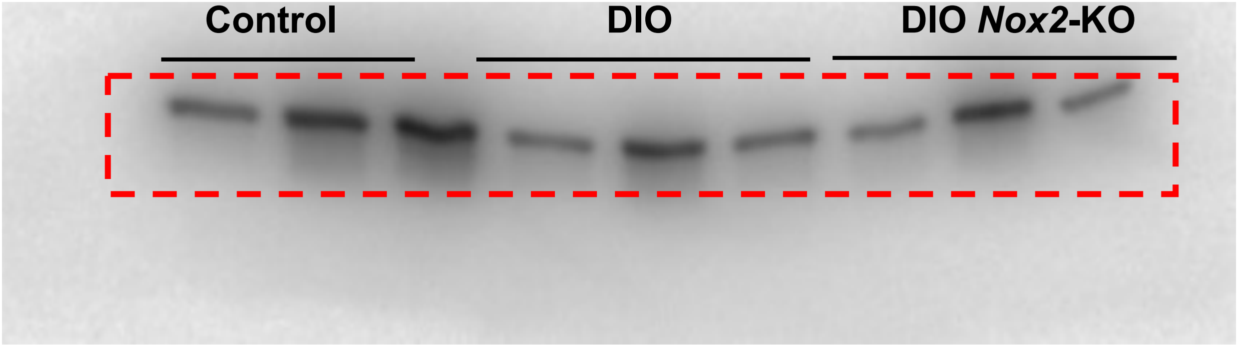

Actin -2

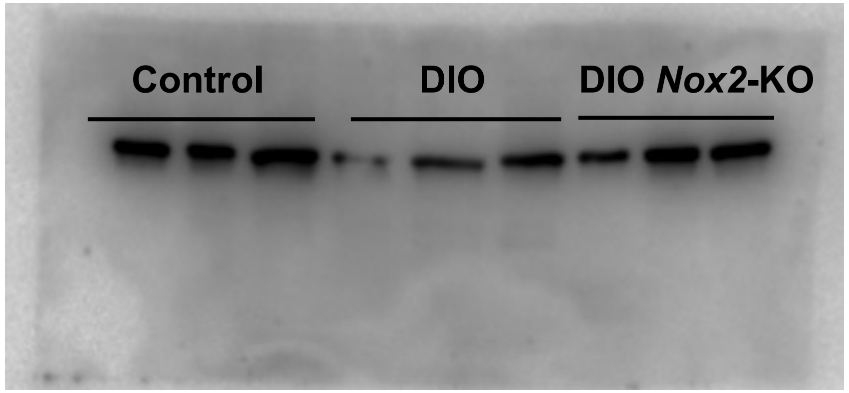

Pparα-1

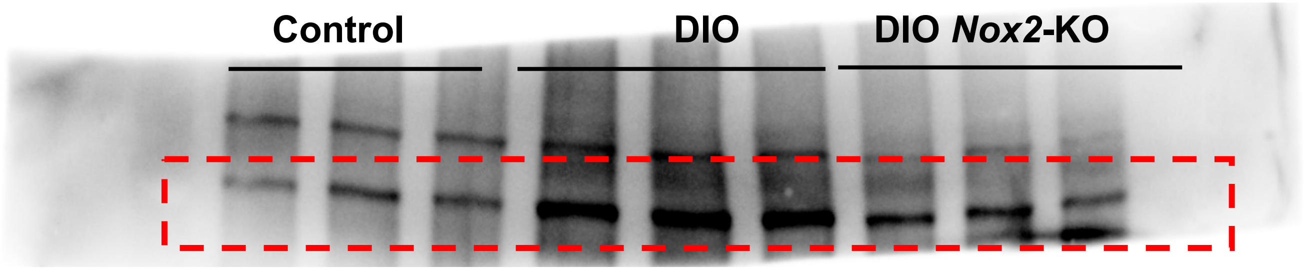

Pparα- 2

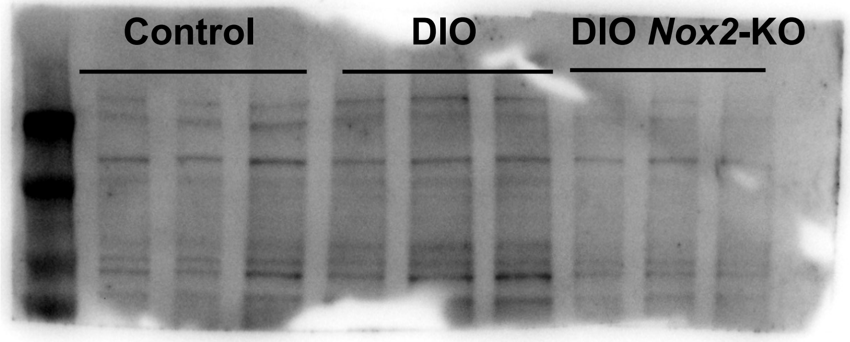

Red box refers to blot used in figure

Unedited Figure S9I- Fabp3 blots

Actin -1

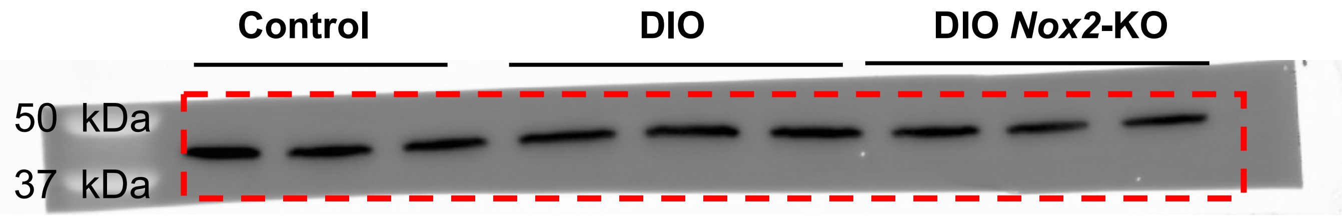

Actin -2

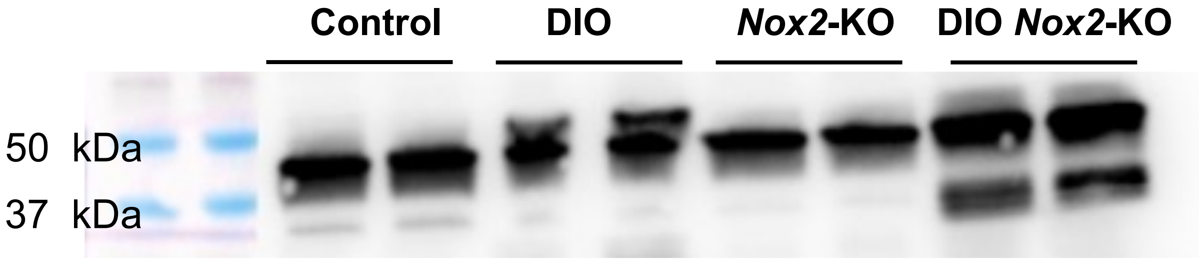

Fabp3-1

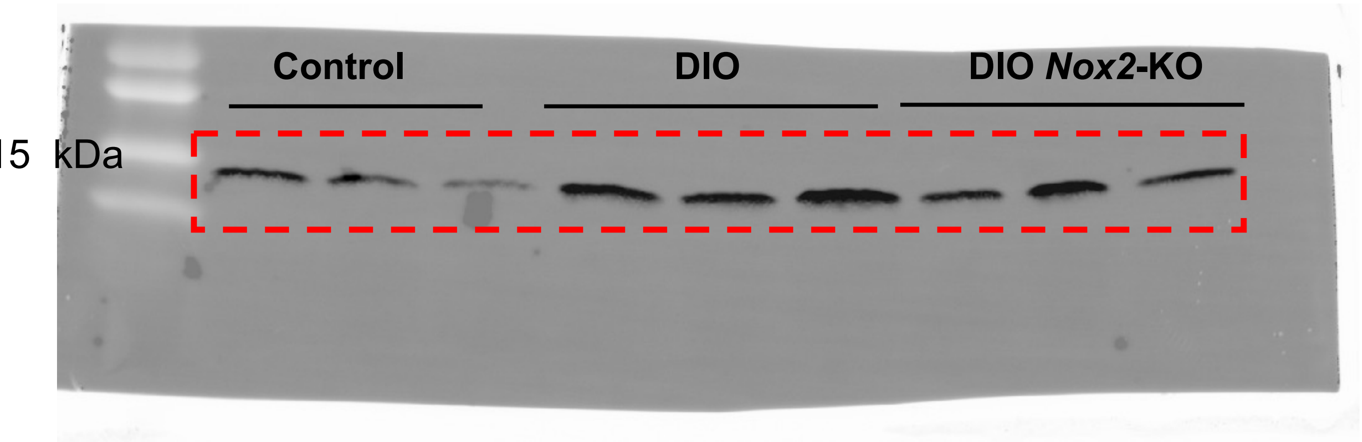

Fabp3- 2

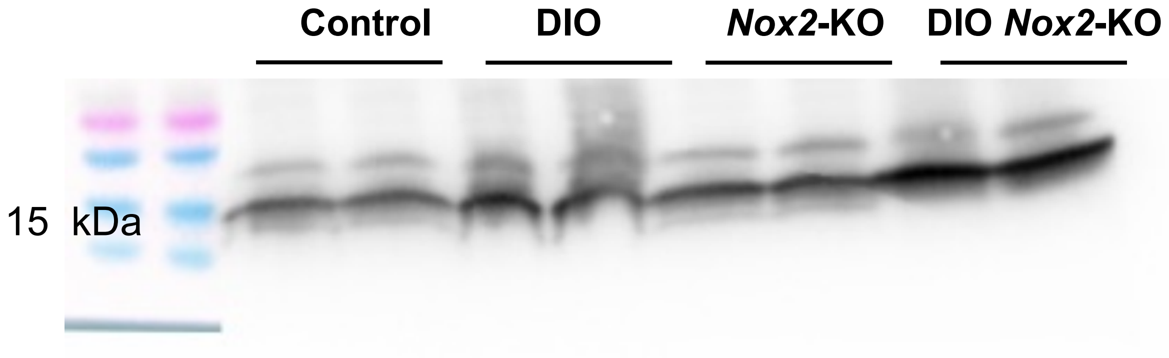

Red box refers to blot used in figure
